# Parkinson’s disease is associated with DNA methylation levels in human blood and saliva

**DOI:** 10.1186/s13073-017-0466-5

**Published:** 2017-08-30

**Authors:** Yu-Hsuan Chuang, Kimberly C. Paul, Jeff M. Bronstein, Yvette Bordelon, Steve Horvath, Beate Ritz

**Affiliations:** 1 Department of Epidemiology, Fielding School of Public Health, University of California Los Angeles, 650 Charles E. Young Drive, Box 951772, Los Angeles, CA 90095-1772 USA; 2 Department of Human Genetics, David Geffen School of Medicine, University of California Los Angeles, 695 Charles E. Young Drive South, Box 708822, Los Angeles, CA 90095-7088 USA; 3 Department of Biostatistics, Fielding School of Public Health, University of California Los Angeles, Los Angeles, CA 90095 USA; 4 Department of Neurology, David Geffen School of Medicine, University of California Los Angeles, Los Angeles, CA 90095 USA; 5 Department of Environmental Health, Fielding School of Public Health, University of California Los Angeles, Los Angeles, CA 90095 USA

**Keywords:** DNA methylation, Epigenomics, Parkinson’s disease, Mitochondrial dysfunction, Immune system, Cytoskeleton, Blood cell counts, Saliva, WGCNA, Bioinformatics

## Abstract

**Background:**

Several articles suggest that DNA methylation levels in blood relate to Parkinson’s disease (PD) but there is a need for a large-scale study that involves suitable population based controls. The purposes of the study were: (1) to study whether PD status is associated with DNA methylation levels in blood/saliva; (2) to study whether observed associations relate to blood cell types; and (3) to characterize genome-wide significant markers (“CpGs”) and clusters of CpGs (co-methylation modules) in terms of biological pathways.

**Methods:**

In a population-based case control study of PD, we studied blood samples from 335 PD cases and 237 controls and saliva samples from another 128 cases and 131 controls. DNA methylation data were generated from over 486,000 CpGs using the Illumina Infinium array. We identified modules of CpGs (clusters) using weighted correlation network analysis (WGCNA).

**Results:**

Our cross-sectional analysis of blood identified 82 genome-wide significant CpGs (including cg02489202 in *LARS2 p* = 8.3 × 10^–11^ and cg04772575 in *ABCB9 p* = 4.3 × 10^–10^). Three out of six PD related co-methylation modules in blood were significantly enriched with immune system related genes. Our analysis of saliva identified five significant CpGs. PD-related CpGs are located near genes that relate to mitochondrial function, neuronal projection, cytoskeleton organization, systemic immune response, and iron handling.

**Conclusions:**

This study demonstrates that: (1) PD status has a profound association with DNA methylation levels in blood and saliva; and (2) the most significant PD-related changes reflect changes in blood cell composition. Overall, this study highlights the role of the immune system in PD etiology but future research will need to address the causal structure of these relationships.

**Electronic supplementary material:**

The online version of this article (doi:10.1186/s13073-017-0466-5) contains supplementary material, which is available to authorized users.

## Background

There is a growing interest in exploring the role of DNA methylation in Parkinson’s disease (PD). Conflicting evidence has been presented regarding the hypomethylation of intron 1 in the *SNCA* of PD patients [1–3]. A small epigenome-wide association study (EWAS) of 30 PD patients and 15 controls identified two loci, *FANCC* cg14115740 and *TNKS2* cg11963436, as hypermethylated in patients [4] and was able to replicate this with bisulfite sequencing in a targeted analysis of 219 PD patients and 223 controls. While these results suggest that PD status might be associated with changes in DNA methylation levels in blood, there is a danger of false-positive findings due to small sample sizes. In general, DNA methylation studies are plagued by hidden biases including systematic differences between cases and controls in terms of DNA collection, DNA extraction, DNA storage, or bisulfite conversion. We have previously shown that PD cases differ significantly from controls in terms of blood cell composition (notably granulocytes [5]) which might confound the relationship between PD status and DNA methylation levels.

There is a critical need for rigorous large-scale studies that involve DNA samples from PD cases and suitable controls that were collected and stored in an identical fashion. Here we leverage our community-based case control study of PD to identify CpGs that relate to PD status in blood or saliva. We also apply a systems biology data analysis method (weighted correlation network analysis [6, 7]) to identify PD related modules, i.e. clusters of CpGs. WGCNA is a widely used method because: (1) it circumvents the multiple comparison problem inherent in large scale genomic data; and (2) it amplifies the underlying biological signal in functional enrichment studies [8].

## Methods

### Study population

Our study involved blood samples from 508 individuals of European ancestry (289 PD patients and 219 controls) and 64 individuals of Hispanic ancestry (46 PD patients and 18 controls) who provided blood samples for DNA extraction in the Parkinson’s Environment and Genes (wave 1 known as PEG1) study we conducted during 2000–2007 in central California. PEG2 study enrolment started in 2010 (ongoing) and 128 PD patients and 131 controls provided saliva samples (Additional file 1: Table S1). We selected saliva samples from among PEG2 participants matched on age, sex, and race for these methylation analyses. PD patients enrolled in PEG1 were recently diagnosed (within three years) and in PEG2 on average four years before they were examined by UCLA movement disorder specialists. Movement disorder specialists (JB, YB) applied UK Brain Bank and Gelb diagnostic criteria for diagnosing idiopathic PD (iPD) and collected bio-samples [9–11]. In PEG1, eligible PD patients had to be residents of Fresno, Kern, or Tulare Counties and lived in California for at least five years; patients were identified by neurologists, large medical groups, or public service announcements [12, 13]. In PEG2, PD patients were identified through the California PD Registry operating in the same counties [14]. Population controls were identified using Medicare lists and residential tax assessor records. Controls who provided saliva samples were randomly selected from clusters of five neighboring households study staff approached in person; one eligible household member was allowed to enroll (see [12, 14] for more recruitment details).

Demographic information, lifestyle factors, and medication use were collected in standardized interviews, including lifetime information on cigarette smoking and coffee/tea consumption, allowing us to calculate total pack-years and average daily coffee/tea consumption. We also calculated levodopa equivalent doses at time of blood draw based on patient reported PD medication histories [15].

### DNA methylation profiling

DNA was extracted from peripheral whole blood and saliva. We used the Illumina HumanMethylation450 BeadChip to determine methylation profiles from over 486,000 CpGs. The raw methylation data (beta values) were preprocessed using the background normalization method from the Genome Studio software. Sex concordance was confirmed.

### Statistical analysis

We regressed individual CpGs on potential confounding variables (age, sex, and optionally blood cell counts) and formed residuals. Replacing DNA methylation levels by residuals is a widely used method for conditioning out confounders (see e.g. [16, 17]). In our epigenome-wide association analysis (EWAS), we related each CpG separately to PD status. Toward this end, we related the adjusted DNA methylation levels to PD status using the R function “standardScreening” in the WGCNA R package. The R function produces a t-test statistic and corresponding two-sided *p* values. We also calculated empirical *p* values by permuting associations between DNA methylation levels in blood and PD status to strengthen results. Abundance measures of blood cell types were estimated with the Houseman algorithm in the minfi R package and the epigenetic clock software [18–20]. To adjust for multiple comparisons (about 486,000 CpGs on the Illumina array), we used the stringent Bonferroni correction method resulting in a genome-wide significance threshold of 0.05/500,000 = 10^–7^. The Bonferroni correction method, which applies to independent hypothesis tests, is highly conservative in light of the fact that the CpGs tend to be highly correlated as can be seen from our WGCNA analysis.

To investigate the biological function shared by genome-wide significant (*p* < 10^–7^) CpGs, we assessed the functional enrichment of genes that are located near the respective CpGs according to the probe annotation table from Illumina. Toward this end, we used the online enrichment tool from the Database for Annotation, Visualization and Integrated Discovery (DAVID v.6.7).

We also stratified by ethnicity since DNA methylation levels can be associated with ethnicity [21]. To combine the results from individuals of European and Hispanic ancestry, we used Stouffer’s meta-analysis resulting in a Z statistic, *meta.Z*, and a corresponding two-sided *p* value, *meta.P*.

We used a systems biologic analysis approach based on WGCNA [6, 7] to identify clusters of highly correlated CpGs (co-methylation modules) in an unsupervised manner, i.e. agnostic of gene ontology. Toward this end, we focused on the 250,000 CpGs with the highest variance across individuals (ignoring PD status). Modules were calculated using the blockwise module function and a robust correlation measure (bi-weight mid-correlation). The CpGs inside each module were represented by a weighted average, the module eigengene (ME), which is formally defined as the first principal component. The MEs were correlated with PD status, chronological age, blood cell count estimates, and other sample characteristics.

To gain insights into the biology underlying PD-related modules, we carried out a pathway enrichment analysis of genes using DAVID. For modules with more than 3000 CpGs, we focused on the 3000 CpGs with highest module membership values.

## Results

**Fig. 1:**
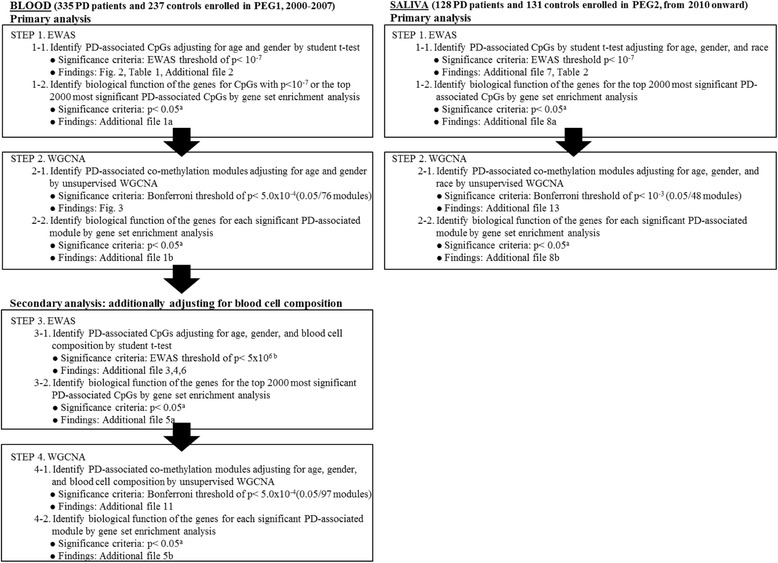
Flowchart of analysis process. EWAS and WGCNA analyses in blood and saliva separately. ^a^Bonferroni, Benjamini corrected *p*values, and FDR were also provided. ^b^No CpG with EWAS *p*values <10^-7^ was found; therefore, we relaxed the significance criteria

An overview of our statistical analysis is presented in Fig. 1. Age, sex, and blood cell counts can have a profound effect on DNA methylation levels. To adjust for these possible confounders, we carried out two distinct pre-processing steps that correspond to separate sets of confounding variables. In our primary analysis, we only adjusted for differences in age and sex because we hypothesized that PD-related changes in DNA methylation might reflect changes in blood cell composition and immune system functioning [5]. In a secondary analysis, we also adjusted DNA methylation levels for differences in blood cell composition in order to find cell-intrinsic changes associated with PD.

### Epigenome-wide association study

#### DNA methylation in blood without adjustment for blood cell composition

**Fig. 2:**
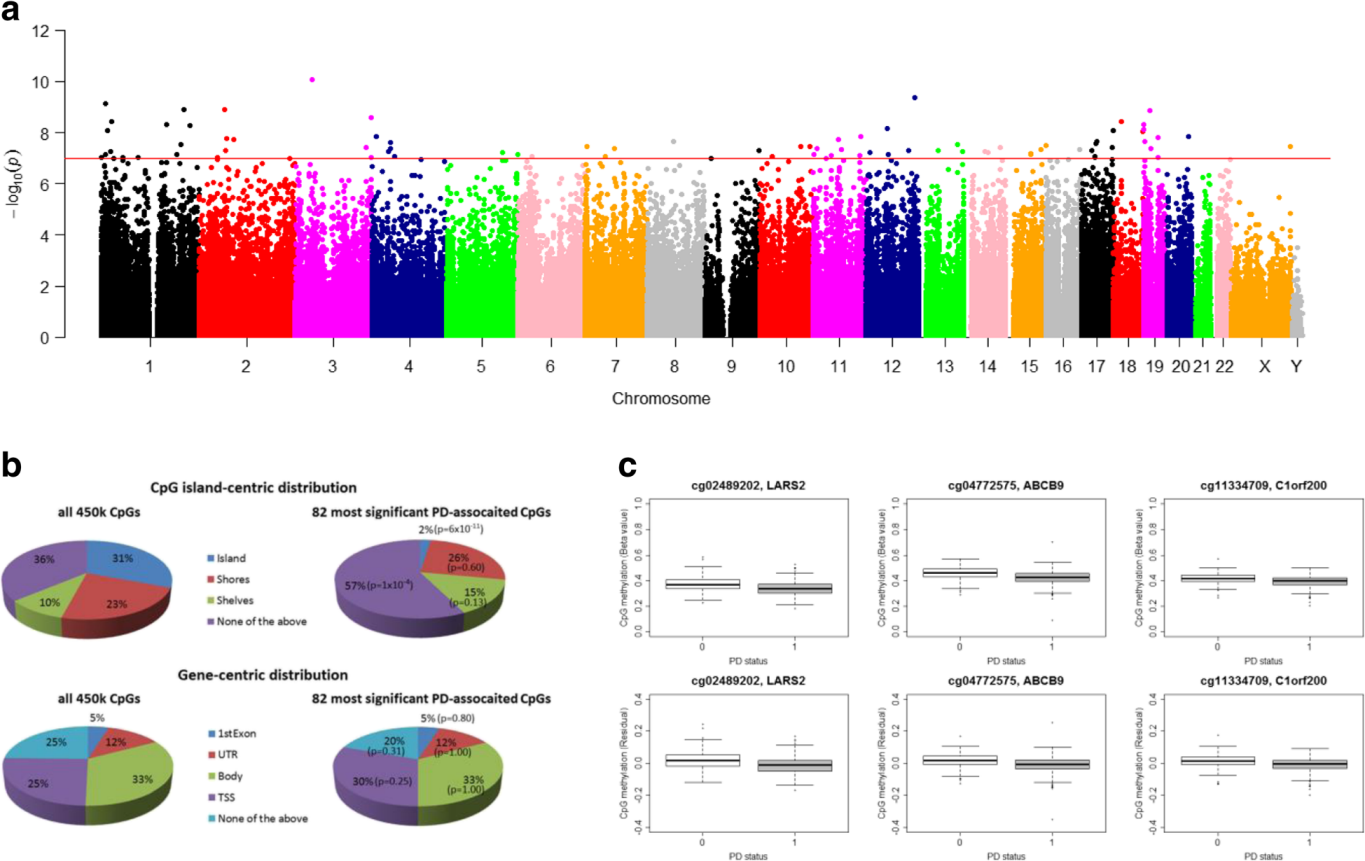
EWAS results for PD and blood-based DNA methylation analyses without cell composition adjustment. Differential methylation associated with PD status in 508 PEG1 subjects of European ancestry adjusting for age and gender. **a** Manhattan plot of *p* values adjusted for age and gender (*red line*: *p* value threshold of 10^–7^). **b** Distributions of CpGs relative to CpG island and gene regions for all 450,000 CpGs on the microarray and the 82 most significant coffee-associated CpGs listed in Table 1. **c** Distribution of DNA methylation levels for the top three most significant PD-associated CpGs by PD status (1 = PD)

In our primary analysis, we related PD status with DNA methylation levels that were adjusted for age and sex but not for blood cell counts. We identified 82 genome-wide significant CpGs (*p* < 10^–7^; Fig. 2a) in individuals of European ancestry. A study of the chromosomal location revealed that only 2% of the CpGs were located in a CpG island compared to an expected proportion of 31% (Fig. 2b). The most significant PD-related CpG, cg02489202 (located in the mitochondrial gene *LARS2*), tends to be hypomethylated in PD cases (*p* = 8.3 × 10^–11^; Table 1). Similarly, hypomethylation in PD cases can be observed for two highly significant CpGs in immune-related genes cg04772575 in *ABCB9* (*p* = 4.3 × 10^–10^) and cg11334709 in *C1orf200* (*p* = 7.5 × 10^–10^). Notably, results among the remaining 79 CpGs include two CpGs in the *AZU1* gene, two Alzheimer’s disease (AD)-related genes, *CLSTN1* and *CALM2*, and a second mitochondria-related gene, *MIR1977*.

**Table 1:** List of 82 PD-associated CpGs in blood-based analyses with blood cell composition adjustment

	CpG	Gene	Chr.	Position (bp)	Relation to UCSC CpG island	Gene region	SNPs	SNPs_10	cor	*p* value
1	cg02489202	LARS2	3	45505334		Body			−0.30	8.34E-11
2	cg04772575	ABCB9	12	123431865		Body	rs73408862		−0.28	4.33E-10
3	cg11334709	C1orf200	1	9716019	S_Shelf	TSS1500			−0.28	7.47E-10
4	cg17491368		1	211779938					−0.28	1.23E-09
5	cg16240816		2	65861662			rs80084148		0.28	1.25E-09
6	cg19879906		19	16392219	N_Shelf				−0.28	1.40E-09
7	cg08704934	C3orf21	3	194826585		Body		rs6799614	−0.27	2.64E-09
8	cg09993145	RUNX3	1	25291905		TSS1500			0.27	3.59E-09
9	cg01152726	LAMA3	18	21452844		TSS200			0.27	3.64E-09
10	cg09032544	CD247	1	167487295		Body			0.27	4.69E-09
11	cg10752406	AZU1	19	827776		TSS200			−0.27	4.96E-09
12	cg26341831	TMEM63A	1	226036279		Body			0.27	5.21E-09
13	cg01213231	ITGA5	12	54806218		Body			−0.26	6.99E-09
14	cg16643542	AZU1	19	827843		1stExon	rs34124897		−0.26	7.37E-09
15	cg21577598	CCDC57	17	80084751	N_Shore	Body			0.26	8.11E-09
16	cg26793227	EPHA2	1	16483658	S_Shore	TSS1500	rs57602506		−0.26	8.17E-09
17	cg16270399	LOC284276	18	74257894		Body			−0.26	8.95E-09
18	cg22358291	WDR1	4	10101553		Body			0.26	1.38E-08
19	cg26681770	PMEPA1	20	56247302	Island	5′UTR	rs36008751		−0.26	1.40E-08
20	cg01657758	SORL1	11	121349740		Body			−0.26	1.46E-08
21	cg24168413	FXYD1	19	35630388	N_Shore	5′UTR	rs77395635		−0.26	1.53E-08
22	cg26474124		2	70368457	N_Shore		rs11685382		−0.26	1.66E-08
23	cg12792363	LGALS12	11	63274030		Body			−0.26	1.79E-08
24	cg12500949		2	88357920	S_Shelf				0.26	1.81E-08
25	cg16580197		8	67841925	S_Shelf				−0.26	2.20E-08
26	cg19709355	RARA	17	38504102	S_Shelf	Body			−0.26	2.27E-08
27	cg24339704	GNG7	19	2529022	S_Shelf	5′UTR	rs740054		−0.26	2.32E-08
28	cg02600394	TXK	4	48136234		5′UTR			0.26	2.44E-08
29	cg23207054	CSF3	17	38171530		TSS200			−0.25	2.79E-08
30	cg14659511	DOCK9	13	99668433		Body			0.25	2.96E-08
31	cg19081101	CHI3L1	1	203156625		TSS1500			−0.25	3.05E-08
32	cg20357538	CHSY1	15	101777761		Body		rs11855006	−0.25	3.31E-08
33	cg26279840	IKBKG	X	153770418		TSS200			−0.25	3.35E-08
34	cg17879101	FAM53B	10	126329354		Body			−0.25	3.37E-08
35	cg27640064	FOXK1	7	4752983		Body		rs77026339	−0.25	3.50E-08
36	cg02505177	MGEA5	10	103574626	N_Shelf	Body			0.25	3.55E-08
37	cg23189692	EIF4G1	3	184050393	N_Shelf	Body			−0.25	3.83E-08
38	cg15209885	CBX2	17	77753199	S_Shore	Body	rs72231015		−0.25	3.89E-08
39	cg19011001	ITPK1	14	93539613		Body			−0.25	3.97E-08
40	cg17984638	TXK	4	48136452		TSS200			0.25	4.02E-08
41	cg17173442	RFXANK	19	19305340	S_Shore	Body			−0.25	4.10E-08
42	cg26489413	AMPD3	11	10476976		1stExon			−0.25	4.15E-08
43	cg20720686	POR	7	75582881		5′UTR	rs41295375		−0.25	4.16E-08
44	cg08069287		11	72868833					−0.25	4.42E-08
45	cg26963632		16	85558148					−0.25	4.49E-08
46	cg14023999		15	90543224	N_Shore				−0.25	4.68E-08
47	cg02861056	PLEK	2	68592345		1stExon			−0.25	4.86E-08
48	cg24185397		17	25659609	N_Shore				−0.25	4.90E-08
49	cg13060970	PLEK	2	68592349		1stExon			−0.25	4.93E-08
50	cg21252105		9	139459307					−0.25	5.07E-08
51	cg01752594	DLEU2	13	50696070	N_Shore	Body			−0.25	5.09E-08
52	cg14642045	UNG	12	109538736	S_Shelf	Body	rs3219221		−0.25	5.17E-08
53	cg08400494	CARS2	13	111318490		Body			−0.25	5.34E-08
54	cg09298313		14	55569959					−0.25	5.36E-08
55	cg15961455		1	23590501					−0.25	5.38E-08
56	cg16000989	DCAF4L1	4	41983716	N_Shore	5′UTR			−0.25	5.54E-08
57	cg14001486	PRKCH	14	61801201		Body			0.25	5.92E-08
58	cg12810837	CLEC2D	12	9822287		TSS200			0.25	5.93E-08
59	cg04182865	RNF14	5	141346431	N_Shelf	TSS200			−0.25	5.99E-08
60	cg14004161	SNX22	15	64442561	N_Shore	TSS1500			−0.25	6.84E-08
61	cg27553947	CLSTN1	1	9819767	N_Shelf	Body		rs76639688	−0.25	7.17E-08
62	cg05163268		5	180116385			rs11738824		0.25	7.20E-08
63	cg25416125	DGKA	12	56329615	Island	5′UTR			0.25	7.38E-08
64	cg21815704	GLRX2	1	193075249	S_Shore	TSS1500			−0.25	7.41E-08
65	cg07196571	SNX22	15	64442578	N_Shore	TSS1500			−0.25	7.46E-08
66	cg12007048	CTSD	11	1785701	S_Shore	TSS1500			−0.25	7.47E-08
67	cg01554529	FBXO6	1	11722935	N_Shore	TSS1500			−0.25	7.51E-08
68	cg27466532	RAPSN	11	47471400		TSS1500			−0.25	7.96E-08
69	cg16971827	CBL	11	119177430	N_Shelf	3′UTR			0.25	8.20E-08
70	cg18463607	EXOC1	4	56718320	N_Shore	TSS1500			0.25	8.54E-08
71	cg13879047	GRB10	7	50774217		5′UTR			−0.25	8.62E-08
72	cg19743406	LHFPL5	6	35771838	N_Shore	TSS1500		rs59697285	−0.25	8.66E-08
73	cg08930843	ZNF438	10	31182298		5′UTR			−0.25	8.76E-08
74	cg13451886	SLFN5	17	33568791	N_Shore	TSS1500			0.24	8.89E-08
75	cg21495704	TYROBP	19	36399346		TSS200		rs56006731	−0.24	9.02E-08
76	cg09674502	GFI1	1	92953279	S_Shore	TSS1500			−0.24	9.27E-08
77	cg27073431	CALM2	2	47404636	S_Shore	TSS1500			−0.24	9.33E-08
78	cg21159128	SSBP3	1	54693933		Body			−0.24	9.47E-08
79	cg04252203		3	194696866					−0.24	9.62E-08
80	cg13443575	SLFN13	17	33775961	N_Shore	TSS200			−0.24	9.72E-08
81	cg05001044	MIR1977	1	567312		TSS1500			−0.24	9.74E-08
82	cg13716760		9	15371248					−0.24	9.95E-08

These are CpGs with p values < 10^–7^ for blood DNA methylation analyses in 508 PEG1 individuals of European ancestry adjusting for age and gender

*Chr* Chromosome, *bp* base pair, *TSS* transcription start site, *TSS1500* within 1500 bps of a TSS, *TSS200* within 200 bps of a TSS, *UTR* untranslated region, *SNPs* listing dbSNP entries within a probe, *SNPs_10* listing dbSNP entries within 10 bp of the CpG site

We applied pathway enrichment analysis (based on DAVID) to two sets of genes that are located near two respective sets of PD-related CpGs: (1) 62 genes located near the 82 genome-wide significant CpGs; and (2) 1177 genes located near the 2000 most significant CpGs, respectively. Pathways and gene categories that are significant after adjusting for multiple comparisons can be found in Additional file 1: Table S2a. The set of 62 genes was significantly enriched with genes involved in primary immunodeficiency (Additional file 1: Table S2a). The set of 1177 genes was significantly enriched with genes involved in both the adaptive and innate immune response (the list of overlapping genes can be found in Additional file 1: Table S2a), such as leukocyte activation, cytokine production, antigen presentation, and also in actin cytoskeleton organization. Sensitivity analyses that excluded CpGs that co-located with SNPs led to equivalent results (data not shown).

Our meta-analysis across individuals of European and Hispanic ancestry revealed another highly significant CpG cg00175838 in a mitochondrial gene *DDAH2* (meta.P = 1.1 × 10^–9^) while maintaining high significance for both cg02489202 in *LARS2* (metaP = 4.7 × 10^–11^; Additional file 1: Table S3) and cg27553947 in *CLSTN1* (metaP = 9.9 × 10^–10^).

#### DNA methylation in blood with adjustment for blood cell composition

Our previous analysis identified several PD-related CpGs that are located near immune-related genes. Since DNA methylation levels differ greatly across blood cell types, it is possible that these CpGs track PD-related changes in cell composition. In a secondary analysis, we repeated our EWAS analysis using DNA methylation levels that were adjusted for blood cell count estimates. After adjustment, none of the CpGs remained significant at a genome-wide significance level in individuals of European ancestry (Additional file 1: Figure S1a). However, 19 CpGs remained significant at a suggestive significance level of *p* < 5.0 × 10^–6^ (Additional file 1: Table S4). These 19 CpGs tended to be located in CpG islands (53% vs an expected proportion of 31%; Additional file 1: Figure S1b) and not in gene bodies (11% vs 33%). The most significant CpG cg05001044 (*p* = 1.7 × 10^–7^) is hypomethylated in PD cases. It is located within 1500 bps of a transcription starting site for *MIR1977*, a site that was also implicated in our EWAS analysis that did not adjust for blood cell types (Table 1). Our gene/pathway enrichment analyses based on the 2000 most significant CpGs (in 1434 genes) implicated gene sets involved in neuron differentiation and the Wnt receptor signaling pathway (Additional file 1: Table S5a). Accounting for the direction of methylation changes did not change results. Our meta-analyses across individuals of European and Hispanic ancestry led to three CpGs with a suggestive association with PD (Additional file 1: Table S6): cg27191131 in *CEP63*, the intergenic cg13322234, and cg00175838 in *DDAH2* (metaP = 7.7 × 10^–6^).

#### DNA methylation in saliva

It is of interest whether methylation differs by PD status in other easily accessible sources of DNA. Thus, we examined DNA methylation levels in saliva-based analyses for a second set of individuals, since saliva is easier and cheaper to collect, store, and transport than blood and would facilitate future large population-based studies of methylation in PD.

In DNA-derived saliva samples from 259 individuals, five CpGs were significantly associated with PD status at the genome-wide level (Additional file 1: Figure S2a), after adjusting for age, sex, and race. Two significant CpGs which are hypomethylated in PD cases are located near H-ferritin genes (cg15133963 *p* = 1.1 × 10^–8^ and cg11748881 *p* = 7.2 × 10^–8^, Table 2). Gene/pathway enrichment analysis showed enrichment for gene sets related to neuron differentiation and projection (Additional file 1: Table S7a).

**Table 2:** List of the five PD-associated CpGs with *p* value < 10^–7^ for saliva-based DNA methylation analyses in 259 PEG2 individuals adjusting for age, gender, and race

					Relation to UCSC CpG island				Caucasian and Hispanic (N = 259)	
	CpG	Gene	Chr.	Position (bp)		Gene region	SNPs	SNPs_10	cor	*p* value
1	cg15133963	FTHL3	2	27616316	Island	Body			−0.35	1.05E-08
2	cg01820192	C21orf125	21	44869762		TSS200			−0.33	3.45E-08
3	cg22275276		6	33973531					−0.33	6.92E-08
4	cg11748881	FTH1	11	61734830	Island	1stExon		rs11554886	−0.33	7.15E-08
5	cg24742912	MYBPH	1	203146346		TSS1500	rs7538338		−0.32	9.10E-08

*Chr* Chromosome, *bp* base pair, *TSS* transcription start site, *TSS1500* within 1500 bps of a TSS, *TSS200* within 200 bps of a TSS, *SNPs* listing dbSNP entries within a probe, *SNPs_10* listing dbSNP entries within 10 bp of the CpG site

An overview for methylation results in blood and saliva, with and without adjustments, stratified by race, and the meta-analysis results are presented in Additional file 1: Table S8. While smoking affects DNA methylation levels, the association results with PD were largely unchanged after adjusting for smoking status. Also, empirical *p* values for DNA methylation and PD were similar to *p* values derived from t-test; therefore, empirical *p* values were not shown in the tables.

### Weighted correlation network analysis

#### DNA methylation in blood without adjustment for blood cell composition

**Fig. 3:**
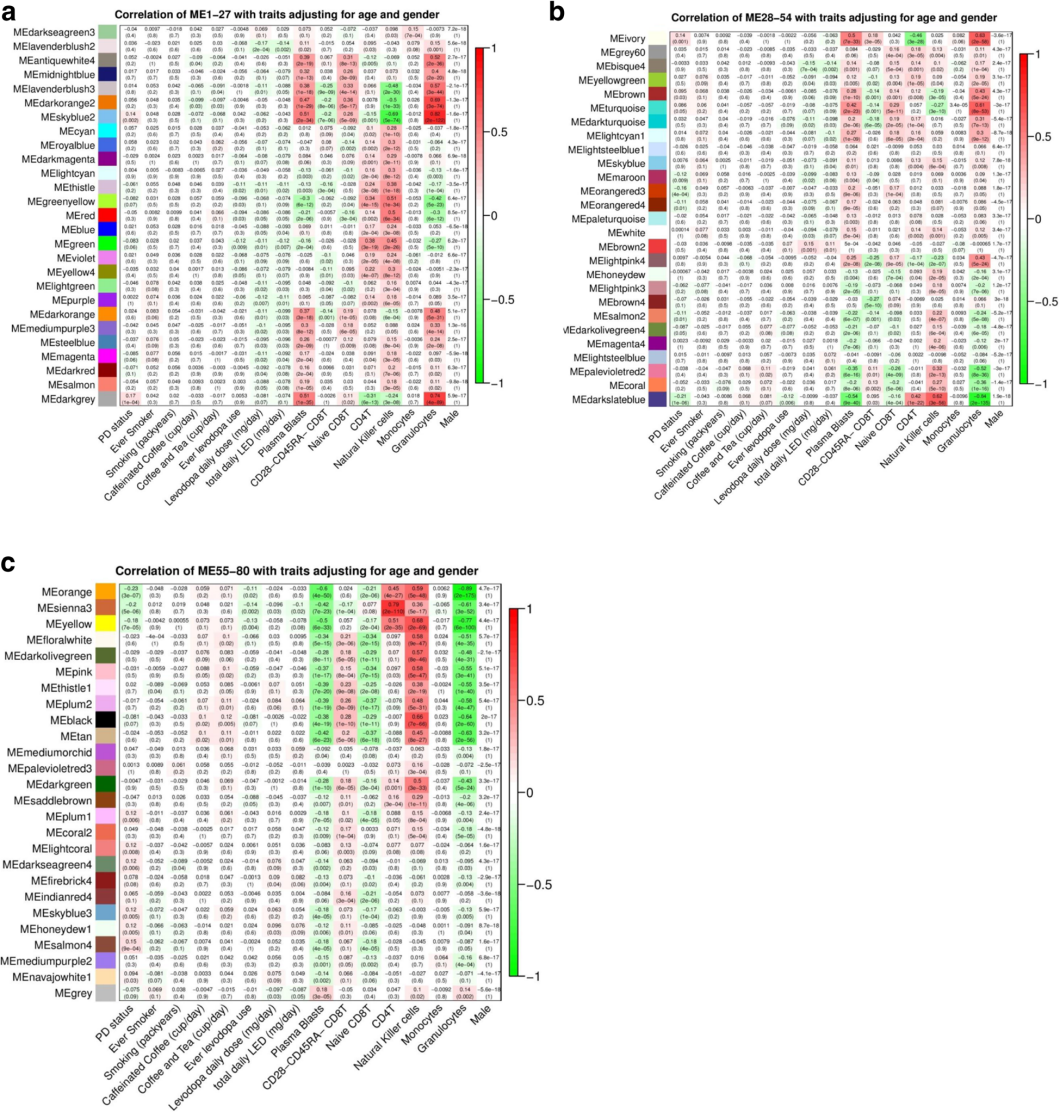
WGCNA results for PD and blood-based DNA methylation analyses without cell composition adjustment. Correlations of module eigengenes (ME) with PD status and other traits in 508 PEG1 individuals of European ancestry adjusting for age and gender. The rows represent ME and its color. The columns represent clinical traits. The Pearson’s correlation coefficients and the corresponding *p* values are shown for each cell. Red indicates positive correlations while color indicates negative correlations. **a** ME 1-27. **b** ME 28-54. **c** ME 55-80

Adjusting for age only, WGCNA clustered the 250,000 CpGs into 76 co-methylation modules (Additional file 1: Figure S3a–c). Of these, seven were significantly associated with PD at the Bonferroni threshold of *p* < 0.05/76 modules (approximately *p* < 5.0 × 10^–4^). These modules were also significantly associated with sex and blood cell composition, particularly granulocytes. We conducted additional WGCNA, adjusting for both age and sex. This approach generated 80 modules with six PD-associated modules (*p* < 5.0 × 10^–4^; Fig. 3a–c), also significantly associated with estimated blood cell counts but not associated with other factors, such as smoking, coffee/tea consumption, and levodopa medication use. Three of the six PD-associated modules were enriched for genes related to both the adaptive and the innate immune response (Additional file 1: Table S2b) and the rest showed enrichment in calcium ion binding, cell adhesion, and transcriptional activity. Moreover, two modules also showed enrichment of gene sets regulating actin cytoskeleton organization.

#### DNA methylation in blood with adjustment for blood cell composition

After adjusting for blood cell composition, we generated 97 modules with only one significant PD-associated module after Bonferroni correction and two modules with borderline significance (Additional file 1: Figure S4a–d). One module was enriched for genes related to calcium ion binding (Additional file 1: Table S5b), another for genes related to neuron differentiation and projection, whereas a third showed enrichment related to the Wnt receptor signaling pathways, especially through beta catenin.

Our systems biological analysis based on WGCNA highlighted epigenetic differences in multiple pathways of importance in PD etiology (Additional file 1: Figure S5).

We also conducted a WGCNA analysis without adjusting for age in order to identify modules with overlap for PD, age, and gender. We found one hyper-methylated module (i.e. thistle) that is significantly associated with PD status, increasing age, and male gender at the Bonferroni threshold of *p* < 5.0 × 10^–4^; Additional file 1: Figure S6a–c). The thistle module is enriched for developmental genes and related to neuronal differentiation processes, which further supports our previously published results of age acceleration in PD [5]. The correlations between each thistle module CpG and PD status, age, and being male can be found in Additional file 1: Table S9.

#### DNA methylation in saliva

Adjusting for age, sex, and race, we generated 48 modules with ten modules significantly associated with PD (*p* < 0.05/48 modules ~ 10^–3^; Additional file 1: Figure S7a, b) and also with blood cell types. Furthermore, these ten PD-associated modules were enriched for genes related to immune response (both adaptive and innate immunity), neuron projection, the Wnt signaling pathway, transcriptional activity, actin binding, and calcium-dependent cell–cell adhesion (Additional file 1: Table S7b). Additionally, two modules were borderline significant and enriched for genes related to the mitochondrial envelope membrane.

Methylation levels of the three most significant PD-associated CpGs in blood and saliva can be found in Additional file 1: Table S10 and results of the association between CpGs and PD status based on logistic regression are provided in Additional file 1: Table S11.

### Attempt to replicate results from previous methylation studies

We found one gene in Table 1, i.e. FOXK1, that positively regulates Wnt/beta-catenin signaling, for which there is overlap with the brain PD EWAS study of 5 PD and 6 control individuals that previously reported concordant methylation changes in brain and blood for 30 genes [22]. However, we failed to replicate the previously reported associations between PD and *FANCC* cg14115740 (*p* = 0.24) and *TNKS2* cg11963436 (*p* = 0.03) from an EWAS study of 45 participants that used whole blood DNA without adjustment for cell composition [4]. While previous targeted DNA methylation studies have implicated *SNCA*, our large-scale study did not provide evidence for epigenetic modifications in this gene in blood and saliva. We also failed to replicate associations between SNCA methylation and levodopa dosage reported previously [23]. The previous study reported methylation in opposite directions according to the patients’ sex (hyper vs hypo-methylation), yet sex had no influence on those experiments in cultured monocytes. The authors did not offer any explanation for the observed sex-dependent differences.

## Discussion

Our study is the first population-based study of PD that leverages a large sample size and the highly robust Illumina array platform. This rigorous and comprehensive study demonstrates that PD is associated with DNA methylation levels in blood and saliva. Many of our genome-wide significant CpGs and co-methylation modules correlate with changes in cell composition, consistent with our previous results surrounding highly significant associations between estimated blood cell counts and PD status [5]. It is unlikely that the genome-wide significant results represent false positives because this is arguably the largest and most comprehensive DNA methylation study of PD to date. This study stands out in terms of its careful matching of PD cases with suitable population-based controls, the identical handling of DNA samples, its analysis of two distinct sources of DNA (blood and saliva), its handling of potential confounders (e.g. blood counts, ethnicity, smoking), and the rigorous control of false positives resulting from multiple comparisons (using the Bonferroni correction and employing WGCNA). During the recruitment period, great attention was paid to “population” control selection using Medicare enrollee lists and residential tax assessor records in the three target counties. Also, every patient was examined by UCLA movement disorder specialists at least once and most multiple times over several years of follow-up and the diagnosis of iPD was re-affirmed. Another strength is the fact that we carefully verified ethnicity using 37 Ancestry Informative Markers (AIM) and we stratified the analysis by ethnicity to eliminate confusion. However, the information on coffee and tea consumption is self-reported, which is a study limitation. It is unlikely that PD status influenced the accuracy of reporting coffee/tea consumption; thus, we would expect non-differential misclassification that tends to bias estimates toward the null. Moreover, we have published a coffee EWAS study using the same samples [24], which was consistent with the literature on coffee and PD. For example, the estimated effect size for coffee consumption and PD risk for three additional cups of coffee per day (odds ratio [OR] 0.73, 95% confidence interval [CI] 0.58–0.92) in our study is consistent with the estimated effect size reported in a meta-analysis of 13 coffee and PD studies (OR 0.75, 95% CI 0.64–0.86) [25].

The fact that we could not reproduce many previous results from smaller studies raises serious concerns about the findings from previous studies. DNA methylation levels are subject to biases resulting from differences in DNA storage, blood tubes, bisulfite conversion, and batch effects. While the lack of replication of previous findings could reflect quality issues in our own data, we believe that it is unlikely because our large-scale DNA methylation data exhibited high quality according to a host of metrics including inter-array correlations, age prediction, sex prediction, and other quality metrics from the epigenetic clock software [5]. Further, our data lent themselves for reproducing published CpGs from other contexts, e.g. to study the effect of smoking (unreported findings). Yet, for the *SNCA* gene it is possible that the most relevant CpGs are not present on the Illumina 450 K array.

A novel aspect of our study is the utilization of WGCNA for genome-wide methylation data. This method greatly reduced the multiple testing problem: instead of relating 486,000 CpGs to PD status, we only considered approximately 100 modules. The functional enrichment of modules was more significant than that of our EWAS analysis which highlights that WGCNA is a biologically meaningful data reduction method. WGCNA not only strengthened our findings from the EWAS analysis, but also implicated interesting pathways in the context of PD such as cytoskeleton organization.

Caution must be exercised when it comes to interpreting individual CpGs (or sets of CpGs) in terms of neighboring genes because DNA methylation levels exhibit only weak associations with neighboring gene expression levels across individuals [16]. With all due caution, we discuss our gene enrichment analysis results below.

### Mitochondrial dysfunction

Mitochondrial dysfunction has long been considered important for PD etiology [26]. Landmark studies reported loss of dopaminergic neurons from mitochondrial complex I respiratory chain deficiency in the substantia nigra [27]. We identified three CpGs located in mitochondria-related genes (*LARS2*, *MIR1977*, and *DDAH2*) that were significantly associated with PD status in blood-based analyses. While blood methylation patterns differed from those in saliva, it is noteworthy that two PD-related co-methylation modules in saliva were significantly enriched with mitochondrial inner membrane genes. The *LARS2*-encoded protein (mitochondrial Leucyl-tRNA Synthetase 2), which catalyzes the aminoacylation of mtRNA^Leu^, has been related to gene expression and nucleotide binding. Interestingly, a small postmortem study analyzing gene expression in dopaminergic neurons from the substantia nigra region comparing iPD patients and controls found hypo-expression of LARS2 among patients [28]. *MIR1977* encodes a microRNA with sequences that map to the mitochondrial genome; a role for mitochondrial DNA gene regulation has been suggested. *DDAH2* plays a role in regulating nitric oxide (NO) generation [29]. Excessive amounts of NO are neurotoxic and impair the function of the mitochondrial respiratory chain [30]. *DDAH2* regulates levels of ADMA which inhibits nitric oxide synthase (NOS) activity [31]. We recently reported that variants in the gene *NOS1* encoding an NO-producing enzyme modifies PD risk in organophosphate pesticide exposed individuals [32] and in a pesticide-induced mouse model of PD the administration of a NOS inhibitor mitigated neuronal death [33].

### Cytoskeleton function

Dopamine transport involves microtubules and actin as key components of the axonal cytoskeleton. Our meta-analysis of blood-based DNA adjusted for blood cell composition suggested *CEP63* cg27191131 as a candidate for PD. CEP63 acts in the centrosome, the major microtubule organizing center of cells. Notably, in PD, parkin, a protein-ubiquitin E3 ligase responsible for some types of familial PD, stabilizes microtubules, protects neurons, and also acts in the centrosome assisting in ubiquitination and degradation of misfolded proteins [34, 35]. Our saliva-based gene set enrichment analysis further supported this finding with enrichment of genes related to the microtubule organizing center among the hypermethylated CpGs. Additionally, our EWAS and WGCNA in blood without adjustment for blood cell composition found PD-associated genes and modules significantly enriched for actin cytoskeleton organization as did a module derived from saliva. Actin is responsible for cell movement in the nervous system including axon and synapse formation [36] and, in fact, synaptic dysfunction resulting from vesicular transport disruption is a hallmark of PD [37].

### Systemic immune response

Reducing neuroinflammation may be important to prevent PD [38]. Studies have recorded higher levels of inflammatory cytokines released by microglia in the substantia nigra of PD patients [39]. In our blood-based DNA EWAS without adjusting for cell compositions, we identified CpGs in three immune-related genes (*ABCB9*, *C1orf200*, *AZU1*) as significantly associated with PD. The enrichment analysis strongly supported this pathway. Further, WGCNA identified three PD-associated modules enriched for immune response in blood-based methylation) and one immune-associated module from saliva-based methylation.

We recently reported that the DNA methylation age of the immune system is significantly older in PD patients in the same samples and blood cell composition of PD patients differed from controls [5]. PD patients’ peripheral blood contained more granulocytes but fewer T helper cells and B cells. These findings were replicated using WGCNA. Every PD-associated module was also significantly associated with blood cell types, most notably granulocyte count, supporting the notion that systemic differences in immune response may play a role in PD pathology and removing the contributions of blood cell composition prior to methylation analyses may result in over-adjustment. The fact that none of the probes are significantly associated with PD after correcting for blood cell counts might also reflect that our Bonferroni corrected threshold of 1 × 10^–7^ is too stringent given that many CpGs are highly correlated. Note that the strong correlation pattern between CpGs gives rise to large modules found by WGCNA. However, the 24 CpGs inside our most significant gene *LARS2* (Table 1) exhibit relatively low pairwise correlations even after adjusting for blood cell counts (Additional file 1: Table S12).

### The Wnt receptor signaling pathway

The Wnt receptor signaling pathway has been suggested as a therapeutic target for PD [40]. Both the “classic” (through β-catenin) and “non-classical” pathways play roles in dopaminergic cell development and synaptic function. PD-associated proteins encoded by *PARK2* (protein: parkin) and *LRRK2* have been shown to modify classic Wnt signaling [41]. While our study did not find evidence for epigenetic modifications in these PD genes, we identified other genes (*APC* and *AXIN1*) involved in classic Wnt signaling in enrichment analysis. Interestingly, decreased Wnt signaling has also been reported in AD [42]. *CALM2*, one of the AD-related genes we associated in blood-based methylation analysis with PD status, has a function in calcium ion binding which is important in the non-classical pathway [43].

### Brain iron and ferritin

Lastly, in our saliva-based DNA methylation EWAS, two out of five significant PD-associated CpGs were located in the H-ferritin genes. The H-chain of the iron-storage protein is responsible for iron uptake and iron oxidation in the brain. Iron overload which leads to oxidative stress, mitochondrial dysfunction, and alpha-synuclein aggregation may play a role in PD pathogenesis [44]. In PD patients, higher iron levels have been found in the substantia nigra [45] and proliferation of ferritin-positive microglia may be involved in dopaminergic neuron death [46].

Given that all analyses are conducted in blood and saliva where neuronal differentiation cannot be assessed, there is a need to replicate these findings for the Wnt signaling pathway.

## Conclusions

Our study provides the first evidence for epigenetic differences related to five biological pathways in PD (Additional file 1: Figure S5). Furthermore, our findings suggest that adjusting for blood cell counts in blood DNA methylation studies may “throw out the baby with the bathwater” when it comes to detecting immune system related pathways in PD.

## Additional file

Additional file 1: Supplementary tables and figures that show the following: characteristics of study participants, results of EWAS, WGCNA, meta-analyses, and gene set enrichment analyses. (DOCX 8897 kb)

## Abbreviations

AD: Alzheimer’s disease
AIM: Ancestry Informative Markers
DAVID: Database for Annotation, Visualization and Integrated Discovery
EWAS: Epigenome-wide association analysis
ME: Module eigengene
NO: Nitric oxide
PD: Parkinson’s disease
PEG: Parkinson’s environment and gene study
WGCNA: Weighted correlation network analysis
