# Supplementary material for: Parkinson’s disease is associated with DNA methylation levels in human blood and saliva
Source: Genome Med. 2017 Aug 30;9:76. doi: 10.1186/s13073-017-0466-5 (PMC5576382; doi:10.1186/s13073-017-0466-5)

## Additional files 1. Supplementary tables and figures (all DOCX)

**Table S1. Characteristics of the 572 PEG1 and 259 PEG2 subjects.**

**Table S2. Gene set enrichment analysis for PD-associated CpGs in blood without blood cell composition adjustment.** (a) Gene set enrichment analysis for the top 82 (p < 10^-7^) and top 2,000 PD-associated CpGs in blood DNA methylation levels from 508 PEG1 subjects of European ancestry adjusting for age and gender and stratified by the direction of methylation changes. (b) Gene set enrichment analysis for CpGs in the 6 PD-associated modules in blood DNA methylation levels from 508 PEG1 subjects of European ancestry adjusting for age and gender (module p-value cutoff = 5x10^-4^)

**Table S3. Meta-analysis of blood EWAS results in European and Hispanic ancestry without blood cell composition adjustment.** List of the 116 PD-associated CpGs with meta.p-value < 10^-7^ in PEG1 subjects of 508 European and 64 Hispanic ancestry adjusting for age and gender.

**Table S4. List of the 19 PD-associated CpGs in blood based methylation analyses with cell composition adjustment.** These are CpGs with p-value < 5x10^-6^ in blood based DNA methylation analyses for 508 PEG1 subjects of European ancestry adjusting for age, gender, and cell composition.

**Table S5. Gene set enrichment analysis for PD-associated CpGs in blood with cell composition adjustment.** (a) Gene set enrichment analysis for the top 2,000 PD-associated CpGs in blood based DNA methylation analyses for 508 PEG1 subjects of European ancestry adjusting for age, gender and cell composition and stratified by direction of methylation changes. (b) Gene set enrichment analysis for CpGs in the 3 PD-associated modules in blood based DNA methylation analyses for 508 PEG1 subjects of European ancestry adjusting for age, gender and cell composition (module p-value cutoff = 5x10^-4^)

**Table S6. Meta-analysis of blood based EWAS results in subjects with European and Hispanic ancestry with cell composition adjustment.** List of the 2 PD-associated CpGs with meta.p-value < 5x10^-6^ in PEG1 for 508 European ancestry and 64 Hispanic ancestry subjects adjusting for age, gender and cell composition.

**Table S7. Gene set enrichment analysis for PD-associated CpGs in saliva.** (a) Gene set enrichment analysis for the top 2,000 PD-associated CpGs in saliva based DNA methylation analyses for 259 PEG2 subjects adjusting for age, gender, and race and stratified by the direction of methylation changes. (b) Gene set enrichment analysis for CpGs in the 10 PD-associated modules in saliva based DNA methylation analyses for 259 PEG2 subjects adjusting for age, gender, and race (module p-value cutoff = 1x10^-3^)

**Table S8. Summary of EWAS results in blood and saliva.** Correlations between PD and DNA methylation levels at 450k CpG sites in blood and saliva.

**Table S9. Correlations between each thistle module CpG and PD status, age, and male, respectively.** The Pearson’s correlation coefficients and the corresponding p-values are shown.

**Table S10. Methylation levels of the 3 most significant PD-associated CpGs in blood and saliva.** The median, min, max methylation levels of the 3 most significant PD-associated CpGs in blood and saliva.

**Table S11. Logistic regression results of the association between CpGs and PD status.** (a) 82 blood based top hits (p-value < 10^-7^) without adjustment for blood cell compositions from **Table S12. Correlations between 24 CpGs on the LARS2 gene.** The Pearson’s correlation coefficients and the corresponding p-values are shown for each cell. Red color indicates positive correlations while green color indicates negative correlations. (a) Using the DNA methylation data in blood that were adjusted for age and sex. (b) Using the DNA methylation data in blood that were adjusted for age, sex, and cell composition.

**Table 1**. (b) 19 blood based top hits (p-value < 5x10^-6^) with adjustment for blood cell compositions from **Additional file 1: Table S4**. (c) 5 saliva based top hits (p-value < 10^-7^) from **Table 2**.

**Figure S1. EWAS results for PD and blood based DNA methylation with cell composition adjustment.** Differential methylation associated with PD status in 508 PEG1 subjects of European ancestry adjusting for age, gender, and blood cell composition. (a) Manhattan plot of p-values adjusted for age and gender (red line: p-value threshold of 10^-7^). (b) Distributions of CpGs relative to CpG island and gene regions for all 450k CpGs on the microarray and the 19 most significant coffee-associated CpGs listed in **Table S5**. (c) Distribution of DNA methylation levels for the top 3 most significant PD-associated CpGs by PD status (1=PD).

**Figure S2.** **EWAS results for PD and saliva based DNA methylation.** Differential methylation associated with PD status in 259 PEG2 subjects adjusting for age, gender and race. (a) Manhattan plot of p-values adjusted for age and gender (red line: p-value threshold of 10^-7^). (b) Distributions of CpGs relative to CpG island and gene regions for all 450k CpGs on the microarray and the 5 most significant coffee-associated CpGs listed in **Table 2**. (c) Distribution of DNA methylation levels in the top 3 most significant PD-associated CpGs by PD status (1=PD).

**Figure S3. WGCNA results for PD and blood based DNA methylation adjusting for age.** Correlations of module eigengenes (ME) with PD status and other traits in 508 PEG1 subjects of European ancestry adjusting for age. The rows represent ME and its color. The columns represent clinical traits. The Pearson’s correlation coefficients and the corresponding p-values are shown for each cell. Red color indicates positive correlations while green color indicates negative correlations.

**Figure S4. WGCNA results for PD and blood based DNA methylation with cell composition adjustment.** Correlations of module eigengenes (ME) with PD status and other traits in 508 PEG1 subjects of European ancestry adjusting for age, gender, and cell composition. The rows represent ME and its color. The columns represent clinical traits. The Pearson’s correlation coefficients and the corresponding p-values are shown for each cell. Red color indicates positive correlations while green color indicates negative correlations.

**Figure S5. A summary of biological pathways identified in this study.** These biological pathways are involved in PD pathogenesis or cell dysfunction.

**Figure S6. WGCNA results for PD and blood based DNA methylation.** Correlations of module eigengenes (ME) with PD status and other traits in 508 PEG1 subjects of European ancestry. The rows represent ME and its color. The columns represent clinical traits. The Pearson’s correlation coefficients and the corresponding p-values are shown for each cell. Red color indicates positive correlations while green color indicates negative correlations.

**Figure S7. WGCNA results for PD and saliva based DNA methylation.** Correlations of module eigengenes (ME) with PD status and other traits in 259 PEG2 subjects adjusting for age, gender, and race. The rows represent ME and its color. The columns represent clinical traits. The Pearson’s correlation coefficients and the corresponding p-values are shown for each cell. Red color indicates positive correlations while green color indicates negative correlations.

| **Table S1. Characteristics of the 572 PEG1 and 259 PEG2 subjects.** | | |  |  |  |
| --- | --- | --- | --- | --- | --- |
|  | **PEG1 blood samples (N=572)** | |  | **PEG2 saliva samples (N=259)** | |
|  | **European ancestry subjects (N=508)** | **Hispanic ancestry subjects (N=64)** |  | **European ancestry subjects (N=166)** | **Hispanic ancestry subjects (N=93)** |
| PD status, N (%) | 289 (57%) | 46 (72%) |  | 82 (49%) | 46 (49%) |
| Age at blood draw, Mean (range) | 70 (35-92) | 65 (35-86) |  | 69 (40-88) | 68 (36-88) |
| Male, N (%) | 282 (55%) | 41 (64%) |  | 90 (54%) | 56 (60%) |
| Ever cigarette smoker, N (%) | 266 (52%) | 34 (53%) |  | 76 (46%) | 40 (43%) |
| Smoking (pack-years), Mean (SD) | 12.9 (22.5) | 6.0 (12.9) |  | 6.8 (13.0) | 5.0 (12.4) |
| Caffeinated coffee consumption (cup/day), Mean (SD) | 1.7 (2.3) | 1.4 (2.8) |  | 1.3 (1.6) | 1.1 (1.5) |
| Coffee and Tea consumption (cup/day), Mean (SD) | 3.1 (2.9) | 2.4 (3.1) |  | 2.0 (2.5) | 1.5 (1.8) |
| Ever levodopa use in PD cases, N (%) | 203 (70%) | 31 (48%) |  | - | - |
| Levodopa daily dose (mg/day) in PD cases, Mean (SD) | 284.5 (269.1) | 303.5 (268.2) |  | - | - |
| Total daily levodopa equivalent dose (LED) in PD cases (mg/day), Mean (SD) | 349.9 (282.7) | 369.2 (278.8) |  | - | - |
| PEG: Parkinson's Environment and Gene study; PD: Parkinson's Disease. *Missing information (PEG1 European ancestry/PEG1 Hispanic ancestry/PEG2 European ancestry/PEG2 Hispanic ancestry): pack-year of smoking (N=0/0/1/0), caffeinated coffee (N=12/1/0/0), coffee and tea (N=25/2/7/3). | | | | | |

| **Table S2. Gene set enrichment analysis for PD-associated CpGs in blood without blood cell composition adjustment.**  (a) Gene set enrichment analysis for the top 82 (p < 10^-7^) and top 2,000 PD-associated CpGs in blood DNA methylation levels from 508 PEG1 subjects of European ancestry adjusting for age and gender and stratified by the direction of methylation changes. | | | | | | | | | | | | | | | | | | | | | | |
| --- | --- | --- | --- | --- | --- | --- | --- | --- | --- | --- | --- | --- | --- | --- | --- | --- | --- | --- | --- | --- | --- | --- |
| **Rank** | | | | | **Category** | | | | | | **Term** | | **p-value** | | **Bonferroni** | | **Benjamini** | | **FDR** | | **Overlap Genes (N)** | **Fold Enrichment** |
| ***All CpGs (82 PD associated CpGs with p-value < 10^-7^ in 62 genes)*** | | | | | | | | | | | | |  | |  | |  | |  | |  |  |
| 1 | | | | KEGG_PATHWAY | | | | | | | hsa05340:Primary immunodeficiency | | 1.06E-02 | | 4.62E-01 | | 4.62E-01 | | 1.01E+01 | | 3 | 18.16 |
| 2 | | | | GOTERM_BP_FAT | | | | | | | GO:0030518~steroid hormone receptor signaling pathway | | 1.80E-02 | | 1.00E+00 | | 9.98E-01 | | 2.40E+01 | | 3 | 14.28 |
| 3 | | | | GOTERM_BP_FAT | | | | | | | GO:0009725~response to hormone stimulus | | 4.07E-02 | | 1.00E+00 | | 9.75E-01 | | 4.66E+01 | | 5 | 3.76 |
| 4 | | | | GOTERM_MF_FAT | | | | | | | GO:0005524~ATP binding | | 4.09E-02 | | 1.00E+00 | | 1.00E+00 | | 4.05E+01 | | 11 | 1.97 |
| 5 | | | | KEGG_PATHWAY | | | | | | | hsa04070:Phosphatidylinositol signaling system | | 4.34E-02 | | 9.24E-01 | | 7.24E-01 | | 3.57E+01 | | 3 | 8.59 |
| ***All CpGs (2,000 CpGs in 1,177 genes)*** | | | | | | | | | | | | |  | |  | |  | |  | |  |  |
| 1 | | | | GOTERM_BP_FAT | | | | | | GO:0045321~leukocyte activation | | | 5.46E-08 | | 1.77E-04 | | 1.77E-04 | | 9.92E-05 | | 40 | 2.62 |
| 2 | | | | GOTERM_BP_FAT | | | | | | GO:0006955~immune response | | | 6.98E-08 | | 2.26E-04 | | 1.13E-04 | | 1.27E-04 | | 81* | 1.86 |
| 3 | | | | GOTERM_BP_FAT | | | | | | GO:0030098~lymphocyte differentiation | | | 5.64E-06 | | 1.81E-02 | | 3.04E-03 | | 1.02E-02 | | 21 | 3.23 |
| 4 | | | | GOTERM_BP_FAT | | | | | | GO:0006952~defense response | | | 4.90E-05 | | 1.47E-01 | | 1.21E-02 | | 8.90E-02 | | 65 | 1.67 |
| 5 | | | | GOTERM_BP_FAT | | | | | | GO:0001816~cytokine production | | | 1.03E-04 | | 2.84E-01 | | 1.94E-02 | | 1.87E-01 | | 12 | 4.13 |
| 6 | | | | GOTERM_BP_FAT | | | | | | GO:0019882~antigen processing and presentation | | | 1.91E-04 | | 4.61E-01 | | 3.20E-02 | | 3.46E-01 | | 16 | 3.05 |
| 7 | | | | KEGG_PATHWAY | | | | | | hsa04660:T cell receptor signaling pathway | | | 2.00E-04 | | 3.19E-02 | | 3.19E-02 | | 2.42E-01 | | 20 | 2.57 |
| 8 | | | | GOTERM_BP_FAT | | | | | | GO:0030036~actin cytoskeleton organization | | | 2.09E-04 | | 4.92E-01 | | 3.33E-02 | | 3.79E-01 | | 30 | 2.10 |
| 9 | | | | GOTERM_BP_FAT | | | | | | GO:0006954~inflammatory response | | | 3.49E-04 | | 6.78E-01 | | 4.61E-02 | | 6.33E-01 | | 38 | 1.85 |
| 10 | | | | KEGG_PATHWAY | | | | | | hsa04940:Type I diabetes mellitus | | | 5.90E-04 | | 9.12E-02 | | 3.14E-02 | | 7.12E-01 | | 11 | 3.64 |
| ***Hypomethylated-CpGs (1,598 CpGs in 963 genes)*** | | | | | | | | | | | | |  | |  | |  | |  | |  |  |
| 1 | | | | GOTERM_BP_FAT | | | | | | GO:0045646~regulation of erythrocyte differentiation | | | 1.66E-05 | | 4.74E-02 | | 4.74E-02 | | 2.98E-02 | | 8 | 8.73 |
| 2 | | | | GOTERM_BP_FAT | | | | | | GO:0006952~defense response | | | 5.90E-05 | | 1.58E-01 | | 5.59E-02 | | 1.06E-01 | | 55 | 1.76 |
| 3 | | | | GOTERM_BP_FAT | | | | | | GO:0045637~regulation of myeloid cell differentiation | | | 1.91E-04 | | 4.29E-01 | | 1.31E-01 | | 3.43E-01 | | 13 | 3.65 |
| 4 | | | | GOTERM_BP_FAT | | | | | | GO:0006954~inflammatory response | | | 2.67E-04 | | 5.43E-01 | | 1.45E-01 | | 4.79E-01 | | 33 | 1.99 |
| 5 | | | | GOTERM_MF_FAT | | | | | | GO:0008092~cytoskeletal protein binding | | | 3.13E-04 | | 2.58E-01 | | 2.58E-01 | | 4.90E-01 | | 46 | 1.74 |
| 6 | | | | GOTERM_BP_FAT | | | | | | GO:0006955~immune response | | | 3.41E-04 | | 6.31E-01 | | 1.53E-01 | | 6.11E-01 | | 57 | 1.62 |
| 7 | | | | GOTERM_CC_FAT | | | | | | GO:0005764~lysosome | | | 4.06E-04 | | 1.67E-01 | | 1.67E-01 | | 5.74E-01 | | 24 | 2.25 |
| 8 | | | | GOTERM_BP_FAT | | | | | | GO:0001816~cytokine production | | | 4.37E-04 | | 7.22E-01 | | 1.67E-01 | | 7.82E-01 | | 10 | 4.27 |
| 9 | | | | GOTERM_MF_FAT | | | | | | GO:0003779~actin binding | | | 4.63E-04 | | 3.56E-01 | | 1.98E-01 | | 7.25E-01 | | 33 | 1.93 |
| ***Hypermethylated-CpGs (402 CpGs in 244 genes)*** | | | | | | | | | | | | |  | |  | |  | |  | |  |  |
| 1 | | | | GOTERM_BP_FAT | | | | | | GO:0046649~lymphocyte activation | | | 1.48E-08 | | 2.42E-05 | | 2.42E-05 | | 2.49E-05 | | 17 | 6.18 |
| 2 | | | | GOTERM_BP_FAT | | | | | | GO:0045321~leukocyte activation | | | 3.86E-08 | | 6.29E-05 | | 2.10E-05 | | 6.48E-05 | | 18 | 5.38 |
| 3 | | | | KEGG_PATHWAY | | | | | | hsa04660:T cell receptor signaling pathway | | | 8.31E-07 | | 8.64E-05 | | 8.64E-05 | | 9.29E-04 | | 12 | 6.89 |
| 4 | | | | KEGG_PATHWAY | | | | | | hsa04940:Type I diabetes mellitus | | | 3.45E-06 | | 3.58E-04 | | 1.79E-04 | | 3.85E-03 | | 8 | 11.81 |
| 5 | | | | GOTERM_MF_FAT | | | | | | GO:0005524~ATP binding | | | 2.28E-05 | | 8.31E-03 | | 4.16E-03 | | 3.14E-02 | | 39 | 2.03 |
| 6 | | | | GOTERM_BP_FAT | | | | | | GO:0006955~immune response | | | 2.59E-05 | | 4.14E-02 | | 3.52E-03 | | 4.35E-02 | | 25 | 2.62 |
| 7 | | | | KEGG_PATHWAY | | | | | | hsa05332:Graft-versus-host disease | | | 2.91E-05 | | 3.02E-03 | | 7.56E-04 | | 3.25E-02 | | 7 | 11.13 |
| 8 | | | | KEGG_PATHWAY | | | | | | hsa04650:Natural killer cell mediated cytotoxicity | | | 2.31E-04 | | 2.38E-02 | | 4.80E-03 | | 2.58E-01 | | 10 | 4.66 |
| 9 | | | | GOTERM_BP_FAT | | | | | | GO:0006915~apoptosis | | | 2.35E-04 | | 3.19E-01 | | 2.11E-02 | | 3.94E-01 | | 21 | 2.52 |
| GOTERM_BP: Biological Process, GOTERM_MF: Molecular Function, GOTERM_CC: Cellular Component, KEGG: Kyoto Encyclopedia of Genes and Genomes.  *Genes: LY86, PPARG, IL19, FASLG, TNFSF13, LY9, HLA-DMB, PDCD1, MBP, TMEM173, IL1RAP, IFNG, CEACAM8, LTF, NFIL3, LOC285830, RAB27A, F12, POU2AF1, SIT1, LYN, NCF2, IL27, NCF4, TP53, HLA-A, HLA-E, HLA-F, IL18BP, LAX1, LYST, CD300LF, IL12B, TREM2, CLEC5A, CTSG, CSF3, ITGAL, IL1R1, LST1, TNF, MAP4K2, HFE, FCGRT, SLC11A1, TUBB, TNFRSF1B, IL17B, PVRL1, FCN2, FCGR1B, TAP1, CNR2, FCER1G, HLA-DOA, CD28, IL2RA, CD1C, MARCH8, FOXP1, PSMB8, CD1D, PSMB9, LAT, CORO1A, NOTCH1, SARM1, ETS1, OTUB1, IKBKG, PLCG2, RFX1, AIRE…etc. | | | | | | | | | | | | | | | | | | | | | | |
| (b) Gene set enrichment analysis for CpGs in the 6 PD-associated modules in blood DNA methylation levels from 508 PEG1 subjects of European ancestry adjusting for age and gender (module p-value cutoff = 5x10^-4^) | | | | | | | | | | | | | | | | | | | | | |  |
| **Rank** | | | | | **Category** | **Term** | | | | | **p-value** | | **Bonferroni** | | **Benjamini** | | **FDR** | | **Overlap Genes (N)** | | **Fold Enrichment** |  |
| ***Hypomethylated-module*** | | | | | |  | | | | |  | |  | |  | |  | |  | |  |  |
|  | | ***Orangered3 module (Cor=-0.16, p-value=4x10^-4^; 56 CpGs in 42 genes)*** | | | | | | | | | | | | | | | | | | | |  |
| 1 | | GOTERM_BP_FAT | | | | | GO:0031399~regulation of protein modification process | | | | 3.19E-02 | | 1.00E+00 | | 1.00E+00 | | 3.61E+01 | | 4 | | 5.56 |  |
| 2 | | GOTERM_MF_FAT | | | | | GO:0030528~transcription regulator activity | | | | 4.48E-02 | | 9.95E-01 | | 9.28E-01 | | 4.07E+01 | | 8 | | 2.29 |  |
|  | | ***Darkslateblue module (Cor=-0.21, p-value=1x10^-6^; 819 CpGs in 468 genes)*** | | | | | | | | | | | | | | | | | | | |  |
| 1 | | GOTERM_MF_FAT | | | | | GO:0043565~sequence-specific DNA binding | | | | 1.90E-05 | | 1.07E-02 | | 2.69E-03 | | 2.78E-02 | | 35 | | 1.55 |  |
| 2 | | KEGG_PATHWAY | | | | | hsa04070:Phosphatidylinositol signaling system | | | | 1.99E-04 | | 2.34E-02 | | 2.34E-02 | | 2.28E-01 | | 10 | | 1.74 |  |
| 3 | | GOTERM_MF_FAT | | | | | GO:0003700~transcription factor activity | | | | 3.95E-04 | | 2.00E-01 | | 2.76E-02 | | 5.77E-01 | | 44 | | 1.53 |  |
|  | | ***Orange module (Cor=-0.23, p-value=3x10^-7^; 1,681 CpGs in 888 genes)*** | | | | | | | | | | | | | | | | | | | |  |
| 1 | | GOTERM_MF_FAT | | | | | GO:0005509~calcium ion binding | | | | 1.16E-04 | | 9.39E-02 | | 4.81E-02 | | 1.80E-01 | | 70 | | 1.59 |  |
| 2 | | GOTERM_BP_FAT | | | | | GO:0007155~cell adhesion | | | | 1.68E-04 | | 3.68E-01 | | 1.08E-01 | | 2.99E-01 | | 54 | | 1.70 |  |
|  | | ***Sienna3 module (Cor=-0.20, p-value=5x10^-6^; 1,695 CpGs in 939 genes)*** | | | | | | | | | | | | | | | | | | | |  |
| 1 | | GOTERM_BP_FAT | | | | | GO:0006955~immune response | | | | 5.03E-09 | | 1.47E-05 | | 1.47E-05 | | 9.03E-06 | | 70 | | 2.10 |  |
| 2 | | GOTERM_BP_FAT | | | | | GO:0045321~leukocyte activation | | | | 1.99E-07 | | 5.81E-04 | | 1.94E-04 | | 3.58E-04 | | 33 | | 2.83 |  |
| 3 | | GOTERM_BP_FAT | | | | | GO:0046649~lymphocyte activation | | | | 3.22E-07 | | 9.38E-04 | | 2.35E-04 | | 5.77E-04 | | 29 | | 3.02 |  |
| 4 | | GOTERM_BP_FAT | | | | | GO:0006954~inflammatory response | | | | 2.72E-06 | | 7.90E-03 | | 1.32E-03 | | 4.88E-03 | | 37 | | 2.36 |  |
| 5 | | GOTERM_BP_FAT | | | | | GO:0042981~regulation of apoptosis | | | | 1.37E-05 | | 3.91E-02 | | 4.97E-03 | | 2.45E-02 | | 67 | | 1.73 |  |
| 6 | | GOTERM_BP_FAT | | | | | GO:0002449~lymphocyte mediated immunity | | | | 1.16E-04 | | 2.86E-01 | | 1.67E-02 | | 2.07E-01 | | 13 | | 3.85 |  |
|  | ***Yellow module (Cor=-0.18, p-value=7x10^-5^; 3000 CpGs in 1,617 genes; 15,118 CpGs in total)*** | | | | | | | | | | | | | | | | | | | | |  |
| 1 | GOTERM_BP_FAT | | | | | | | GO:0009611~response to wounding | | | 1.09E-05 | | 3.86E-02 | | 4.90E-03 | | 2.00E-02 | | 76 | | 1.66 |  |
| 2 | GOTERM_BP_FAT | | | | | | | GO:0006954~inflammatory response | | | 3.76E-05 | | 1.27E-01 | | 1.04E-02 | | 6.90E-02 | | 51 | | 1.82 |  |
| 3 | GOTERM_BP_FAT | | | | | | | GO:0006915~apoptosis | | | 1.53E-04 | | 4.24E-01 | | 2.48E-02 | | 2.80E-01 | | 79 | | 1.52 |  |
| 4 | GOTERM_BP_FAT | | | | | | | GO:0016477~cell migration | | | 1.98E-04 | | 5.11E-01 | | 2.71E-02 | | 3.63E-01 | | 43 | | 1.81 |  |
| 5 | GOTERM_MF_FAT | | | | | | | GO:0008092~cytoskeletal protein binding | | | 3.92E-04 | | 3.75E-01 | | 3.30E-02 | | 6.32E-01 | | 67 | | 1.54 |  |
| 6 | GOTERM_MF_FAT | | | | | | | GO:0003779~actin binding | | | 6.04E-04 | | 5.15E-01 | | 3.94E-02 | | 9.71E-01 | | 47 | | 1.67 |  |
| ***Hypermethylated-modules*** | | | | | | | |  | | |  | |  | |  | |  | |  | |  |  |
|  | ***Darkgrey module (Cor=0.17, p-value=1x10^-4^; 2,555 CpGs in 1310 genes)*** | | | | | | | | | | | | | | | | | | | | |  |
| 1 | GOTERM_BP_FAT | | | | | | | GO:0045321~leukocyte activation | | | 1.34E-20 | | 4.67E-17 | | 4.67E-17 | | 2.46E-17 | | 64 | | 3.77 |  |
| 2 | GOTERM_BP_FAT | | | | | | | GO:0046649~lymphocyte activation | | | 3.65E-20 | | 1.27E-16 | | 6.35E-17 | | 6.68E-17 | | 57 | | 4.08 |  |
| 3 | KEGG_PATHWAY | | | | | | | hsa04660:T cell receptor signaling pathway | | | 3.13E-16 | | 5.43E-14 | | 5.43E-14 | | 4.00E-13 | | 38 | | 4.68 |  |
| 4 | GOTERM_BP_FAT | | | | | | | GO:0006955~immune response | | | 1.32E-12 | | 4.60E-09 | | 4.60E-10 | | 2.42E-09 | | 101 | | 2.09 |  |
| 5 | GOTERM_BP_FAT | | | | | | | GO:0006915~apoptosis | | | 1.35E-12 | | 4.69E-09 | | 4.27E-10 | | 2.47E-09 | | 92 | | 2.18 |  |
| 6 | KEGG_PATHWAY | | | | | | | hsa04650:Natural killer cell mediated cytotoxicity | | | 1.56E-11 | | 2.55E-09 | | 1.27E-09 | | 1.89E-08 | | 36 | | 3.60 |  |
| 7 | GOTERM_BP_FAT | | | | | | | GO:0030036~actin cytoskeleton organization | | | 4.61E-09 | | 1.60E-05 | | 5.73E-07 | | 8.44E-06 | | 43 | | 2.71 |  |
| 8 | GOTERM_BP_FAT | | | | | | | GO:0033077~T cell differentiation in the thymus | | | 8.50E-08 | | 2.96E-04 | | 8.22E-06 | | 1.56E-04 | | 13 | | 6.86 |  |
| GOTERM_BP: Biological Process, GOTERM_MF: Molecular Function, GOTERM_CC: Cellular Component, KEGG: Kyoto Encyclopedia of Genes and Genomes. | | | | | | | | | | | | | | | | | | | | | |  |

| **Table S3. Meta-analysis of blood EWAS results in European and Hispanic ancestry without blood cell composition adjustment.** List of the 116 PD-associated CpGs with meta.p-value < 10^-7^ in PEG1 subjects of 508 European and 64 Hispanic ancestry adjusting for age and gender. | | | | | | | | | | | | | | |
| --- | --- | --- | --- | --- | --- | --- | --- | --- | --- | --- | --- | --- | --- | --- |
|  |  |  |  |  | **Relation to UCSC CpG Island** |  |  | | **Caucasian and Hispanic (N=572)** | | **Caucasian (N=508)** | | **Hispanic  (N=64)** | |
|  | **CpG** | **Gene** | **Chr.** | **Position (bp)** |  | **Gene region** | **SNPs** | **SNPs_10** | **meta. Zscore** | **meta. p-value** | **cor** | **p-value** | **cor** | **p-value** |
| 1 | cg02489202 | LARS2 | 3 | 45505334 |  | Body |  |  | -6.58 | 4.67E-11 | -0.30 | 8.34E-11 | -0.35 | 6.56E-03 |
| 2 | cg16580197 |  | 8 | 67841925 | S_Shelf |  |  |  | -6.32 | 2.59E-10 | -0.26 | 2.20E-08 | -0.41 | 1.17E-03 |
| 3 | cg17491368 |  | 1 | 211779938 |  |  |  |  | -6.25 | 4.15E-10 | -0.28 | 1.23E-09 | -0.34 | 7.44E-03 |
| 4 | cg27553947 | CLSTN1 | 1 | 9819767 | N_Shelf | Body | rs76639688 | | -6.11 | 9.88E-10 | -0.25 | 7.17E-08 | -0.40 | 1.55E-03 |
| 5 | cg00175838 | DDAH2 | 6 | 31695027 | N_Shore | Body |  |  | -6.10 | 1.05E-09 | -0.22 | 1.32E-06 | -0.46 | 2.33E-04 |
| 6 | cg11334709 | C1orf200 | 1 | 9716019 | S_Shelf | TSS1500 |  |  | -6.09 | 1.16E-09 | -0.28 | 7.47E-10 | -0.31 | 1.75E-02 |
| 7 | cg04182865 | RNF14 | 5 | 141346431 | N_Shelf | TSS200 |  |  | -6.08 | 1.18E-09 | -0.25 | 5.99E-08 | -0.39 | 1.96E-03 |
| 8 | cg16270399 | LOC284276 | 18 | 74257894 |  | Body |  |  | -6.01 | 1.81E-09 | -0.26 | 8.95E-09 | -0.34 | 7.40E-03 |
| 9 | cg24339704 | GNG7 | 19 | 2529022 | S_Shelf | 5'UTR | rs740054 |  | -5.96 | 2.51E-09 | -0.26 | 2.32E-08 | -0.35 | 5.67E-03 |
| 10 | cg04434593 | LOC100130987 | 11 | 67139546 | N_Shore | Body |  |  | -5.94 | 2.82E-09 | -0.24 | 2.35E-07 | -0.40 | 1.65E-03 |
| 11 | cg15961455 |  | 1 | 23590501 |  |  |  |  | -5.88 | 4.01E-09 | -0.25 | 5.38E-08 | -0.36 | 5.01E-03 |
| 12 | cg23207054 | CSF3 | 17 | 38171530 |  | TSS200 |  |  | -5.87 | 4.42E-09 | -0.25 | 2.79E-08 | -0.34 | 7.57E-03 |
| 13 | cg26474124 |  | 2 | 70368457 | N_Shore |  | rs11685382 |  | -5.86 | 4.52E-09 | -0.26 | 1.66E-08 | -0.33 | 9.98E-03 |
| 14 | cg19879906 |  | 19 | 16392219 | N_Shelf |  |  |  | -5.84 | 5.16E-09 | -0.28 | 1.40E-09 | -0.28 | 3.24E-02 |
| 15 | cg03834767 | CDK14 | 7 | 90794392 |  | 3'UTR |  |  | -5.81 | 6.16E-09 | -0.24 | 1.51E-07 | -0.37 | 3.84E-03 |
| 16 | cg12792363 | LGALS12 | 11 | 63274030 |  | Body |  |  | -5.81 | 6.43E-09 | -0.26 | 1.79E-08 | -0.32 | 1.21E-02 |
| 17 | cg21621482 | SLC38A10 | 17 | 79228937 |  | Body |  |  | -5.77 | 7.78E-09 | -0.24 | 1.15E-07 | -0.36 | 5.28E-03 |
| 18 | cg26963632 |  | 16 | 85558148 |  |  |  |  | -5.77 | 7.89E-09 | -0.25 | 4.49E-08 | -0.34 | 8.77E-03 |
| 19 | cg16301004 | ITPRIP | 10 | 106082537 |  | 5'UTR |  |  | -5.75 | 8.90E-09 | -0.22 | 2.03E-06 | -0.41 | 9.88E-04 |
| 20 | cg16411668 |  | 4 | 123704747 |  |  |  |  | -5.72 | 1.06E-08 | -0.23 | 5.21E-07 | -0.38 | 2.75E-03 |
| 21 | cg03514239 | S100A9 | 1 | 153329781 |  | TSS1500 |  |  | -5.72 | 1.08E-08 | -0.23 | 4.60E-07 | -0.38 | 3.00E-03 |
| 22 | cg01686975 |  | 7 | 138816336 | N_Shelf |  |  |  | -5.71 | 1.14E-08 | -0.23 | 5.02E-07 | -0.38 | 2.98E-03 |
| 23 | cg17879101 | FAM53B | 10 | 126329354 |  | Body |  |  | -5.70 | 1.23E-08 | -0.25 | 3.37E-08 | -0.32 | 1.36E-02 |
| 24 | cg07240557 | STK38L | 12 | 27396937 | N_Shore | TSS200 |  |  | -5.68 | 1.32E-08 | -0.23 | 4.41E-07 | -0.37 | 3.59E-03 |
| 25 | cg04252203 |  | 3 | 194696866 |  |  |  |  | -5.66 | 1.47E-08 | -0.24 | 9.62E-08 | -0.33 | 9.06E-03 |
| 26 | cg25221207 | PVRL1 | 11 | 119555633 |  | Body |  |  | -5.64 | 1.75E-08 | -0.24 | 1.49E-07 | -0.34 | 8.11E-03 |
| 27 | cg23012600 |  | 1 | 244088110 |  |  |  |  | -5.63 | 1.80E-08 | -0.24 | 2.24E-07 | -0.35 | 6.61E-03 |
| 28 | cg08181251 | AVPI1 | 10 | 99443455 | N_Shelf | 5'UTR |  |  | -5.63 | 1.82E-08 | -0.23 | 7.61E-07 | -0.37 | 3.28E-03 |
| 29 | cg08704934 | C3orf21 | 3 | 194826585 |  | Body | rs6799614 | | -5.62 | 1.94E-08 | -0.27 | 2.64E-09 | -0.25 | 5.35E-02 |
| 30 | cg14004161 | SNX22 | 15 | 64442561 | N_Shore | TSS1500 |  |  | -5.61 | 2.01E-08 | -0.25 | 6.84E-08 | -0.32 | 1.33E-02 |
| 31 | cg19709355 | RARA | 17 | 38504102 | S_Shelf | Body |  |  | -5.61 | 2.06E-08 | -0.26 | 2.27E-08 | -0.29 | 2.27E-02 |
| 32 | cg20720686 | POR | 7 | 75582881 |  | 5'UTR | rs41295375 |  | -5.61 | 2.06E-08 | -0.25 | 4.16E-08 | -0.31 | 1.72E-02 |
| 33 | cg02463844 |  | 16 | 88152170 | N_Shore |  |  |  | -5.59 | 2.27E-08 | -0.23 | 9.04E-07 | -0.37 | 3.49E-03 |
| 34 | cg00571483 |  | 1 | 59046173 | S_Shelf |  |  |  | -5.58 | 2.34E-08 | -0.23 | 4.84E-07 | -0.36 | 5.18E-03 |
| 35 | cg16643542 | AZU1 | 19 | 827843 |  | 1stExon | rs34124897 |  | -5.58 | 2.35E-08 | -0.26 | 7.37E-09 | -0.27 | 3.98E-02 |
| 36 | cg14204586 | ARHGEF2 | 1 | 155931858 |  | Body |  |  | -5.57 | 2.51E-08 | -0.22 | 1.83E-06 | -0.38 | 2.42E-03 |
| 37 | cg01357892 | ZXDC | 3 | 126191181 | N_Shelf | Body |  |  | -5.57 | 2.54E-08 | -0.22 | 1.93E-06 | -0.39 | 2.35E-03 |
| 38 | cg23342367 | TPD52 | 8 | 81077480 |  | Body | rs77702759 | | -5.57 | 2.58E-08 | -0.24 | 1.93E-07 | -0.33 | 9.26E-03 |
| 39 | cg25397054 | G12 | 7 | 2874568 |  | Body | rs77747297 |  | -5.56 | 2.64E-08 | -0.23 | 8.25E-07 | -0.37 | 4.13E-03 |
| 40 | cg01152726 | LAMA3 | 18 | 21452844 |  | TSS200 |  |  | 5.56 | 2.76E-08 | 0.27 | 3.64E-09 | 0.25 | 5.75E-02 |
| 41 | cg26542792 | LOC283404 | 12 | 52603567 |  | TSS1500 |  |  | -5.55 | 2.78E-08 | -0.20 | 1.32E-05 | -0.43 | 6.52E-04 |
| 42 | cg02111865 | YPEL5 | 2 | 30371990 | S_Shore | 5'UTR |  |  | -5.55 | 2.82E-08 | -0.24 | 2.37E-07 | -0.34 | 8.82E-03 |
| 43 | cg08698997 | CBFA2T3 | 16 | 88989212 |  | 5'UTR |  |  | -5.55 | 2.88E-08 | -0.21 | 6.48E-06 | -0.41 | 1.14E-03 |
| 44 | cg24866700 | MIR1227 | 19 | 2235103 | N_Shore | TSS1500 | rs34490212 |  | -5.55 | 2.89E-08 | -0.24 | 1.20E-07 | -0.32 | 1.28E-02 |
| 45 | cg26767214 | CHST14 | 15 | 40762864 | N_Shore | TSS1500 |  |  | -5.54 | 2.94E-08 | -0.19 | 3.69E-05 | -0.45 | 2.99E-04 |
| 46 | cg21252105 |  | 9 | 139459307 |  |  |  |  | -5.54 | 3.09E-08 | -0.25 | 5.07E-08 | -0.30 | 2.03E-02 |
| 47 | cg23189692 | EIF4G1 | 3 | 184050393 | N_Shelf | Body |  |  | -5.52 | 3.39E-08 | -0.25 | 3.83E-08 | -0.29 | 2.45E-02 |
| 48 | cg09248276 |  | 1 | 160696468 |  |  |  |  | -5.52 | 3.43E-08 | -0.23 | 4.52E-07 | -0.34 | 7.11E-03 |
| 49 | cg26407841 |  | 7 | 81475720 |  |  |  |  | -5.52 | 3.49E-08 | -0.22 | 1.28E-06 | -0.37 | 3.91E-03 |
| 50 | cg24168413 | FXYD1 | 19 | 35630388 | N_Shore | 5'UTR | rs77395635 |  | -5.50 | 3.79E-08 | -0.26 | 1.53E-08 | -0.27 | 3.91E-02 |
| 51 | cg09032544 | CD247 | 1 | 167487295 |  | Body |  |  | 5.49 | 3.98E-08 | 0.27 | 4.69E-09 | 0.24 | 6.40E-02 |
| 52 | cg08525145 | RXFP4 | 1 | 155911361 |  | TSS200 |  |  | -5.49 | 4.00E-08 | -0.18 | 7.90E-05 | -0.46 | 2.04E-04 |
| 53 | cg03296935 | OSTalpha | 3 | 195946851 |  | Body |  |  | -5.49 | 4.00E-08 | -0.21 | 3.22E-06 | -0.38 | 2.41E-03 |
| 54 | cg19867914 | ARHGAP15 | 2 | 144234430 |  | Body |  |  | -5.49 | 4.02E-08 | -0.23 | 3.39E-07 | -0.33 | 9.31E-03 |
| 55 | cg00827581 | RAVER1 | 19 | 10434088 | Island | Body |  |  | 5.49 | 4.04E-08 | 0.21 | 6.08E-06 | 0.40 | 1.57E-03 |
| 56 | cg22669060 | IFNGR2 | 21 | 34774882 | N_Shore | TSS1500 |  |  | -5.49 | 4.07E-08 | -0.22 | 2.02E-06 | -0.37 | 3.31E-03 |
| 57 | cg16037711 |  | 1 | 59280841 | N_Shore |  | rs2760468 |  | -5.48 | 4.23E-08 | -0.23 | 5.90E-07 | -0.34 | 7.11E-03 |
| 58 | cg26681770 | PMEPA1 | 20 | 56247302 | Island | 5'UTR | rs36008751 |  | -5.48 | 4.23E-08 | -0.26 | 1.40E-08 | -0.26 | 4.32E-02 |
| 59 | cg08669096 |  | 11 | 121229765 | N_Shelf |  |  |  | -5.48 | 4.24E-08 | -0.23 | 6.12E-07 | -0.34 | 6.98E-03 |
| 60 | cg16110541 | GPR177 | 1 | 68679600 |  | Body |  |  | -5.48 | 4.27E-08 | -0.24 | 1.67E-07 | -0.32 | 1.41E-02 |
| 61 | cg12707346 |  | 12 | 64960957 |  |  |  |  | -5.47 | 4.40E-08 | -0.24 | 1.22E-07 | -0.31 | 1.68E-02 |
| 62 | cg13049483 | RAB31 | 18 | 9712461 | S_Shelf | Body |  |  | -5.47 | 4.42E-08 | -0.21 | 4.00E-06 | -0.39 | 2.26E-03 |
| 63 | cg15209885 | CBX2 | 17 | 77753199 | S_Shore | Body | rs72231015 |  | -5.47 | 4.54E-08 | -0.25 | 3.89E-08 | -0.28 | 2.92E-02 |
| 64 | cg14023999 |  | 15 | 90543224 | N_Shore |  |  |  | -5.47 | 4.62E-08 | -0.25 | 4.68E-08 | -0.29 | 2.72E-02 |
| 65 | cg04988978 | MPO | 17 | 56359578 | S_Shelf | TSS1500 | rs61007103 | | -5.46 | 4.69E-08 | -0.23 | 7.75E-07 | -0.35 | 6.56E-03 |
| 66 | cg05468843 | IL10RA | 11 | 117857990 | S_Shore | Body |  |  | -5.46 | 4.87E-08 | -0.23 | 6.51E-07 | -0.34 | 7.45E-03 |
| 67 | cg01966117 | STAB1 | 3 | 52528714 |  | TSS1500 |  |  | -5.45 | 4.99E-08 | -0.23 | 3.83E-07 | -0.33 | 1.01E-02 |
| 68 | cg04496824 | MTF1 | 1 | 38276835 | S_Shelf | 3'UTR | rs76151238 | | -5.44 | 5.19E-08 | -0.22 | 1.38E-06 | -0.36 | 5.03E-03 |
| 69 | cg14224600 | TESC | 12 | 117477926 | S_Shore | Body |  |  | -5.44 | 5.30E-08 | -0.23 | 3.99E-07 | -0.33 | 1.04E-02 |
| 70 | cg08400494 | CARS2 | 13 | 111318490 |  | Body |  |  | -5.44 | 5.32E-08 | -0.25 | 5.34E-08 | -0.28 | 2.80E-02 |
| 71 | cg06097213 | LOC285830 | 6 | 29717651 | S_Shore | TSS1500 |  |  | -5.44 | 5.42E-08 | -0.23 | 3.98E-07 | -0.33 | 1.05E-02 |
| 72 | cg06746829 | PADI2 | 1 | 17424524 |  | Body | rs2746507 | | -5.44 | 5.43E-08 | -0.22 | 2.40E-06 | -0.37 | 3.70E-03 |
| 73 | cg05657656 |  | 3 | 150093970 |  |  | rs34177912 | | -5.43 | 5.57E-08 | -0.21 | 3.62E-06 | -0.38 | 2.90E-03 |
| 74 | cg20100745 | NDRG1 | 8 | 134307728 | N_Shore | 5'UTR |  |  | -5.43 | 5.63E-08 | -0.23 | 3.53E-07 | -0.32 | 1.15E-02 |
| 75 | cg23281529 | RASA3 | 13 | 114823829 |  | Body |  |  | -5.43 | 5.66E-08 | -0.22 | 1.23E-06 | -0.35 | 5.76E-03 |
| 76 | cg06437100 | OTOA | 16 | 21767742 | N_Shore | Body | rs459377 | rs465386 | -5.43 | 5.71E-08 | -0.23 | 9.28E-07 | -0.35 | 6.83E-03 |
| 77 | cg26489413 | AMPD3 | 11 | 10476976 |  | 1stExon |  |  | -5.42 | 6.01E-08 | -0.25 | 4.15E-08 | -0.27 | 3.38E-02 |
| 78 | cg21815704 | GLRX2 | 1 | 193075249 | S_Shore | TSS1500 |  |  | -5.42 | 6.03E-08 | -0.25 | 7.41E-08 | -0.29 | 2.61E-02 |
| 79 | cg04772575 | ABCB9 | 12 | 123431865 |  | Body | rs73408862 |  | -5.42 | 6.08E-08 | -0.28 | 4.33E-10 | -0.18 | 1.73E-01 |
| 80 | cg12007048 | CTSD | 11 | 1785701 | S_Shore | TSS1500 |  |  | -5.41 | 6.14E-08 | -0.25 | 7.47E-08 | -0.29 | 2.63E-02 |
| 81 | cg25139649 | SKI | 1 | 2165579 |  | Body |  |  | -5.41 | 6.18E-08 | -0.22 | 1.06E-06 | -0.35 | 6.69E-03 |
| 82 | cg26793227 | EPHA2 | 1 | 16483658 | S_Shore | TSS1500 | rs57602506 |  | -5.40 | 6.58E-08 | -0.26 | 8.17E-09 | -0.24 | 6.86E-02 |
| 83 | cg14642045 | UNG | 12 | 109538736 | S_Shelf | Body | rs3219221 |  | -5.40 | 6.58E-08 | -0.25 | 5.17E-08 | -0.28 | 3.24E-02 |
| 84 | cg21577598 | CCDC57 | 17 | 80084751 | N_Shore | Body |  |  | 5.40 | 6.72E-08 | 0.26 | 8.11E-09 | 0.24 | 6.96E-02 |
| 85 | cg18463607 | EXOC1 | 4 | 56718320 | N_Shore | TSS1500 |  |  | 5.40 | 6.76E-08 | 0.25 | 8.54E-08 | 0.29 | 2.63E-02 |
| 86 | cg00115639 |  | 17 | 54701692 |  |  |  |  | -5.40 | 6.78E-08 | -0.20 | 1.04E-05 | -0.40 | 1.64E-03 |
| 87 | cg19690214 | IPCEF1 | 6 | 154678326 |  | TSS1500 |  |  | -5.40 | 6.80E-08 | -0.23 | 5.57E-07 | -0.33 | 1.03E-02 |
| 88 | cg07029980 |  | 7 | 104640734 |  |  |  |  | -5.40 | 6.82E-08 | -0.21 | 6.60E-06 | -0.39 | 2.28E-03 |
| 89 | cg08885142 |  | 11 | 69264296 |  |  |  |  | -5.39 | 6.96E-08 | -0.22 | 1.75E-06 | -0.35 | 5.44E-03 |
| 90 | cg08618878 | CDH15 | 16 | 89261116 | S_Shore | Body |  |  | -5.39 | 7.01E-08 | -0.22 | 2.21E-06 | -0.36 | 4.74E-03 |
| 91 | cg01657758 | SORL1 | 11 | 121349740 |  | Body |  |  | -5.39 | 7.04E-08 | -0.26 | 1.46E-08 | -0.25 | 5.71E-02 |
| 92 | cg14039779 | DUSP3 | 17 | 41857714 | S_Shore | TSS1500 |  |  | -5.39 | 7.07E-08 | -0.23 | 8.49E-07 | -0.34 | 8.40E-03 |
| 93 | cg01606027 | LIMK2 | 22 | 31607212 | N_Shore | TSS1500 |  |  | -5.39 | 7.11E-08 | -0.23 | 5.11E-07 | -0.33 | 1.12E-02 |
| 94 | cg23956760 | SLC25A42 | 19 | 19178708 | S_Shelf | 5'UTR |  |  | -5.38 | 7.50E-08 | -0.22 | 2.78E-06 | -0.36 | 4.33E-03 |
| 95 | cg27031099 |  | 8 | 126620534 |  |  |  |  | -5.38 | 7.55E-08 | -0.21 | 5.37E-06 | -0.38 | 2.84E-03 |
| 96 | cg03452174 | RAB34 | 17 | 27045113 | N_Shore | Body |  |  | -5.38 | 7.59E-08 | -0.19 | 3.12E-05 | -0.42 | 7.95E-04 |
| 97 | cg15658793 | NFIC | 19 | 3398810 | Island | Body |  |  | -5.38 | 7.61E-08 | -0.24 | 2.10E-07 | -0.30 | 1.85E-02 |
| 98 | cg06719445 | FOXN3 | 14 | 89925468 |  | 5'UTR |  |  | -5.37 | 7.80E-08 | -0.20 | 9.90E-06 | -0.39 | 1.92E-03 |
| 99 | cg12063992 | PTPN9 | 15 | 75865604 |  | Body | rs80220483 |  | -5.37 | 7.81E-08 | -0.21 | 5.25E-06 | -0.38 | 2.97E-03 |
| 100 | cg10752406 | AZU1 | 19 | 827776 |  | TSS200 |  |  | -5.37 | 7.84E-08 | -0.27 | 4.96E-09 | -0.22 | 9.04E-02 |
| 101 | cg15401418 | SEPT9 | 17 | 75316383 |  | 5'UTR |  |  | -5.37 | 7.86E-08 | -0.23 | 4.60E-07 | -0.32 | 1.27E-02 |
| 102 | cg23829102 |  | 4 | 124476694 |  |  |  |  | -5.37 | 7.98E-08 | -0.22 | 2.76E-06 | -0.36 | 4.57E-03 |
| 103 | cg27640064 | FOXK1 | 7 | 4752983 |  | Body | rs77026339 | | -5.36 | 8.13E-08 | -0.25 | 3.50E-08 | -0.26 | 4.36E-02 |
| 104 | cg05496814 |  | 5 | 111445415 |  |  |  |  | -5.36 | 8.34E-08 | -0.24 | 2.45E-07 | -0.30 | 1.82E-02 |
| 105 | cg24821554 | GUCY1B2 | 13 | 51639953 |  | Body |  |  | -5.36 | 8.41E-08 | -0.21 | 5.04E-06 | -0.37 | 3.23E-03 |
| 106 | cg04992150 | NUP210 | 3 | 13457267 | N_Shelf | Body |  |  | -5.35 | 8.57E-08 | -0.20 | 1.17E-05 | -0.39 | 1.85E-03 |
| 107 | cg08380252 | SCN1B | 19 | 35530254 | N_Shore | Body | rs34790884 | rs72550244 | -5.35 | 8.81E-08 | -0.23 | 3.11E-07 | -0.31 | 1.68E-02 |
| 108 | cg19081101 | CHI3L1 | 1 | 203156625 |  | TSS1500 |  |  | -5.35 | 8.88E-08 | -0.25 | 3.05E-08 | -0.26 | 4.87E-02 |
| 109 | cg22358291 | WDR1 | 4 | 10101553 |  | Body |  |  | 5.35 | 8.99E-08 | 0.26 | 1.38E-08 | 0.24 | 6.70E-02 |
| 110 | cg21495704 | TYROBP | 19 | 36399346 |  | TSS200 | rs56006731 | | -5.35 | 9.01E-08 | -0.24 | 9.02E-08 | -0.28 | 3.08E-02 |
| 111 | cg16349093 |  | 5 | 90485840 |  |  |  |  | -5.34 | 9.16E-08 | -0.20 | 1.14E-05 | -0.39 | 1.98E-03 |
| 112 | cg02268192 | RIN3 | 14 | 92981666 | S_Shore | Body |  |  | -5.34 | 9.28E-08 | -0.24 | 2.85E-07 | -0.30 | 1.81E-02 |
| 113 | cg05007997 | SLC11A1 | 2 | 219246985 |  | 1stExon |  |  | -5.34 | 9.41E-08 | -0.23 | 8.27E-07 | -0.33 | 1.05E-02 |
| 114 | cg01058360 | PRKAG2 | 7 | 151442371 |  | Body |  |  | -5.34 | 9.51E-08 | -0.20 | 2.04E-05 | -0.40 | 1.34E-03 |
| 115 | cg10884953 | GNG7 | 19 | 2579745 | Island | 5'UTR |  |  | -5.33 | 9.77E-08 | -0.21 | 6.09E-06 | -0.37 | 3.21E-03 |
| 116 | cg16451527 | ARHGEF3 | 3 | 56889795 |  | Body |  |  | -5.33 | 9.89E-08 | -0.20 | 9.74E-06 | -0.39 | 2.36E-03 |
| Chr.: Chromosome, bp: base pair, TSS: transcription start site, TSS1500: within 1500 bps of a TSS, TSS200: within 200 bps of a TSS, UTR: untranslated region, SNPs: listing dbSNP entries within a probe, SNPs_10: listing dbSNP entries within 10 bp of the CpG site. | | | | | | | | | | | | | | |

| **Table S4. List of the 19 PD-associated CpGs in blood based methylation analyses with cell composition adjustment.** These are CpGs with p-value < 5x10^-6^ in blood based DNA methylation analyses for 508 PEG1 subjects of European ancestry adjusting for age, gender, and cell composition. | | | | | | | | | | |
| --- | --- | --- | --- | --- | --- | --- | --- | --- | --- | --- |
|  | **CpG** | **Gene** | **Chr.** | **Position (bp)** | **Relation to UCSC CpG Island** | **Gene region** | **SNPs** | **SNPs_10** | **cor** | **p-value** |
| 1 | cg05001044 | MIR1977 | 1 | 567312 |  | TSS1500 |  |  | -0.24 | 1.66E-07 |
| 2 | cg27211284 |  | 2 | 200524012 | Island |  |  |  | 0.24 | 1.77E-07 |
| 3 | cg05853632 | RNF39 | 6 | 30043273 | Island | 1stExon |  |  | 0.24 | 1.93E-07 |
| 4 | cg03830712 |  | 10 | 17347391 |  |  | rs10904927 |  | 0.23 | 4.84E-07 |
| 5 | cg09136896 | RRP12 | 10 | 99160663 | N_Shore | Body | rs10882926 |  | 0.23 | 5.85E-07 |
| 6 | cg05683049 | CDH10 | 5 | 24645093 |  | TSS200 |  |  | 0.23 | 6.94E-07 |
| 7 | cg18809855 | CGNL1 | 15 | 57668539 | Island | TSS200 |  |  | 0.23 | 8.87E-07 |
| 8 | cg13286582 | CDC42EP3 | 2 | 37883934 |  | 5'UTR |  |  | -0.23 | 9.09E-07 |
| 9 | cg10248492 |  | 1 | 228647248 | S_Shore |  |  |  | 0.22 | 1.08E-06 |
| 10 | cg03958663 |  | 6 | 41262467 |  |  |  |  | -0.22 | 1.46E-06 |
| 11 | cg09040699 | EIF3F | 11 | 8009242 | Island | 1stExon |  |  | 0.22 | 2.07E-06 |
| 12 | cg01685644 | MBLAC1 | 7 | 99724788 | Island | 5'UTR |  |  | 0.22 | 2.31E-06 |
| 13 | cg25679366 | ABCB4 | 7 | 87104879 | Island | 5'UTR |  | rs34690247 | 0.22 | 2.57E-06 |
| 14 | cg26544742 |  | 6 | 27533825 |  |  |  |  | 0.22 | 2.86E-06 |
| 15 | cg07391218 | HPCAL1 | 2 | 10442879 | Island | TSS1500 |  |  | 0.21 | 3.35E-06 |
| 16 | cg26560222 | DMRTA2 | 1 | 50888826 | Island | 1stExon |  |  | 0.21 | 3.37E-06 |
| 17 | cg05624199 |  | 19 | 41168318 | N_Shore |  |  |  | -0.21 | 4.07E-06 |
| 18 | cg19342159 | C19orf60 | 19 | 18699433 | Island | TSS200 |  |  | 0.21 | 4.26E-06 |
| 19 | cg11266874 | ARL13B | 3 | 93699352 | Island | Body |  |  | 0.21 | 4.46E-06 |
| Chr.: Chromosome, bp: base pair, TSS: transcription start site, TSS1500: within 1500 bps of a TSS, TSS200: within 200 bps of a TSS, UTR: untranslated region, SNPs: listing dbSNP entries within a probe, SNPs_10: listing dbSNP entries within 10 bp of the CpG site.  ^a^ age, gender, and race adjusted. | | | | | | | | | | |

| **Table S5. Gene set enrichment analysis for PD-associated CpGs in blood with cell composition adjustment.**  (a) Gene set enrichment analysis for the top 2,000 PD-associated CpGs in blood based DNA methylation analyses for 508 PEG1 subjects of European ancestry adjusting for age, gender and cell composition and stratified by direction of methylation changes. | | | | | | | | | |
| --- | --- | --- | --- | --- | --- | --- | --- | --- | --- |
| **Rank** | | **Category** | **Term** | **p-value** | **Bonferroni** | **Benjamini** | **FDR** | **Overlap Genes (N)** | **Fold Enrichment** |
| ***All CpGs (2,000 CpGs in 1,434 genes)*** | | | |  |  |  |  |  |  |
| 1 | | GOTERM_MF_FAT | GO:0016564~transcription repressor activity | 7.58E-06 | 7.96E-03 | 3.99E-03 | 1.21E-02 | 47 | 2.00 |
| 2 | | GOTERM_BP_FAT | GO:0001501~skeletal system development | 6.71E-05 | 2.01E-01 | 3.66E-02 | 1.22E-01 | 45 | 1.87 |
| 3 | | GOTERM_BP_FAT | GO:0051960~regulation of nervous system development | 1.05E-04 | 2.95E-01 | 4.28E-02 | 1.91E-01 | 31 | 2.14 |
| 4 | | GOTERM_BP_FAT | GO:0010629~negative regulation of gene expression | 1.49E-04 | 3.91E-01 | 3.75E-02 | 2.70E-01 | 62 | 1.63 |
| 5 | | GOTERM_BP_FAT | GO:0030182~neuron differentiation | 2.21E-04 | 5.22E-01 | 4.80E-02 | 4.02E-01 | 55 | 1.66 |
| 6 | | GOTERM_BP_FAT | GO:0016055~Wnt receptor signaling pathway | 4.22E-02 | 1.00E+00 | 5.87E-01 | 5.44E+01 | 17 | 1.69 |
| ***Hypomethylated-CpGs (612 CpGs in 382 genes)*** | | | |  |  |  |  |  |  |
| 1 | GOTERM_MF_FAT | | GO:0016564~transcription repressor activity | 4.93E-04 | 2.21E-01 | 1.17E-01 | 7.09E-01 | 17 | 2.74 |
| 2 | GOTERM_BP_FAT | | GO:0016481~negative regulation of transcription | 2.81E-03 | 9.95E-01 | 5.38E-01 | 4.70E+00 | 20 | 2.13 |
| 3 | GOTERM_BP_FAT | | GO:0010975~regulation of neuron projection development | 3.00E-03 | 9.97E-01 | 5.15E-01 | 5.02E+00 | 7 | 4.88 |
| 4 | GOTERM_MF_FAT | | GO:0004674~protein serine/threonine kinase activity | 4.75E-03 | 9.10E-01 | 4.52E-01 | 6.64E+00 | 18 | 2.13 |
| 5 | GOTERM_MF_FAT | | GO:0005524~ATP binding | 4.86E-03 | 9.15E-01 | 3.89E-01 | 6.79E+00 | 44 | 1.52 |
| ***Hypermethylated-CpGs (1,388 CpGs in 1,078 genes)*** | | | |  |  |  |  |  |  |
| 1 | GOTERM_BP_FAT | | GO:0001501~skeletal system development | 5.01E-05 | 1.33E-01 | 2.82E-02 | 8.97E-02 | 37 | 2.06 |
| 2 | GOTERM_MF_FAT | | GO:0003700~transcription factor activity | 6.20E-05 | 5.53E-02 | 2.80E-02 | 9.68E-02 | 84 | 1.54 |
| 3 | GOTERM_MF_FAT | | GO:0043565~sequence-specific DNA binding | 4.39E-04 | 3.31E-01 | 1.26E-01 | 6.84E-01 | 55 | 1.62 |
| 4 | GOTERM_BP_FAT | | GO:0030323~respiratory tube development | 1.63E-03 | 9.90E-01 | 1.91E-01 | 2.88E+00 | 15 | 2.62 |
| GOTERM_BP: Biological Process, GOTERM_MF: Molecular Function. | | | | | | | | | |

| (b) Gene set enrichment analysis for CpGs in the 3 PD-associated modules in blood based DNA methylation analyses for 508 PEG1 subjects of European ancestry adjusting for age, gender and cell composition (module p-value cutoff = 5x10^-4^) | | | | | | | | | | | |  |
| --- | --- | --- | --- | --- | --- | --- | --- | --- | --- | --- | --- | --- |
| **Rank** | **Category** | **Term** | **p-value** | **Bonferroni** | | **Benjamini** | | **FDR** | **Overlap Genes (N)** | **Fold Enrichment** | | |
| ***Hypomethylated-module*** | | | | | | | | | | | | |
|  | ***Mediumorchid module (Cor=-0.17, p-value=2x10^-4^; 60 CpGs in 45 genes)*** | | | |  |  |  | |  | |  | |
| 1 | GOTERM_MF_FAT | GO:0046872~metal ion binding | 4.32E-03 | | 4.54E-01 | 4.54E-01 | 4.98E+00 | | 18 | | 1.82 | |
| 2 | GOTERM_MF_FAT | GO:0005509~calcium ion binding | 5.73E-02 | | 1.00E+00 | 8.08E-01 | 5.01E+01 | | 6 | | 2.73 | |
| ***Hypermethylated-modules*** | |  |  | |  |  |  | |  | |  | |
|  | ***Thistle1 module (Cor=0.15, pvalue=8x10^-4^; 138 CpGs in 92 genes)*** | |  | |  |  |  | |  | |  | |
| 1 | GOTERM_BP_FAT | GO:0030182~neuron differentiation | 5.91E-11 | | 5.12E-08 | 5.12E-08 | 9.17E-08 | | 17 | | 8.47 | |
| 2 | GOTERM_BP_FAT | GO:0051960~regulation of nervous system development | 1.78E-07 | | 1.54E-04 | 5.13E-05 | 2.76E-04 | | 10 | | 11.36 | |
| 3 | GOTERM_MF_FAT | GO:0043565~sequence-specific DNA binding | 5.08E-07 | | 8.38E-05 | 8.38E-05 | 6.17E-04 | | 16 | | 4.89 | |
| 4 | GOTERM_BP_FAT | GO:0048663~neuron fate commitment | 1.18E-06 | | 1.02E-03 | 1.70E-04 | 1.83E-03 | | 6 | | 31.17 | |
| 5 | GOTERM_BP_FAT | GO:0045449~regulation of transcription | 7.14E-05 | | 5.99E-02 | 4.11E-03 | 1.11E-01 | | 26 | | 2.18 | |
| 6 | GOTERM_BP_FAT | GO:0031175~neuron projection development | 1.01E-03 | | 5.82E-01 | 2.53E-02 | 1.55E+00 | | 7 | | 5.97 | |
|  | ***Salmon4 module (Cor=0.15, pvalue=8x10^-4^; 1831 CpGs in 1282 genes)*** | |  | |  |  |  | |  | |  | |
| 1 | GOTERM_MF_FAT | GO:0008134~transcription factor binding | 1.51E-07 | | 1.59E-04 | 7.94E-05 | 2.41E-04 | | 67 | | 1.96 | |
| 2 | GOTERM_MF_FAT | GO:0016564~transcription repressor activity | 1.30E-05 | | 1.35E-02 | 2.72E-03 | 2.07E-02 | | 43 | | 2.04 | |
| 3 | GOTERM_BP_FAT | GO:0016055~Wnt receptor signaling pathway | 2.47E-05 | | 7.46E-02 | 1.54E-02 | 4.46E-02 | | 24 | | 2.68 | |
| 4 | GOTERM_BP_FAT | GO:0060070~Wnt receptor signaling pathway through beta-catenin | 6.51E-05 | | 1.85E-01 | 1.69E-02 | 1.18E-01 | | 8* | | 6.99 | |
| 5 | GOTERM_BP_FAT | GO:0010629~negative regulation of gene expression | 7.32E-05 | | 2.06E-01 | 1.52E-02 | 1.32E-01 | | 58 | | 1.71 | |
| GOTERM_BP: Biological Process, GOTERM_MF: Molecular Function.  *Genes: APC, AXIN1, CTNNB1, PTPRU, WNT9A, TBL1X, TBL1XR1, TCF7L2 | | | | | | | | | | | | |

| **Table S6. Meta-analysis of blood based EWAS results in subjects with European and Hispanic ancestry with cell composition adjustment.** List of the 2 PD-associated CpGs with meta.p-value < 5x10^-6^ in PEG1 for 508 European ancestry and 64 Hispanic ancestry subjects adjusting for age, gender and cell composition. | | | | | | | | | | | | | | |
| --- | --- | --- | --- | --- | --- | --- | --- | --- | --- | --- | --- | --- | --- | --- |
|  |  |  |  |  | **Relation to UCSC CpG Island** |  |  | | **Caucasian and Hispanic (N=572)** | | **Caucasian  (N=508)** | | **Hispanic  (N=64)** | |
|  | **CpG** | **Gene** | **Chr.** | **Position (bp)** |  | **Gene region** | **SNPs** | **SNPs_10** | **meta. Zscore** | **meta. p-value** | **cor** | **p-value** | **cor** | **p-value** |
| 1 | cg27191131 | CEP63 | 3 | 134205014 | Island | 5'UTR |  | rs35860255 | 4.82 | 1.45E-06 | 0.19 | 5.17E-05 | 0.35 | 6.71E-03 |
| 2 | cg13322234 |  | 8 | 142632593 | S_Shelf |  |  |  | -4.69 | 2.75E-06 | -0.19 | 2.50E-05 | -0.31 | 1.78E-02 |
| Chr.: Chromosome, bp: base pair, UTR: untranslated region, SNPs: listing dbSNP entries within a probe, SNPs_10: listing dbSNP entries within 10 bp of the CpG site. | | | | | | | | | | | | | | |

| **Table S7. Gene set enrichment analysis for PD-associated CpGs in saliva.**  (a) Gene set enrichment analysis for the top 2,000 PD-associated CpGs in saliva based DNA methylation analyses for 259 PEG2 subjects adjusting for age, gender, and race and stratified by the direction of methylation changes. | | | | | | | | | | | | | | | |
| --- | --- | --- | --- | --- | --- | --- | --- | --- | --- | --- | --- | --- | --- | --- | --- |
| **Rank** | **Category** | **Term** | **p-value** | | **Bonferroni** | | | **Benjamini** | **FDR** | **Overlap Genes (N)** | **Fold Enrichment** | | |  |  |
| ***All CpGs (2,000 CpGs in 1,226 genes)*** | | | |  | | |  |  |  |  | | |  |  |  |
| 1 | GOTERM_MF_FAT | GO:0043565~sequence-specific DNA binding | 1.46E-10 | | | 1.42E-07 | | 1.42E-07 | 2.29E-07 | 85 | | 2.07 | |  |  |
| 2 | GOTERM_BP_FAT | GO:0030182~neuron differentiation | 1.61E-08 | | | 5.11E-05 | | 5.11E-05 | 2.91E-05 | 62 | | 2.15 | |  |  |
| 3 | GOTERM_BP_FAT | GO:0030902~hindbrain development | 1.00E-06 | | | 3.19E-03 | | 7.99E-04 | 1.82E-03 | 17 | | 4.31 | |  |  |
| 4 | GOTERM_BP_FAT | GO:0010628~positive regulation of gene expression | 2.00E-06 | | | 6.35E-03 | | 7.07E-04 | 3.63E-03 | 69 | | 1.81 | |  |  |
| 5 | GOTERM_BP_FAT | GO:0006357~regulation of transcription from RNA polymerase II promoter | 5.38E-06 | | | 1.70E-02 | | 1.31E-03 | 9.75E-03 | 80 | | 1.67 | |  |  |
| 6 | GOTERM_BP_FAT | GO:0048812~neuron projection morphogenesis | 9.04E-06 | | | 2.84E-02 | | 1.92E-03 | 1.64E-02 | 33 | | 2.36 | |  |  |
| ***Hypomethylated-CpGs (1,760 CpGs in 1,054 genes)*** | | |  | | |  | |  |  |  | |  | |  |  |
| 1 | GOTERM_MF_FAT | GO:0043565~sequence-specific DNA binding | 6.59E-11 | | | 6.17E-08 | | 6.17E-08 | 1.03E-07 | 78 | | 2.20 | |  |  |
| 2 | GOTERM_BP_FAT | GO:0030182~neuron differentiation | 3.55E-09 | | | 1.09E-05 | | 1.09E-05 | 6.41E-06 | 58 | | 2.32 | |  |  |
| 3 | GOTERM_BP_FAT | GO:0030902~hindbrain development | 1.47E-07 | | | 4.49E-04 | | 1.50E-04 | 2.65E-04 | 17 | | 4.97 | |  |  |
| 4 | GOTERM_BP_FAT | GO:0006357~regulation of transcription from RNA polymerase II promoter | 1.28E-06 | | | 3.90E-03 | | 5.58E-04 | 2.30E-03 | 74 | | 1.79 | |  |  |
| 5 | GOTERM_BP_FAT | GO:0048812~neuron projection morphogenesis | 3.88E-06 | | | 1.18E-02 | | 1.48E-03 | 7.00E-03 | 31 | | 2.55 | |  |  |
| 6 | GOTERM_BP_FAT | GO:0010628~positive regulation of gene expression | 9.90E-06 | | | 2.98E-02 | | 2.16E-03 | 1.79E-02 | 60 | | 1.81 | |  |  |
| ***Hypermethylated-CpGs (240 CpGs in 195 genes)*** | | |  | | |  | |  |  |  | |  | |  |  |
| 1 | GOTERM_MF_FAT | GO:0016563~transcription activator activity | 3.68E-04 | | | 1.15E-01 | | 1.15E-01 | 4.98E-01 | 14 | | 3.24 | |  |  |
| 2 | GOTERM_CC_FAT | GO:0031981~nuclear lumen | 5.79E-04 | | | 1.19E-01 | | 6.15E-02 | 7.34E-01 | 29 | | 1.95 | |  |  |
| 3 | GOTERM_CC_FAT | GO:0044450~microtubule organizing center part | 1.93E-02 | | | 9.86E-01 | | 3.47E-01 | 2.19E+01 | 4 | | 6.97 | |  |  |
| GOTERM_BP: Biological Process, GOTERM_MF: Molecular Function, GOTERM_CC: Cellular Component. | | | | | | | | | | | | | |  |  |

| (b) Gene set enrichment analysis for CpGs in the 10 PD-associated modules in saliva based DNA methylation analyses for 259 PEG2 subjects adjusting for age, gender, and race (module p-value cutoff = 1x10^-3^) | | | | | | | | | | | | |  |  |  |  |
| --- | --- | --- | --- | --- | --- | --- | --- | --- | --- | --- | --- | --- | --- | --- | --- | --- |
| **Rank** | | | **Category** | **Term** | **p-value** | **Bonferroni** | **Benjamini** | **FDR** | | **Overlap Genes (N)** | | **Fold Enrichment** |  |  |  |  |
| ***Hypomethylated-module*** | | | | | | | | | | | | |  |  |  |  |
|  | | ***Magenta module (Cor=-0.22, p-value=3x10^-4^; 3,000 CpGs in 1,523 genes; 4,954 CpGs in total)*** | | | | | | | | | | |  |  |  |  |
| 1 | | GOTERM_BP_FAT | | GO:0016339~calcium-dependent cell-cell adhesion | 2.44E-06 | 8.19E-03 | 8.19E-03 | 4.45E-03 | 11 | | 6.39 | |  |  |  |  |
| 2 | | GOTERM_MF_FAT | | GO:0005509~calcium ion binding | 8.06E-06 | 8.17E-03 | 8.17E-03 | 1.28E-02 | 106 | | 1.53 | |  |  |  |  |
| 3 | | GOTERM_BP_FAT | | GO:0030182~neuron differentiation | 2.67E-05 | 8.61E-02 | 2.96E-02 | 4.87E-02 | 58 | | 1.77 | |  |  |  |  |
| 4 | | GOTERM_BP_FAT | | GO:0048667~cell morphogenesis involved in neuron differentiation | 8.01E-05 | 2.37E-01 | 3.32E-02 | 1.46E-01 | 33 | | 2.11 | |  |  |  |  |
|  | | ***Salmon module (Cor=-0.21, p-value=9x10^-4^; 2,053 CpGs in 1,239 genes)*** | | | | | | | | | | |  |  |  |  |
| 1 | | GOTERM_BP_FAT | | GO:0030182~neuron differentiation | 8.18E-05 | 2.22E-01 | 1.18E-01 | 1.48E-01 | 50 | | 1.79 | |  |  |  |  |
| 2 | | GOTERM_BP_FAT | | GO:0031175~neuron projection development | 1.95E-04 | 4.50E-01 | 1.39E-01 | 3.51E-01 | 33 | | 2.02 | |  |  |  |  |
| 3 | | GOTERM_CC_FAT | | GO:0045211~postsynaptic membrane | 3.97E-04 | 1.64E-01 | 1.64E-01 | 5.63E-01 | 21 | |  | |  |  |  |  |
| 4 | | KEGG_PATHWAY | | hsa04080:Neuroactive ligand-receptor interaction | 5.88E-04 | 8.99E-02 | 8.99E-02 | 7.08E-01 | 30 | | 1.95 | |  |  |  |  |
| 5 | | GOTERM_BP_FAT | | GO:0006816~calcium ion transport | 6.69E-04 | 8.71E-01 | 1.28E-01 | 1.20E+00 | 21 | | 2.32 | |  |  |  |  |
| 6 | | GOTERM_BP_FAT | | GO:0016337~cell-cell adhesion | 7.42E-04 | 8.97E-01 | 1.25E-01 | 1.33E+00 | 33 | | 1.87 | |  |  |  |  |
| 7 | | GOTERM_BP_FAT | | GO:0048667~cell morphogenesis involved in neuron differentiation | 1.76E-03 | 9.95E-01 | 1.94E-01 | 3.12E+00 | 26 | | 1.95 | |  |  |  |  |
| 8 | | GOTERM_MF_FAT | | GO:0008092~cytoskeletal protein binding | 1.77E-03 | 8.16E-01 | 1.71E-01 | 2.75E+00 | 51 | | 1.55 | |  |  |  |  |
| 9 | | GOTERM_BP_FAT | | GO:0007268~synaptic transmission | 2.61E-03 | 1.00E+00 | 2.22E-01 | 4.61E+00 | 33 | |  | |  |  |  |  |
| 10 | | GOTERM_BP_FAT | | GO:0019226~transmission of nerve impulse | 3.05E-03 | 1.00E+00 | 2.35E-01 | 5.36E+00 | 37 | | 1.66 | |  |  |  |  |
|  | | ***Brown module (Cor=-0.21, p-value=8x10^-4^; 3,000 CpGs in 1,523 genes; 37,210 CpGs in total)*** | | | | | | | | | | |  |  |  |  |
| 1 | | GOTERM_BP_FAT | | GO:0045321~leukocyte activation | 2.94E-13 | 1.04E-09 | 1.04E-09 | 5.39E-10 | 57 | | 2.92 | |  |  |  |  |
| 2 | | GOTERM_BP_FAT | | GO:0006955~immune response | 1.60E-12 | 5.67E-09 | 1.89E-09 | 2.94E-09 | 111 | | 1.99 | |  |  |  |  |
| 3 | | GOTERM_BP_FAT | | GO:0046649~lymphocyte activation | 1.47E-10 | 5.21E-07 | 1.30E-07 | 2.70E-07 | 46 | | 2.86 | |  |  |  |  |
| 4 | | GOTERM_BP_FAT | | GO:0042110~T cell activation | 7.95E-10 | 2.82E-06 | 5.63E-07 | 1.46E-06 | 34 | | 3.34 | |  |  |  |  |
| 5 | | GOTERM_BP_FAT | | GO:0001817~regulation of cytokine production | 3.40E-08 | 1.20E-04 | 2.01E-05 | 6.24E-05 | 39 | | 2.67 | |  |  |  |  |
| 6 | | GOTERM_BP_FAT | | GO:0043065~positive regulation of apoptosis | 2.33E-06 | 8.22E-03 | 4.13E-04 | 4.28E-03 | 64 | | 1.84 | |  |  |  |  |
| 7 | | GOTERM_BP_FAT | | GO:0006954~inflammatory response | 2.98E-06 | 1.05E-02 | 4.22E-04 | 5.46E-03 | 52 | | 1.98 | |  |  |  |  |
| 8 | | KEGG_PATHWAY | | hsa04650:Natural killer cell mediated cytotoxicity | 3.03E-05 | 5.32E-03 | 2.66E-03 | 3.72E-02 | 28 | | 2.36 | |  |  |  |  |
|  | | ***Green module (Cor=-0.24, p-value=1x10^-4^; 3,000 CpGs in 1,694 genes; 8,539 CpGs in total)*** | | | | | | | | | | |  |  |  |  |
| 1 | | GOTERM_MF_FAT | | GO:0043565~sequence-specific DNA binding | 1.81E-08 | 2.07E-05 | 2.07E-05 | 2.91E-05 | 95 | | 1.80 | |  |  |  |  |
| 2 | | GOTERM_BP_FAT | | GO:0030182~neuron differentiation | 1.08E-06 | 3.83E-03 | 3.83E-03 | 1.98E-03 | 71 | | 1.81 | |  |  |  |  |
| 3 | | GOTERM_BP_FAT | | GO:0001501~skeletal system development | 7.04E-06 | 2.47E-02 | 3.57E-03 | 1.29E-02 | 54 | | 1.89 | |  |  |  |  |
| 4 | | GOTERM_BP_FAT | | GO:0050769~positive regulation of neurogenesis | 9.89E-06 | 3.45E-02 | 3.51E-03 | 1.81E-02 | 18 | | 3.41 | |  |  |  |  |
| 5 | | GOTERM_BP_FAT | | GO:0045893~positive regulation of transcription, DNA-dependent | 1.19E-05 | 4.13E-02 | 3.51E-03 | 2.18E-02 | 72 | | 1.69 | |  |  |  |  |
|  | | ***Midnightblue module (Cor=-0.21, p-value=7x10^-4^; 1,908 CpGs in 1,441 genes)*** | | | | | | | | | | |  |  |  |  |
| 1 | | GOTERM_MF_FAT | | GO:0008134~transcription factor binding | 2.19E-07 | 2.27E-04 | 1.13E-04 | 3.47E-04 | 73 | | 1.87 | |  |  |  |  |
| 2 | | KEGG_PATHWAY | | hsa04310:Wnt signaling pathway | 2.98E-06 | 4.95E-04 | 2.47E-04 | 3.62E-03 | 29 | | 2.63 | |  |  |  |  |
| 3 | | GOTERM_MF_FAT | | GO:0016564~transcription repressor activity | 2.92E-05 | 2.98E-02 | 6.04E-03 | 4.64E-02 | 46 | | 1.92 | |  |  |  |  |
| 4 | | GOTERM_CC_FAT | | GO:0044431~Golgi apparatus part | 2.96E-05 | 1.62E-02 | 5.42E-03 | 4.32E-02 | 42 | | 1.99 | |  |  |  |  |
| 5 | | GOTERM_BP_FAT | | GO:0030182~neuron differentiation | 9.45E-05 | 2.66E-01 | 3.79E-02 | 1.72E-01 | 57 | | 1.70 | |  |  |  |  |
|  | | ***Darkolivegreen module (Cor=-0.20, p-value=1x10^-3^; 354 CpGs in 257 genes)*** | | | | | | | | | | |  |  |  |  |
| 1 | | GOTERM_BP_FAT | | GO:0006355~regulation of transcription, DNA-dependent | 9.12E-06 | 1.07E-02 | 5.36E-03 | 1.47E-02 | 46 | | 1.95 | |  |  |  |  |
| 2 | | GOTERM_CC_FAT | | GO:0031981~nuclear lumen | 8.69E-05 | 1.88E-02 | 1.88E-02 | 1.10E-01 | 33 | | 2.05 | |  |  |  |  |
|  | | ***Darkturquoise module (Cor=-0.20, p-value=1x10^-3^; 1,460 CpGs in 428 genes)*** | | | | | | | | | | |  |  |  |  |
| 1 | | GOTERM_CC_FAT | | GO:0005730~nucleolus | 7.94E-05 | 2.58E-02 | 2.58E-02 | 1.07E-01 | 31 | | 2.18 | |  |  |  |  |
| 2 | | GOTERM_BP_FAT | | GO:0016568~chromatin modification | 1.98E-03 | 9.61E-01 | 9.61E-01 | 3.28E+00 | 15 | | 2.60 | |  |  |  |  |
| 3 | | GOTERM_CC_FAT | | GO:0005743~mitochondrial inner membrane | 9.64E-03 | 9.59E-01 | 2.98E-01 | 1.23E+01 | 14 | | 2.25 | |  |  |  |  |
| 4 | | GOTERM_CC_FAT | | GO:0005768~endosome | 1.21E-02 | 9.82E-01 | 3.29E-01 | 1.52E+01 | 14 | | 2.18 | |  |  |  |  |
|  | | ***Purple module (Cor=-0.21, p-value=5x10^-4^; 3,451 CpGs in 1,815 genes)*** | | |  |  |  |  |  | |  | |  |  |  |  |
| 1 | | GOTERM_MF_FAT | | GO:0003700~transcription factor activity | 6.60E-24 | 7.79E-21 | 3.89E-21 | 1.06E-20 | 194 | | 2.06 | |  |  |  |  |
| 2 | | GOTERM_BP_FAT | | GO:0030182~neuron differentiation | 7.56E-22 | 2.82E-18 | 9.40E-19 | 1.39E-18 | 109 | | 2.65 | |  |  |  |  |
| 3 | | GOTERM_BP_FAT | | GO:0048812~neuron projection morphogenesis | 1.86E-08 | 6.95E-05 | 1.78E-06 | 3.43E-05 | 48 | | 2.40 | |  |  |  |  |
| 4 | | GOTERM_CC_FAT | | GO:0045211~postsynaptic membrane | 2.83E-08 | 1.49E-05 | 3.73E-06 | 4.11E-05 | 34 | |  | |  |  |  |  |
| 5 | | GOTERM_BP_FAT | | GO:0030901~midbrain development | 3.38E-06 | 1.25E-02 | 1.94E-04 | 6.23E-03 | 10 | | 6.66 | |  |  |  |  |
| 6 | | GOTERM_BP_FAT | | GO:0031018~endocrine pancreas development | 6.58E-06 | 2.43E-02 | 3.51E-04 | 1.21E-02 | 10 | | 6.27 | |  |  |  |  |
| 7 | | GOTERM_MF_FAT | | GO:0008066~glutamate receptor activity | 3.20E-07 | 3.77E-04 | 7.54E-05 | 5.16E-04 | 15 | | 5.00 | |  |  |  |  |
| 8 | | KEGG_PATHWAY | | hsa04514:Cell adhesion molecules (CAMs) | 1.97E-05 | 3.42E-03 | 1.71E-03 | 2.41E-02 | 29 | | 2.37 | |  |  |  |  |
| 9 | | KEGG_PATHWAY | | hsa04020:Calcium signaling pathway | 2.95E-04 | 5.00E-02 | 1.70E-02 | 3.61E-01 | 32 | | 1.96 | |  |  |  |  |
|  | | ***Darkorange module (Cor=-0.24, p-value=1x10^-4^; 1,106 CpGs in 358 genes)*** | | | | | | | | | | |  |  |  |  |
| 1 | | GOTERM_BP_FAT | | GO:0051494~negative regulation of cytoskeleton organization | 3.11E-03 | 9.90E-01 | 8.80E-01 | 5.00E+00 | 6 | | 6 | |  |  |  |  |
| 2 | | GOTERM_BP_FAT | | GO:0016568~chromatin modification | 4.28E-03 | 1.00E+00 | 8.60E-01 | 6.81E+00 | 13 | | 2.61 | |  |  |  |  |
| 3 | | GOTERM_BP_FAT | | GO:0042989~sequestering of actin monomers | 4.67E-03 | 1.00E+00 | 8.00E-01 | 7.41E+00 | 3 | | 27.5 | |  |  |  |  |
| 4 | | GOTERM_MF_FAT | | GO:0003779~actin binding | 7.43E-03 | 9.70E-01 | 9.70E-01 | 1.01E+01 | 14 | | 2.32 | |  |  |  |  |
| 5 | | GOTERM_CC_FAT | | GO:0005856~cytoskeleton | 8.99E-03 | 9.17E-01 | 3.92E-01 | 1.12E+01 | 37 | | 1.53 | |  |  |  |  |
| 6 | | GOTERM_CC_FAT | | GO:0005740~mitochondrial envelope | 1.53E-02 | 9.86E-01 | 4.13E-01 | 1.84E+01 | 15 | | 2.04 | |  |  |  |  |
| 7 | | GOTERM_CC_FAT | | GO:0005783~endoplasmic reticulum | 1.66E-02 | 9.90E-01 | 3.70E-01 | 1.98E+01 | 27 | | 1.6 | |  |  |  |  |
|  | | ***Steelblue module (Cor=-0.23, p-value=2x10^-4^; 546 CpGs in 214 genes)*** | | |  |  |  |  |  | |  | |  |  |  |  |
| 1 | | GOTERM_MF_FAT | | GO:0016790~thiolester hydrolase activity | 2.22E-02 | 9.99E-01 | 9.99E-01 | 2.62E+01 | 5 | | 4.65 | |  |  |  |  |
| 2 | | GOTERM_BP_FAT | | GO:0031175~neuron projection development | 2.48E-02 | 1.00E+00 | 1.00E+00 | 3.25E+01 | 8 | | 2.78 | |  |  |  |  |
| 3 | | KEGG_PATHWAY | | hsa04722:Neurotrophin signaling pathway | 3.94E-02 | 9.71E-01 | 6.92E-01 | 3.53E+01 | 5 | | 3.80 | |  |  |  |  |
| 4 | | GOTERM_MF_FAT | | GO:0030528~transcription regulator activity | 4.18E-02 | 1.00E+00 | 9.90E-01 | 4.39E+01 | 24 | | 1.50 | |  |  |  |  |
| 5 | | GOTERM_MF_FAT | | GO:0004221~ubiquitin thiolesterase activity | 4.40E-02 | 1.00E+00 | 9.74E-01 | 4.56E+01 | 4 | | 5.05 | |  |  |  |  |
| 6 | | GOTERM_MF_FAT | | GO:0000287~magnesium ion binding | 4.81E-02 | 1.00E+00 | 9.59E-01 | 4.86E+01 | 10 | | 2.10 | |  |  |  |  |
| GOTERM_BP: Biological Process, GOTERM_MF: Molecular Function, GOTERM_CC: Cellular Component, KEGG: Kyoto Encyclopedia of Genes and Genomes. | | | | | | | | | | | | | | |  |  |

| **Table S8. Summary of EWAS results in blood and saliva.** Correlations between PD and DNA methylation levels at 450k CpG sites in blood and saliva. | | | | | | | | | |
| --- | --- | --- | --- | --- | --- | --- | --- | --- | --- |
| **Models** | **Subjects (N)** | **Min Cor** | **Max Cor** | **Min Zscore** | **Max Zscore** | **Min Pvalue** | **Number of CpGs with p-value < 10^-7^ (N)** | **Proportion of hypomethylated CpGs in CpGs with p-value < 10^-7^ (%)** | **Proportion of hypomethylated CpGs in the top 2,000 most significant PD-associated CpGs (%)** |
| **DNA methylation level in blood** |  |  |  |  |  |  |  |  |  |
| **Caucasian-only** |  |  |  |  |  |  |  |  |  |
| Crude | 508 | -0.30 | 0.28 | -6.89 | 6.37 | 8.00E-12 | 386 | 81% | 86% |
| Adjusted for age, gender | 508 | -0.30 | 0.28 | -6.55 | 6.12 | 8.34E-11 | 82 | 77% | 80% |
| Adjusted for age, gender, blood cell composition | 508 | -0.24 | 0.24 | -5.26 | 5.25 | 1.66E-07 | 0 | - | 31% |
| Adjusted for age, gender, blood cell composition, smoking | 508 | -0.24 | 0.24 | -5.17 | 5.22 | 2.08E-07 | 0 | - | 27% |
| **Hispanic-only** |  |  |  |  |  |  |  |  |  |
| Crude | 64 | -0.55 | 0.48 | -4.92 | 4.13 | 2.03E-06 | 0 | - | 96% |
| Adjusted for age, gender | 64 | -0.55 | 0.48 | -4.73 | 4.01 | 4.84E-06 | 0 | - | 90% |
| Adjusted for age, gender, blood cell composition | 64 | -0.52 | 0.49 | -4.38 | 4.06 | 2.13E-05 | 0 | - | 75% |
| Adjusted for age, gender, blood cell composition, smoking | 64 | -0.53 | 0.49 | -4.51 | 4.09 | 1.22E-05 | 0 | - | 75% |
| **Caucasian and Hispanic  (meta-analysis)** |  |  |  |  |  |  |  |  |  |
| Adjusted for age, gender | 572 | - | - | -6.58 | 5.56 | 4.67E-11 | 116 | 95% | 94% |
| Adjusted for age, gender, blood cell composition | 572 | - | - | -4.69 | 4.82 | 1.45E-06 | 0 | - | 52% |
| Adjusted for age, gender, blood cell composition, smoking | 572 | - | - | -4.65 | 4.65 | 3.30E-06 | 0 | - | 51% |
| **DNA methylation level in saliva** |  |  |  |  |  |  |  |  |  |
| **Caucasian and Hispanic  (pooled analysis)** |  |  |  |  |  |  |  |  |  |
| Adjusted for age, gender, race | 259 | -0.35 | 0.32 | -5.79 | 5.34 | 1.05E-08 | 5 | 100% | 88% |
| Adjusted for age, gender, race, smoking | 259 | -0.34 | 0.32 | -5.68 | 5.28 | 1.97E-08 | 3 | 100% | 86% |
| Cor: Correlation coefficient from student t-test; Z score: Stouffer's test Z score from meta-analysis. | | | | | | | | | |

| **Table S9. Correlations between each thistle module CpG and PD status, age, and male, respectively.** The Pearson’s correlation coefficients and the corresponding p-values are shown. | | | | | | | | | | | | | | |
| --- | --- | --- | --- | --- | --- | --- | --- | --- | --- | --- | --- | --- | --- | --- |
|  | **CpG** | **Gene** | **Chr.** | **Position (bp)** | **Relation to UCSC CpG Island** | **Gene region** | **SNPs** | **SNPs_10** | **cor.PD** | **p-value.**  **PD** | **cor.Age** | **p-value.**  **Age** | **cor.Male** | **p-value.**  **Male** |
| 1 | cg13451886 | SLFN5 | 17 | 33568791 | N_Shore | TSS1500 |  |  | 0.26 | 2.63E-09 | 0.28 | 2.51E-10 | 0.20 | 4.04E-06 |
| 2 | cg25893560 | KCNJ1 | 11 | 128710122 |  | Body |  |  | 0.26 | 2.69E-09 | 0.25 | 1.04E-08 | 0.19 | 1.91E-05 |
| 3 | cg07679948 | DGKA | 12 | 56329641 | Island | 5'UTR |  |  | 0.26 | 2.73E-09 | 0.26 | 2.01E-09 | 0.20 | 3.30E-06 |
| 4 | cg15997393 | MAD1L1 | 7 | 1961869 |  | Body | rs73050128 | 0.26 | | 4.80E-09 | 0.33 | 4.06E-14 | 0.22 | 3.67E-07 |
| 5 | cg20937934 | LAMA3 | 18 | 21452788 |  | TSS200 | rs28413178 | 0.25 | | 1.03E-08 | 0.36 | 4.45E-17 | 0.22 | 6.97E-07 |
| 6 | cg17346246 | GPR81 | 12 | 123214864 |  | 1stExon |  |  | 0.25 | 1.21E-08 | 0.26 | 1.55E-09 | 0.21 | 2.48E-06 |
| 7 | cg00895196 | FLJ22536 | 6 | 22147182 |  | Body |  |  | 0.25 | 1.54E-08 | 0.29 | 2.29E-11 | 0.21 | 1.11E-06 |
| 8 | cg24199463 | ATP11A | 13 | 113407479 | N_Shelf | Body |  |  | 0.24 | 7.63E-08 | 0.29 | 3.76E-11 | 0.18 | 4.50E-05 |
| 9 | cg02963266 | BCL11B | 14 | 99681710 |  | Body |  |  | 0.24 | 8.02E-08 | 0.32 | 8.06E-14 | 0.20 | 8.33E-06 |
| 10 | cg01154505 | FBLN7 | 2 | 112940409 | S_Shore | Body |  |  | 0.23 | 9.79E-08 | 0.26 | 4.40E-09 | 0.21 | 1.15E-06 |
| 11 | cg02387618 | TCF7 | 5 | 133453480 | S_Shelf | Body |  |  | 0.21 | 2.52E-06 | 0.28 | 1.35E-10 | 0.25 | 1.08E-08 |
| 12 | cg24662823 | ETS1 | 11 | 128350635 |  | Body | rs7108562 | 0.20 | | 4.30E-06 | 0.23 | 1.13E-07 | 0.20 | 4.05E-06 |
| 13 | cg25325005 | PLEC1 | 8 | 145012748 | N_Shelf | Body | rs7823393 | rs7844225 | 0.20 | 4.41E-06 | 0.14 | 1.44E-03 | 0.23 | 1.16E-07 |
| 14 | cg15227911 | CHD3 | 17 | 7792059 | S_Shelf | Body |  |  | 0.19 | 1.20E-05 | 0.26 | 2.10E-09 | 0.23 | 2.85E-07 |
| 15 | cg01974478 | FOXK1 | 7 | 4779312 |  | Body | rs57323934 | 0.19 | | 1.60E-05 | 0.21 | 2.18E-06 | 0.21 | 2.35E-06 |
| 16 | cg20558320 |  | 2 | 235210313 |  |  |  |  | 0.19 | 1.95E-05 | 0.22 | 5.29E-07 | 0.21 | 1.11E-06 |
| 17 | cg05017994 |  | 5 | 964562 |  |  |  |  | 0.19 | 2.50E-05 | 0.32 | 8.21E-14 | 0.28 | 1.15E-10 |
| 18 | cg23953820 | DDR1 | 6 | 30851051 | N_Shore | TSS1500 |  |  | 0.18 | 3.07E-05 | 0.32 | 1.01E-13 | 0.28 | 1.45E-10 |
| 19 | cg07713946 | LIMK2 | 22 | 31675144 | S_Shelf | 3'UTR |  |  | 0.18 | 3.09E-05 | 0.23 | 1.75E-07 | 0.24 | 6.42E-08 |
| 20 | cg26347170 | CHMP4B | 20 | 32441424 |  | 3'UTR |  |  | 0.18 | 3.23E-05 | 0.31 | 1.61E-12 | 0.24 | 7.81E-08 |
| 21 | cg06329392 |  | 13 | 40762435 |  |  |  |  | 0.18 | 3.86E-05 | 0.27 | 4.54E-10 | 0.24 | 5.51E-08 |
| 22 | cg21565496 |  | 13 | 40762150 |  |  |  |  | 0.18 | 4.18E-05 | 0.27 | 1.02E-09 | 0.28 | 1.31E-10 |
| 23 | cg17824906 | FBXL14 | 12 | 1700011 | N_Shelf | Body |  |  | 0.18 | 4.18E-05 | 0.20 | 6.29E-06 | 0.24 | 7.24E-08 |
| 24 | cg13937905 | RARG | 12 | 53612551 | N_Shore | Body |  |  | 0.18 | 4.93E-05 | 0.26 | 1.51E-09 | 0.18 | 4.06E-05 |
| 25 | cg04347414 | PRKCZ | 1 | 2084519 | S_Shore | Body |  |  | 0.17 | 8.43E-05 | 0.20 | 8.08E-06 | 0.12 | 6.97E-03 |
| 26 | cg21900799 | PLEC1 | 8 | 145004388 | S_Shore | Body |  |  | 0.17 | 8.47E-05 | 0.20 | 6.82E-06 | 0.13 | 4.06E-03 |
| 27 | cg25226014 | CXCR6 | 3 | 45984942 |  | TSS200 |  |  | 0.17 | 8.58E-05 | 0.19 | 1.03E-05 | 0.21 | 2.99E-06 |
| 28 | cg22488891 |  | 13 | 40762452 |  |  |  |  | 0.17 | 8.70E-05 | 0.26 | 4.72E-09 | 0.22 | 7.82E-07 |
| 29 | cg23479730 | BCL11B | 14 | 99681757 |  | Body |  |  | 0.17 | 1.01E-04 | 0.32 | 1.68E-13 | 0.14 | 1.80E-03 |
| 30 | cg00017826 |  | 2 | 30644955 |  |  |  |  | 0.17 | 1.16E-04 | 0.23 | 9.92E-08 | 0.23 | 1.34E-07 |
| 31 | cg12327866 |  | 10 | 1939663 |  |  |  |  | 0.17 | 1.45E-04 | 0.11 | 1.47E-02 | 0.16 | 2.57E-04 |
| 32 | cg14522718 | SLC25A25 | 9 | 130868874 |  | Body |  |  | 0.17 | 1.80E-04 | 0.28 | 9.46E-11 | 0.25 | 5.58E-09 |
| 33 | cg14024328 | PCGF3 | 4 | 719362 | S_Shelf | 5'UTR |  |  | 0.17 | 1.85E-04 | 0.09 | 4.66E-02 | 0.16 | 2.81E-04 |
| 34 | cg03641640 | RASA3 | 13 | 114776416 | S_Shore | Body |  |  | 0.16 | 2.14E-04 | 0.19 | 1.01E-05 | 0.00 | 9.45E-01 |
| 35 | cg15562346 |  | 2 | 239403295 |  |  |  |  | 0.16 | 2.42E-04 | 0.32 | 9.11E-14 | 0.14 | 1.59E-03 |
| 36 | cg21524538 | SDCBP2 | 20 | 1310846 |  | TSS1500 | rs6041464 | 0.16 | | 3.37E-04 | 0.14 | 2.10E-03 | 0.16 | 1.96E-04 |
| 37 | cg05345285 | DOT1L | 19 | 2215718 | N_Shore | Body |  |  | 0.16 | 4.05E-04 | 0.15 | 6.36E-04 | 0.19 | 1.72E-05 |
| 38 | cg08469255 | DDR1 | 6 | 30851069 | N_Shore | TSS1500 |  |  | 0.16 | 4.06E-04 | 0.30 | 7.15E-12 | 0.26 | 2.05E-09 |
| 39 | cg03318904 | MAP3K7IP1 | 22 | 39801522 |  | Body |  |  | 0.15 | 5.03E-04 | 0.22 | 4.29E-07 | 0.18 | 4.51E-05 |
| 40 | cg08280368 | TTC9 | 14 | 71110536 | S_Shore | Body | rs74063312 | 0.15 | | 5.13E-04 | 0.19 | 2.46E-05 | 0.04 | 4.21E-01 |
| 41 | cg07019638 | EHD1 | 11 | 64635774 |  | Body |  |  | 0.15 | 5.60E-04 | 0.18 | 4.97E-05 | 0.18 | 6.05E-05 |
| 42 | cg13496119 |  | 14 | 98341006 |  |  |  |  | 0.15 | 7.59E-04 | 0.18 | 2.74E-05 | 0.17 | 1.72E-04 |
| 43 | cg08362785 | MKL1 | 22 | 40814878 | Island | Body |  |  | 0.15 | 9.51E-04 | 0.37 | 1.87E-17 | 0.26 | 3.41E-09 |
| 44 | cg00144180 | HDAC4 | 2 | 240294362 |  | 5'UTR |  |  | 0.14 | 1.06E-03 | 0.45 | 4.12E-26 | 0.23 | 9.90E-08 |
| 45 | cg15275758 |  | 12 | 133412815 | Island |  | rs36046850 | rs74473743 | 0.14 | 1.09E-03 | 0.20 | 5.62E-06 | 0.16 | 4.18E-04 |
| 46 | cg18181923 | BCL11B | 14 | 99682269 |  | Body |  |  | 0.14 | 1.10E-03 | 0.12 | 8.08E-03 | 0.07 | 1.33E-01 |
| 47 | cg26003388 | TMC8 | 17 | 76129533 | S_Shore | Body |  |  | 0.14 | 1.49E-03 | 0.09 | 4.33E-02 | 0.17 | 8.90E-05 |
| 48 | cg17864041 |  | 15 | 31175309 | Island |  |  |  | 0.14 | 1.97E-03 | 0.20 | 8.27E-06 | 0.17 | 1.33E-04 |
| 49 | cg18758433 | RPTOR | 17 | 78623601 |  | Body | rs59316918 | 0.14 | | 2.29E-03 | 0.20 | 3.65E-06 | 0.19 | 1.02E-05 |
| 50 | cg17850055 |  | 2 | 239403239 |  |  |  |  | 0.13 | 2.50E-03 | 0.25 | 1.87E-08 | 0.23 | 1.09E-07 |
| 51 | cg00459992 |  | 7 | 158819232 | S_Shore |  |  |  | 0.13 | 2.56E-03 | 0.24 | 2.43E-08 | 0.09 | 4.95E-02 |
| 52 | cg03596635 | ABTB1 | 3 | 127393517 | S_Shore | Body | rs72967683 | rs62264122 | 0.13 | 2.60E-03 | 0.21 | 2.34E-06 | 0.17 | 1.32E-04 |
| 53 | cg01083549 | ACSF3 | 16 | 89168371 | S_Shore | Body |  |  | 0.13 | 2.77E-03 | 0.21 | 1.32E-06 | 0.10 | 3.07E-02 |
| 54 | cg18021785 | EXOC4 | 7 | 133168802 |  | 3'UTR |  |  | 0.13 | 3.28E-03 | 0.16 | 2.49E-04 | 0.13 | 3.49E-03 |
| 55 | cg15564619 | SKI | 1 | 2163437 | S_Shelf | Body |  |  | 0.13 | 3.33E-03 | 0.17 | 1.30E-04 | 0.09 | 4.15E-02 |
| 56 | cg15001636 | SMARCA4 | 19 | 11144178 | Island | Body |  |  | 0.13 | 4.33E-03 | 0.17 | 1.29E-04 | 0.20 | 6.15E-06 |
| 57 | cg00791190 |  | 22 | 50223573 | S_Shore |  |  | rs79032976 | 0.12 | 5.95E-03 | 0.12 | 6.69E-03 | 0.19 | 1.27E-05 |
| 58 | cg04892187 | PLEC1 | 8 | 145040629 |  | Body |  |  | 0.11 | 1.01E-02 | 0.18 | 5.79E-05 | 0.17 | 9.98E-05 |
| 59 | cg20702527 | HDAC4 | 2 | 240086087 |  | Body |  |  | 0.11 | 1.13E-02 | 0.21 | 2.70E-06 | 0.07 | 1.32E-01 |
| 60 | cg19961800 | SYT16 | 14 | 62550978 |  | Body |  |  | 0.11 | 1.27E-02 | 0.23 | 1.22E-07 | 0.20 | 5.19E-06 |
| 61 | cg08297616 |  | 16 | 85433718 |  |  | rs12929971 | 0.10 | | 1.88E-02 | 0.15 | 5.36E-04 | 0.27 | 4.02E-10 |
| 62 | cg12297231 | PUM1 | 1 | 31531229 |  | Body |  |  | 0.10 | 2.68E-02 | 0.26 | 5.06E-09 | 0.12 | 6.75E-03 |
| 63 | cg21104449 | PAK2 | 3 | 196485278 |  | 5'UTR |  |  | 0.10 | 3.14E-02 | 0.23 | 1.75E-07 | 0.18 | 4.02E-05 |
| 64 | cg18302225 |  | 5 | 55776401 | N_Shore |  |  |  | 0.09 | 3.31E-02 | 0.10 | 3.15E-02 | 0.09 | 3.40E-02 |
| 65 | cg09177577 | BIN3 | 8 | 22503577 |  | Body | rs35083320 | 0.09 | | 4.38E-02 | 0.18 | 3.59E-05 | 0.18 | 4.92E-05 |
| 66 | cg23698124 | SMG6 | 17 | 2154177 |  | Body |  |  | 0.09 | 4.82E-02 | 0.27 | 7.03E-10 | 0.12 | 4.99E-03 |
| 67 | cg22454769 | FHL2 | 2 | 106015767 | Island | TSS200 |  |  | 0.09 | 5.21E-02 | 0.61 | 6.49E-54 | 0.03 | 4.55E-01 |
| 68 | cg19743522 | DTX1 | 12 | 113495566 | S_Shore | TSS200 |  |  | 0.09 | 5.32E-02 | 0.03 | 4.75E-01 | 0.02 | 7.28E-01 |
| 69 | cg15605704 | AJAP1 | 1 | 4770676 | N_Shore | Body |  |  | 0.08 | 6.88E-02 | 0.10 | 1.95E-02 | -0.02 | 7.28E-01 |
| 70 | cg11011533 | ITGAE | 17 | 3674649 |  | Body |  |  | 0.08 | 7.46E-02 | 0.38 | 6.53E-19 | 0.14 | 1.27E-03 |
| 71 | cg15730481 | GPER | 7 | 1126318 | N_Shore | TSS200 |  |  | 0.08 | 8.51E-02 | 0.11 | 1.42E-02 | 0.19 | 1.13E-05 |
| 72 | cg08972190 | MAD1L1 | 7 | 2138995 |  | Body |  | rs73034464 | 0.07 | 1.06E-01 | 0.15 | 6.07E-04 | 0.14 | 1.70E-03 |
| 73 | cg01832672 | VPS37B | 12 | 123358583 |  | Body |  |  | 0.07 | 1.21E-01 | 0.15 | 5.53E-04 | 0.13 | 2.58E-03 |
| 74 | cg02167713 |  | 17 | 79630115 | N_Shelf |  | rs12325815 | 0.06 | | 1.62E-01 | 0.21 | 2.97E-06 | 0.13 | 3.77E-03 |
| 75 | cg17939040 | ACACA | 17 | 35503956 |  | Body |  |  | 0.05 | 2.24E-01 | 0.15 | 8.66E-04 | 0.19 | 1.21E-05 |
| 76 | cg19893929 |  | 2 | 16105823 |  |  |  |  | 0.05 | 2.94E-01 | 0.17 | 1.72E-04 | 0.24 | 5.11E-08 |
| 77 | cg09084391 | AHRR | 5 | 346247 | S_Shore | Body | rs78566649 | 0.05 | | 3.01E-01 | 0.13 | 2.70E-03 | 0.09 | 3.76E-02 |
| 78 | cg09245989 | PANK4 | 1 | 2454811 | S_Shore | Body |  |  | 0.04 | 3.61E-01 | 0.13 | 4.40E-03 | 0.06 | 1.49E-01 |
| 79 | cg23038277 | ITPKB | 1 | 226833134 | S_Shelf | Body |  |  | 0.04 | 3.71E-01 | 0.15 | 9.04E-04 | 0.04 | 3.36E-01 |
| 80 | cg01578875 | ZNF827 | 4 | 146804010 |  | Body |  |  | 0.04 | 4.26E-01 | 0.23 | 1.00E-07 | 0.21 | 2.66E-06 |
| 81 | cg12682323 |  | 10 | 132883127 |  |  |  | rs2397745 | 0.03 | 5.61E-01 | 0.11 | 1.77E-02 | -0.05 | 2.45E-01 |
| 82 | cg24118521 | HTR2A | 13 | 47472330 |  | TSS1500 |  |  | 0.02 | 6.85E-01 | 0.17 | 1.24E-04 | 0.11 | 1.51E-02 |
| 83 | cg05647602 | BID | 22 | 18258664 | S_Shore | TSS1500 | rs8190252 | 0.01 | | 7.69E-01 | 0.08 | 7.72E-02 | 0.14 | 1.05E-03 |
| 84 | cg22614203 | HPCAL1 | 2 | 10517361 |  | 5'UTR |  |  | -0.01 | 8.48E-01 | 0.04 | 3.23E-01 | 0.19 | 1.85E-05 |
| Chr.: Chromosome, bp: base pair, TSS: transcription start site, TSS1500: within 1500 bps of a TSS, TSS200: within 200 bps of a TSS, UTR: untranslated region, SNPs: listing dbSNP entries within a probe, SNPs_10: listing dbSNP entries within 10 bp of the CpG site. | | | | | | | | | | | | | | |

**Table S10. Methylation levels of the 3 most significant PD-associated CpGs in blood and saliva.** The median, min, max methylation levels of the 3 most significant PD-associated CpGs in blood and saliva.

| **(a) List of 3 top hits in 508 PEG1 subjects of European ancestry adjusting for age and gender** | | |
| --- | --- | --- |
|  | **PD (N=289)** | **Controls (N=219)** |
| cg02489202, LARS2 |  |  |
| β-value | 0.34 (0.18, 0.53) | 0.37 (0.22, 0.59) |
| Residuals* | -0.01 (-0.17, 0.17) | 0.02 (-0.12, 0.24) |
| cg04772575, ABCB9 |  |  |
| β-value | 0.43 (0.09, 0.71) | 0.46 (0.29, 0.57) |
| Residuals* | 0.01 (-0.35, 0.25) | 0.02 (-0.13, 0.17) |
| cg11334709, C1orf200 |  |  |
| β-value | 0.40 (0.20, 0.50) | 0.42 (0.27, 0.57) |
| Residuals* | 0.00 (-0.20, 0.09) | 0.01 (-0.13, 0.18) |
| *Age and gender adjusted | | |
| **(b) List of 3 top hits in 508 PEG1 subjects of European ancestry adjusting for age, gender, and blood cell composition** | | |
|  | **PD (N=289)** | **Controls (N=219)** |
| cg05001044, MIR1977 |  |  |
| β-value | 0.31 (0.09, 0.45) | 0.35 (0.12, 0.44) |
| Residuals* | 0.00 (-0.23, 0.18) | 0.03 (-0.19, 0.12) |
| cg27211284 |  |  |
| β-value | 0.27 (0.15, 0.74) | 0.26 (0.14, 0.42) |
| Residuals* | 0.00 (-0.11, 0.35) | -0.01 (-0.17, 0.10) |
| cg05853632, RNF39 |  |  |
| β-value | 0.05 (0.02, 0.15) | 0.04 (0.01, 0.10) |
| Residuals* | 0.00 (-0.04, 0.10) | -0.01 (-0.03, 0.06) |
| *Age, gender, and blood cell composition adjusted | |  |
| **(c) List of 3 top hits in 259 PEG2 subjects adjusting for age, gender, and race** | | |
|  | **PD (N=128)** | **Controls (N=131)** |
| cg15133963, FTHL3 |  |  |
| β-value | 0.72 (0.61, 0.84) | 0.74 (0.67, 0.84) |
| Residuals* | -0.01 (-0.12, 0.11) | 0.01 (-0.06, 0.11) |
| cg01820192, C21orf125 |  |  |
| β-value | 0.51 (0.38, 0.67) | 0.55 (0.42, 0.69) |
| Residuals* | -0.02 (-0.14, 0.13) | 0.02 (-0.10, 0.18) |
| cg22275276 |  |  |
| β-value | 0.76 (0.66, 0.88) | 0.78 (0.70, 0.92) |
| Residuals* | -0.01 (-0.11, 0.10) | 0.01 (-0.07, 0.15) |
| *Age, gender and race adjusted | | |

**Table S11. Logistic regression results of the association between CpGs and PD status.**

| (a) 82 blood based top hits (p-value < 10^-7^) without adjustment for blood cell compositions from **Table 1** | | | | | | | | | | | | | | | | | | | | | | | | | | | | | | |  |  |  |
| --- | --- | --- | --- | --- | --- | --- | --- | --- | --- | --- | --- | --- | --- | --- | --- | --- | --- | --- | --- | --- | --- | --- | --- | --- | --- | --- | --- | --- | --- | --- | --- | --- | --- |
|  | | | | | | **CpG** | | | **Gene** | | **Chr.** | | **Position (bp)** | | | **Relation to UCSC CpG Island** | **Gene region** | | **SNPs** | | | **SNPs_10** | | | **OR** | | | **95% CI** | **logistic  p-value** | |  |  |  |
| 1 | | | | | | cg02489202 | | | LARS2 | | 3 | | 45505334 | | |  | Body | |  | | |  | | | 0.89 | | | (0.86,0.92) | 4.48E-10 | |  |  |  |
| 2 | | | | | | cg04772575 | | | ABCB9 | | 12 | | 123431865 | | |  | Body | | rs73408862 | | |  | | | 0.88 | | | (0.85,0.92) | 6.47E-09 | |  |  |  |
| 3 | | | | | | cg11334709 | | | C1orf200 | | 1 | | 9716019 | | | S_Shelf | TSS1500 | |  | | |  | | | 0.86 | | | (0.82,0.90) | 2.29E-09 | |  |  |  |
| 4 | | | | | | cg17491368 | | |  | | 1 | | 211779938 | | |  |  | |  | | |  | | | 0.91 | | | (0.89,0.94) | 2.72E-08 | |  |  |  |
| 5 | | | | | | cg16240816 | | |  | | 2 | | 65861662 | | |  |  | | rs80084148 | | |  | | | 1.10 | | | (1.07,1.14) | 7.77E-09 | |  |  |  |
| 6 | | | | | | cg19879906 | | |  | | 19 | | 16392219 | | | N_Shelf |  | |  | | |  | | | 0.89 | | | (0.85,0.92) | 6.16E-09 | |  |  |  |
| 7 | | | | | | cg08704934 | | | C3orf21 | | 3 | | 194826585 | | |  | Body | |  | | | rs6799614 | | | 0.91 | | | (0.88,0.94) | 8.44E-09 | |  |  |  |
| 8 | | | | | | cg09993145 | | | RUNX3 | | 1 | | 25291905 | | |  | TSS1500 | |  | | |  | | | 1.05 | | | (1.03,1.07) | 5.25E-08 | |  |  |  |
| 9 | | | | | | cg01152726 | | | LAMA3 | | 18 | | 21452844 | | |  | TSS200 | |  | | |  | | | 1.20 | | | (1.12,1.28) | 7.74E-08 | |  |  |  |
| 10 | | | | | | cg09032544 | | | CD247 | | 1 | | 167487295 | | |  | Body | |  | | |  | | | 1.09 | | | (1.06,1.12) | 1.45E-08 | |  |  |  |
| 11 | | | | | | cg10752406 | | | AZU1 | | 19 | | 827776 | | |  | TSS200 | |  | | |  | | | 0.90 | | | (0.87,0.93) | 3.03E-08 | |  |  |  |
| 12 | | | | | | cg26341831 | | | TMEM63A | | 1 | | 226036279 | | |  | Body | |  | | |  | | | 1.08 | | | (1.05,1.10) | 4.79E-08 | |  |  |  |
| 13 | | | | | | cg01213231 | | | ITGA5 | | 12 | | 54806218 | | |  | Body | |  | | |  | | | 0.89 | | | (0.85,0.93) | 3.06E-08 | |  |  |  |
| 14 | | | | | | cg16643542 | | | AZU1 | | 19 | | 827843 | | |  | 1stExon | | rs34124897 | | |  | | | 0.91 | | | (0.87,0.94) | 3.92E-08 | |  |  |  |
| 15 | | | | | | cg21577598 | | | CCDC57 | | 17 | | 80084751 | | | N_Shore | Body | |  | | |  | | | 1.08 | | | (1.05,1.11) | 5.74E-08 | |  |  |  |
| 16 | | | | | | cg26793227 | | | EPHA2 | | 1 | | 16483658 | | | S_Shore | TSS1500 | | rs57602506 | | |  | | | 0.91 | | | (0.88,0.94) | 1.33E-08 | |  |  |  |
| 17 | | | | | | cg16270399 | | | LOC284276 | | 18 | | 74257894 | | |  | Body | |  | | |  | | | 0.93 | | | (0.91,0.95) | 8.37E-09 | |  |  |  |
| 18 | | | | | | cg22358291 | | | WDR1 | | 4 | | 10101553 | | |  | Body | |  | | |  | | | 1.10 | | | (1.06,1.13) | 2.75E-08 | |  |  |  |
| 19 | | | | | | cg26681770 | | | PMEPA1 | | 20 | | 56247302 | | | Island | 5'UTR | | rs36008751 | | |  | | | 0.93 | | | (0.91,0.95) | 3.16E-09 | |  |  |  |
| 20 | | | | | | cg01657758 | | | SORL1 | | 11 | | 121349740 | | |  | Body | |  | | |  | | | 0.89 | | | (0.86,0.93) | 2.74E-08 | |  |  |  |
| 21 | | | | | | cg24168413 | | | FXYD1 | | 19 | | 35630388 | | | N_Shore | 5'UTR | | rs77395635 | | |  | | | 0.86 | | | (0.81,0.91) | 6.02E-08 | |  |  |  |
| 22 | | | | | | cg26474124 | | |  | | 2 | | 70368457 | | | N_Shore |  | | rs11685382 | | |  | | | 0.92 | | | (0.89,0.94) | 2.60E-08 | |  |  |  |
| 23 | | | | | | cg12792363 | | | LGALS12 | | 11 | | 63274030 | | |  | Body | |  | | |  | | | 0.92 | | | (0.89,0.95) | 5.05E-08 | |  |  |  |
| 24 | | | | | | cg12500949 | | |  | | 2 | | 88357920 | | | S_Shelf |  | |  | | |  | | | 1.12 | | | (1.08,1.17) | 7.67E-08 | |  |  |  |
| 25 | | | | | | cg16580197 | | |  | | 8 | | 67841925 | | | S_Shelf |  | |  | | |  | | | 0.92 | | | (0.90,0.95) | 2.08E-08 | |  |  |  |
| 26 | | | | | | cg19709355 | | | RARA | | 17 | | 38504102 | | | S_Shelf | Body | |  | | |  | | | 0.82 | | | (0.77,0.88) | 3.25E-08 | |  |  |  |
| 27 | | | | | | cg24339704 | | | GNG7 | | 19 | | 2529022 | | | S_Shelf | 5'UTR | | rs740054 | | |  | | | 0.93 | | | (0.91,0.95) | 1.86E-08 | |  |  |  |
| 28 | | | | | | cg02600394 | | | TXK | | 4 | | 48136234 | | |  | 5'UTR | |  | | |  | | | 1.10 | | | (1.06,1.14) | 6.46E-08 | |  |  |  |
| 29 | | | | | | cg23207054 | | | CSF3 | | 17 | | 38171530 | | |  | TSS200 | |  | | |  | | | 0.90 | | | (0.87,0.93) | 3.52E-08 | |  |  |  |
| 30 | | | | | | cg14659511 | | | DOCK9 | | 13 | | 99668433 | | |  | Body | |  | | |  | | | 1.11 | | | (1.06,1.15) | 2.02E-07 | |  |  |  |
| 31 | | | | | | cg19081101 | | | CHI3L1 | | 1 | | 203156625 | | |  | TSS1500 | |  | | |  | | | 0.94 | | | (0.92,0.96) | 5.51E-08 | |  |  |  |
| 32 | | | | | | cg20357538 | | | CHSY1 | | 15 | | 101777761 | | |  | Body | |  | | | rs11855006 | | | 0.93 | | | (0.90,0.95) | 3.65E-08 | |  |  |  |
| 33 | | | | | | cg26279840 | | | IKBKG | | X | | 153770418 | | |  | TSS200 | |  | | |  | | | 0.91 | | | (0.88,0.94) | 1.89E-07 | |  |  |  |
| 34 | | | | | | cg17879101 | | | FAM53B | | 10 | | 126329354 | | |  | Body | |  | | |  | | | 0.93 | | | (0.90,0.95) | 6.65E-08 | |  |  |  |
| 35 | | | | | | cg27640064 | | | FOXK1 | | 7 | | 4752983 | | |  | Body | |  | | | rs77026339 | | | 0.91 | | | (0.87,0.94) | 5.80E-08 | |  |  |  |
| 36 | | | | | | cg02505177 | | | MGEA5 | | 10 | | 103574626 | | | N_Shelf | Body | |  | | |  | | | 1.08 | | | (1.05,1.12) | 1.21E-07 | |  |  |  |
| 37 | | | | | | cg23189692 | | | EIF4G1 | | 3 | | 184050393 | | | N_Shelf | Body | |  | | |  | | | 0.94 | | | (0.92,0.96) | 4.46E-08 | |  |  |  |
| 38 | | | | | | cg15209885 | | | CBX2 | | 17 | | 77753199 | | | S_Shore | Body | | rs72231015 | | |  | | | 0.91 | | | (0.87,0.94) | 6.41E-08 | |  |  |  |
| 39 | | | | | | cg19011001 | | | ITPK1 | | 14 | | 93539613 | | |  | Body | |  | | |  | | | 0.92 | | | (0.89,0.95) | 1.06E-07 | |  |  |  |
| 40 | | | | | | cg17984638 | | | TXK | | 4 | | 48136452 | | |  | TSS200 | |  | | |  | | | 1.10 | | | (1.06,1.14) | 9.51E-08 | |  |  |  |
| 41 | | | | | | cg17173442 | | | RFXANK | | 19 | | 19305340 | | | S_Shore | Body | |  | | |  | | | 0.89 | | | (0.85,0.93) | 5.78E-08 | |  |  |  |
| 42 | | | | | | cg26489413 | | | AMPD3 | | 11 | | 10476976 | | |  | 1stExon | |  | | |  | | | 0.94 | | | (0.91,0.96) | 2.35E-07 | |  |  |  |
| 43 | | | | | | cg20720686 | | | POR | | 7 | | 75582881 | | |  | 5'UTR | | rs41295375 | | |  | | | 0.94 | | | (0.92,0.96) | 1.09E-07 | |  |  |  |
| 44 | | | | | | cg08069287 | | |  | | 11 | | 72868833 | | |  |  | |  | | |  | | | 0.93 | | | (0.90,0.95) | 1.31E-07 | |  |  |  |
| 45 | | | | | | cg26963632 | | |  | | 16 | | 85558148 | | |  |  | |  | | |  | | | 0.91 | | | (0.87,0.94) | 1.35E-07 | |  |  |  |
| 46 | | | | | | cg14023999 | | |  | | 15 | | 90543224 | | | N_Shore |  | |  | | |  | | | 0.93 | | | (0.90,0.95) | 1.33E-07 | |  |  |  |
| 47 | | | | | | cg02861056 | | | PLEK | | 2 | | 68592345 | | |  | 1stExon | |  | | |  | | | 0.89 | | | (0.86,0.93) | 1.57E-08 | |  |  |  |
| 48 | | | | | | cg24185397 | | |  | | 17 | | 25659609 | | | N_Shore |  | |  | | |  | | | 0.84 | | | (0.78,0.90) | 4.94E-07 | |  |  |  |
| 49 | | | | | | cg13060970 | | | PLEK | | 2 | | 68592349 | | |  | 1stExon | |  | | |  | | | 0.90 | | | (0.87,0.93) | 1.02E-08 | |  |  |  |
| 50 | | | | | | cg21252105 | | |  | | 9 | | 139459307 | | |  |  | |  | | |  | | | 0.91 | | | (0.88,0.95) | 2.01E-07 | |  |  |  |
| 51 | | | | | | cg01752594 | | | DLEU2 | | 13 | | 50696070 | | | N_Shore | Body | |  | | |  | | | 0.94 | | | (0.92,0.96) | 1.84E-07 | |  |  |  |
| 52 | | | | | | cg14642045 | | | UNG | | 12 | | 109538736 | | | S_Shelf | Body | | rs3219221 | | |  | | | 0.94 | | | (0.92,0.96) | 8.63E-08 | |  |  |  |
| 53 | | | | | | cg08400494 | | | CARS2 | | 13 | | 111318490 | | |  | Body | |  | | |  | | | 0.94 | | | (0.92,0.96) | 1.50E-07 | |  |  |  |
| 54 | | | | | | cg09298313 | | |  | | 14 | | 55569959 | | |  |  | |  | | |  | | | 0.90 | | | (0.87,0.94) | 1.35E-07 | |  |  |  |
| 55 | | | | | | cg15961455 | | |  | | 1 | | 23590501 | | |  |  | |  | | |  | | | 0.94 | | | (0.92,0.96) | 1.95E-07 | |  |  |  |
| 56 | | | | | | cg16000989 | | | DCAF4L1 | | 4 | | 41983716 | | | N_Shore | 5'UTR | |  | | |  | | | 0.95 | | | (0.93,0.97) | 3.06E-07 | |  |  |  |
| 57 | | | | | | cg14001486 | | | PRKCH | | 14 | | 61801201 | | |  | Body | |  | | |  | | | 1.08 | | | (1.05,1.11) | 2.25E-07 | |  |  |  |
| 58 | | | | | | cg12810837 | | | CLEC2D | | 12 | | 9822287 | | |  | TSS200 | |  | | |  | | | 1.09 | | | (1.05,1.12) | 1.49E-07 | |  |  |  |
| 59 | | | | | | cg04182865 | | | RNF14 | | 5 | | 141346431 | | | N_Shelf | TSS200 | |  | | |  | | | 0.94 | | | (0.92,0.96) | 6.53E-08 | |  |  |  |
| 60 | | | | | | cg14004161 | | | SNX22 | | 15 | | 64442561 | | | N_Shore | TSS1500 | |  | | |  | | | 0.92 | | | (0.90,0.95) | 3.49E-07 | |  |  |  |
| 61 | | | | | | cg27553947 | | | CLSTN1 | | 1 | | 9819767 | | | N_Shelf | Body | |  | | | rs76639688 | | | 0.93 | | | (0.91,0.96) | 6.65E-08 | |  |  |  |
| 62 | | | | | | cg05163268 | | |  | | 5 | | 180116385 | | |  |  | | rs11738824 | | |  | | | 1.11 | | | (1.07,1.15) | 1.56E-07 | |  |  |  |
| 63 | | | | | | cg25416125 | | | DGKA | | 12 | | 56329615 | | | Island | 5'UTR | |  | | |  | | | 1.12 | | | (1.07,1.17) | 1.02E-07 | |  |  |  |
| 64 | | | | | | cg21815704 | | | GLRX2 | | 1 | | 193075249 | | | S_Shore | TSS1500 | |  | | |  | | | 0.95 | | | (0.93,0.97) | 2.73E-07 | |  |  |  |
| 65 | | | | | | cg07196571 | | | SNX22 | | 15 | | 64442578 | | | N_Shore | TSS1500 | |  | | |  | | | 0.93 | | | (0.90,0.95) | 3.18E-07 | |  |  |  |
| 66 | | | | | | cg12007048 | | | CTSD | | 11 | | 1785701 | | | S_Shore | TSS1500 | |  | | |  | | | 0.87 | | | (0.82,0.92) | 4.97E-07 | |  |  |  |
| 67 | | | | | | cg01554529 | | | FBXO6 | | 1 | | 11722935 | | | N_Shore | TSS1500 | |  | | |  | | | 0.94 | | | (0.92,0.96) | 9.96E-08 | |  |  |  |
| 68 | | | | | | cg27466532 | | | RAPSN | | 11 | | 47471400 | | |  | TSS1500 | |  | | |  | | | 0.92 | | | (0.89,0.95) | 1.90E-07 | |  |  |  |
| 69 | | | | | | cg16971827 | | | CBL | | 11 | | 119177430 | | | N_Shelf | 3'UTR | |  | | |  | | | 1.11 | | | (1.07,1.16) | 2.78E-07 | |  |  |  |
| 70 | | | | | | cg18463607 | | | EXOC1 | | 4 | | 56718320 | | | N_Shore | TSS1500 | |  | | |  | | | 1.10 | | | (1.06,1.14) | 9.20E-08 | |  |  |  |
| 71 | | | | | | cg13879047 | | | GRB10 | | 7 | | 50774217 | | |  | 5'UTR | |  | | |  | | | 0.87 | | | (0.83,0.92) | 6.47E-07 | |  |  |  |
| 72 | | | | | | cg19743406 | | | LHFPL5 | | 6 | | 35771838 | | | N_Shore | TSS1500 | |  | | | rs59697285 | | | 0.90 | | | (0.86,0.94) | 1.88E-06 | |  |  |  |
| 73 | | | | | | cg08930843 | | | ZNF438 | | 10 | | 31182298 | | |  | 5'UTR | |  | | |  | | | 0.95 | | | (0.94,0.97) | 1.70E-07 | |  |  |  |
| 74 | | | | | | cg13451886 | | | SLFN5 | | 17 | | 33568791 | | | N_Shore | TSS1500 | |  | | |  | | | 1.10 | | | (1.06,1.13) | 1.65E-07 | |  |  |  |
| 75 | | | | | | cg21495704 | | | TYROBP | | 19 | | 36399346 | | |  | TSS200 | | | | | rs56006731 | | | 0.93 | | | (0.91,0.96) | 5.43E-08 | |  |  |  |
| 76 | | | | | | cg09674502 | | | GFI1 | | 1 | | 92953279 | | | S_Shore | TSS1500 | |  | | |  | | | 0.93 | | | (0.91,0.96) | 1.11E-07 | |  |  |  |
| 77 | | | | | | cg27073431 | | | CALM2 | | 2 | | 47404636 | | | S_Shore | TSS1500 | |  | | |  | | | 0.94 | | | (0.92,0.96) | 2.11E-07 | |  |  |  |
| 78 | | | | | | cg21159128 | | | SSBP3 | | 1 | | 54693933 | | |  | Body | |  | | |  | | | 0.94 | | | (0.92,0.96) | 1.05E-07 | |  |  |  |
| 79 | | | | | | cg04252203 | | |  | | 3 | | 194696866 | | |  |  | |  | | |  | | | 0.94 | | | (0.91,0.96) | 1.85E-07 | |  |  |  |
| 80 | | | | | | cg13443575 | | | SLFN13 | | 17 | | 33775961 | | | N_Shore | TSS200 | |  | | |  | | | 0.92 | | | (0.89,0.95) | 2.97E-07 | |  |  |  |
| 81 | | | | | | cg05001044 | | | MIR1977 | | 1 | | 567312 | | |  | TSS1500 | |  | | |  | | | 0.93 | | | (0.90,0.95) | 7.96E-08 | |  |  |  |
| 82 | | | | | | cg13716760 | | |  | | 9 | | 15371248 | | |  |  | |  | | |  | | | 0.90 | | | (0.86,0.93) | 1.32E-07 | |  |  |  |
|  | | |  | | | |  | | |  | | |  |  | | | |  | |  | | |  |  | | |  | | |  | | |  |
| (b) 19 blood based top hits (p-value < 5x10^-6^) with adjustment for blood cell compositions from **Additional file 1: Table S4** | | | | | | | | | | | | | | | | | | | | | | | | | | | | | | | | |  |
| 1 | | | cg05001044 | | | | MIR1977 | | | 1 | | | 567312 |  | | | | TSS1500 | |  | | |  | 0.92 | | | (0.89,0.95) | | | 9.07E-08 | | |  |
| 2 | | | cg27211284 | | | |  | | | 2 | | | 200524012 | Island | | | |  | |  | | |  | 1.11 | | | (1.07,1.16) | | | 5.31E-07 | | |  |
| 3 | | | cg05853632 | | | | RNF39 | | | 6 | | | 30043273 | Island | | | | 1stExon | |  | | |  | 1.46 | | | (1.26,1.70) | | | 5.47E-07 | | |  |
| 4 | | | | | cg03830712 | | |  | | 10 | | | 17347391 |  | | | |  | | rs10904927 | | |  | 1.26 | | | (1.15,1.39) | | | 3.17E-06 | | |  |
| 5 | | | cg09136896 | | | | RRP12 | | | 10 | | | 99160663 | N_Shore | | | | Body | | rs10882926 | | |  | 1.29 | | | (1.16,1.42) | | | 8.90E-07 | | |  |
| 6 | | | cg05683049 | | | | CDH10 | | | 5 | | | 24645093 |  | | | | TSS200 | |  | | |  | 1.13 | | | (1.07,1.19) | | | 1.33E-06 | | |  |
| 7 | | | cg18809855 | | | | CGNL1 | | | 15 | | | 57668539 | Island | | | | TSS200 | |  | | |  | 1.17 | | | (1.10,1.26) | | | 3.82E-06 | | |  |
| 8 | | | cg13286582 | | | | CDC42EP3 | | | 2 | | | 37883934 |  | | | | 5'UTR | |  | | |  | 0.90 | | | (0.86,0.94) | | | 5.63E-06 | | |  |
| 9 | | | cg10248492 | | | |  | | | 1 | | | 228647248 | S_Shore | | | |  | |  | | |  | 1.17 | | | (1.10,1.25) | | | 2.22E-06 | | |  |
| 10 | | | cg03958663 | | | |  | | | 6 | | | 41262467 |  | | | |  | |  | | |  | 0.88 | | | (0.83,0.93) | | | 4.23E-06 | | |  |
| 11 | | | cg09040699 | | | | EIF3F | | | 11 | | | 8009242 | Island | | | | 1stExon | |  | | |  | 1.24 | | | (1.13,1.35) | | | 2.49E-06 | | |  |
| 12 | | | cg01685644 | | | | MBLAC1 | | | 7 | | | 99724788 | Island | | | | 5'UTR | |  | | |  | 1.21 | | | (1.11,1.31) | | | 1.02E-05 | | |  |
| 13 | | | cg25679366 | | | | ABCB4 | | | 7 | | | 87104879 | Island | | | | 5'UTR | | rs34690247 | | | | 1.33 | | | (1.17,1.51) | | | 9.53E-06 | | |  |
| 14 | | | cg26544742 | | | |  | | | 6 | | | 27533825 |  | | | |  | |  | | |  | 1.07 | | | (1.04,1.11) | | | 2.06E-05 | | |  |
| 15 | | | cg07391218 | | | | HPCAL1 | | | 2 | | | 10442879 | Island | | | | TSS1500 | |  | | |  | 1.62 | | | (1.32,1.99) | | | 5.09E-06 | | |  |
| 16 | | | cg26560222 | | | | DMRTA2 | | | 1 | | | 50888826 | Island | | | | 1stExon | |  | | |  | 1.12 | | | (1.07,1.18) | | | 8.91E-06 | | |  |
| 17 | | | cg05624199 | | | |  | | | 19 | | | 41168318 | N_Shore | | | |  | |  | | |  | 0.92 | | | (0.89,0.95) | | | 8.58E-06 | | |  |
| 18 | | | cg19342159 | | | | C19orf60 | | | 19 | | | 18699433 | Island | | | | TSS200 | |  | | |  | 1.53 | | | (1.25,1.87) | | | 2.74E-05 | | |  |
| 19 | | | cg11266874 | | | | ARL13B | | | 3 | | | 93699352 | Island | | | | Body | |  | | |  | 1.40 | | | (1.21,1.61) | | | 3.18E-06 | | |  |
|  | |  |  |  |  |  |  |  |  |  |  |  |  |  |  |  |  |  |  |  |  |  |  |  |  |  |  |  |  |  |  |  |  |
| (c) 5 saliva based top hits (p-value < 10^-7^) from **Table 2** | | | | | | | | | | | | | | | | | | | | | | | | | | | | | | | |  |  |
| 1 | | | | cg15133963 | | | FTHL3 | | | 2 | | 27616316 | | | Island | | | Body | |  |  | | | | | 0.82 | | (0.76,0.88) | | 1.71E-07 | | | |
| 2 | | | | cg01820192 | | | C21orf125 | | | 21 | | 44869762 | | |  | | | TSS200 | |  |  | | | | | 0.85 | | (0.80,0.91) | | 4.23E-07 | | | |
| 3 | | | | cg22275276 | | |  | | | 6 | | 33973531 | | |  | | |  | |  |  | | | | | 0.81 | | (0.75,0.88) | | 6.11E-07 | | | |
| 4 | | | | cg11748881 | | | FTH1 | | | 11 | | 61734830 | | | Island | | | 1stExon | |  | rs11554886 | | | | | 0.87 | | (0.82,0.92) | | 1.04E-06 | | | |
| 5 | | | | cg24742912 | | | MYBPH | | | 1 | | 203146346 | | |  | | | TSS1500 | | rs7538338 |  | | | | | 0.75 | | (0.67,0.84) | | 1.11E-06 | | | |
| Chr.: Chromosome, bp: base pair, TSS: transcription start site, TSS1500: within 1500 bps of a TSS, TSS200: within 200 bps of a TSS, UTR: untranslated region, SNPs: listing dbSNP entries within a probe, SNPs_10: listing dbSNP entries within 10 bp of the CpG site. | | | | | | | | | | | | | | | | | | | | | | | | | | | | | | | | | |

**Table S12. Correlations between 24 CpGs on the LARS2 gene.**The Pearson’s correlation coefficients and the corresponding p-values are shown for each cell. Red color indicates positive correlations while green color indicates negative correlations.

(a) Using the DNA methylation data in blood that were adjusted for age and sex


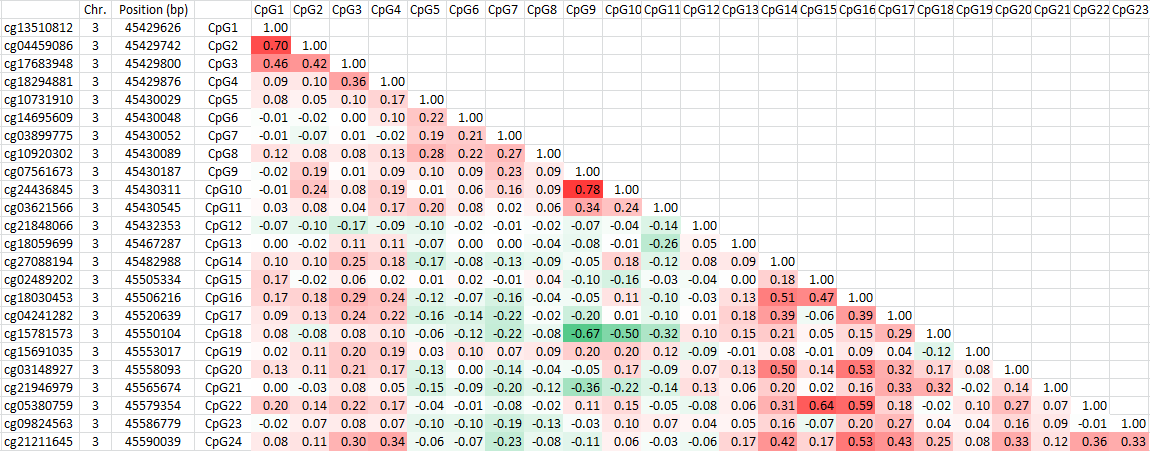


(b) Using the DNA methylation data in blood that were adjusted for age, sex, and cell composition


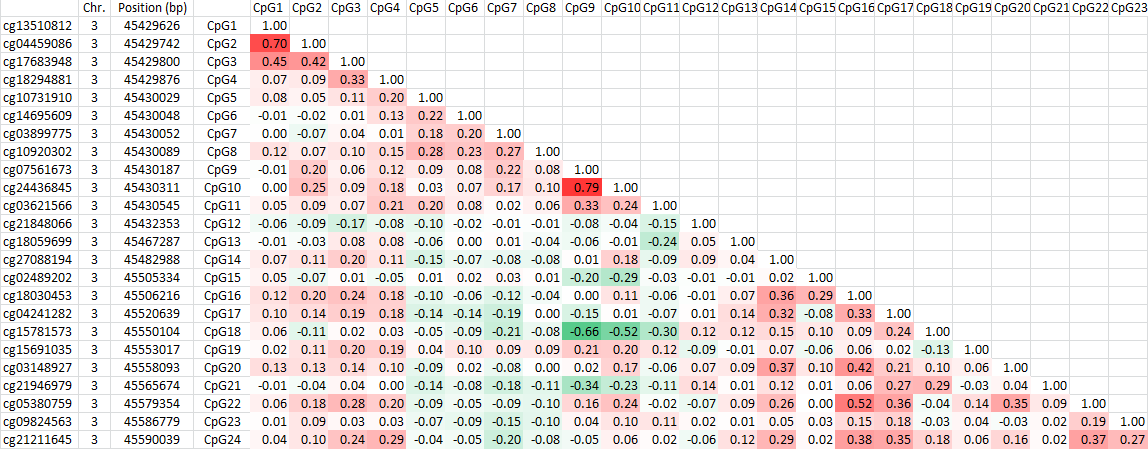


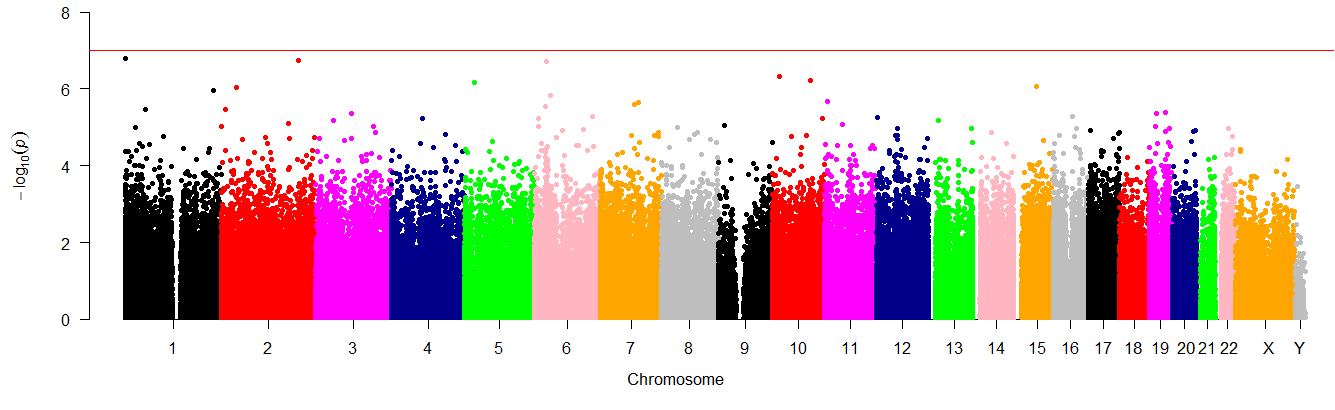
(a)


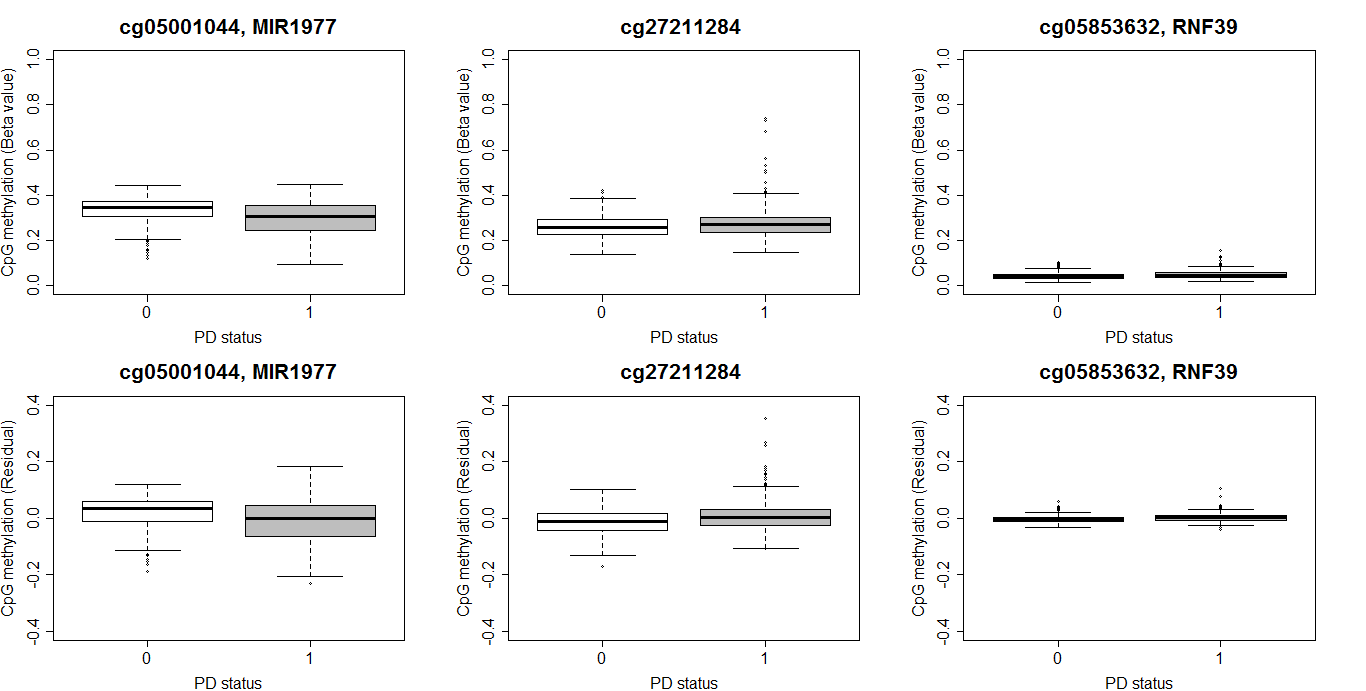

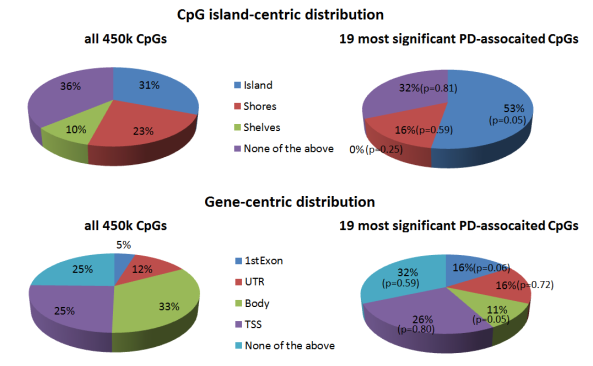
(b) (c)

**Figure S1. EWAS results for PD and blood based DNA methylation with cell composition adjustment.** Differential methylation associated with PD status in 508 PEG1 subjects of European ancestry adjusting for age, gender, and blood cell composition. (a) Manhattan plot of p-values adjusted for age and gender (red line: p-value threshold of 10^-7^). (b) Distributions of CpGs relative to CpG island and gene regions for all 450k CpGs on the microarray and the 19 most significant coffee-associated CpGs listed in **Additional file 1: Table S4**. (c) Distribution of DNA methylation levels for the top 3 most significant PD-associated CpGs by PD status (1=PD).


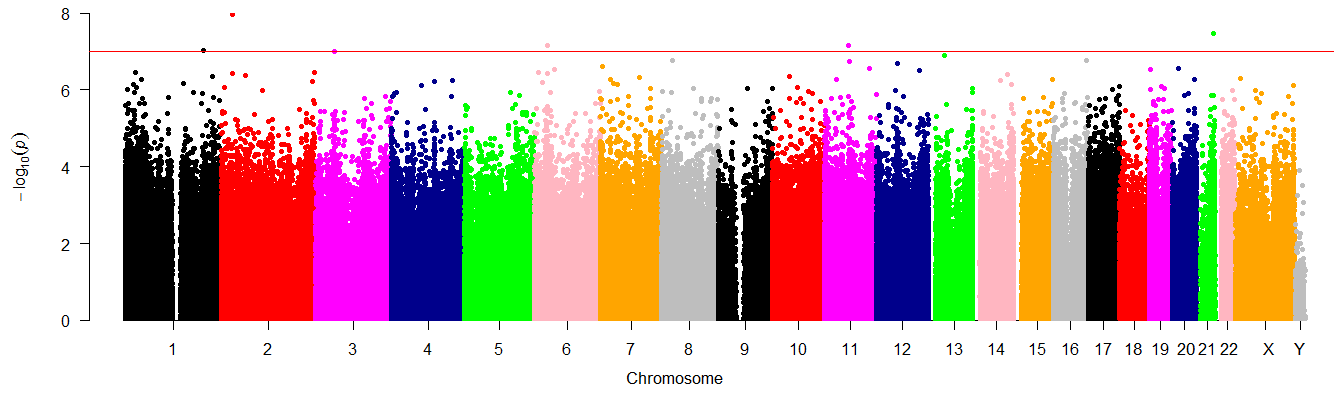
(a)


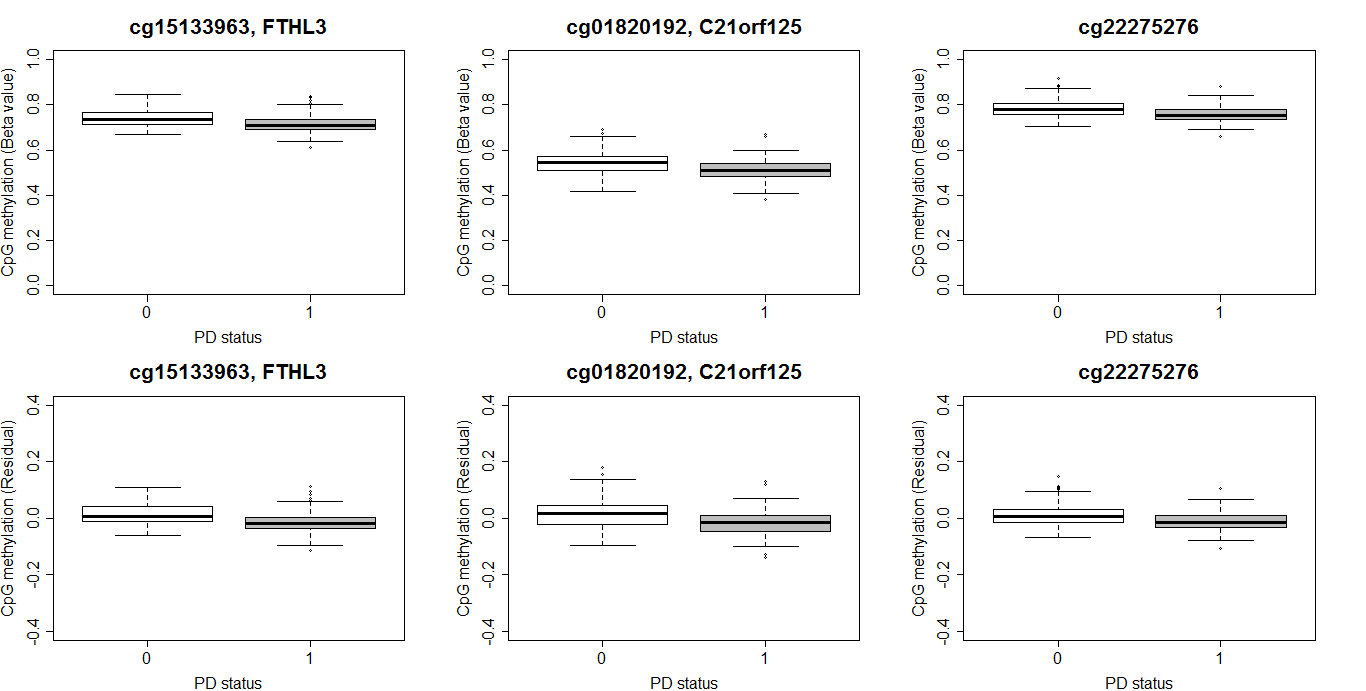
(b) (c)


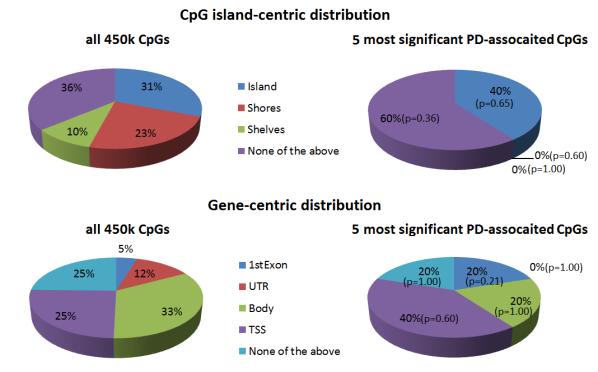


**Figure S2.** **EWAS results for PD and saliva based DNA methylation.** Differential methylation associated with PD status in 259 PEG2 subjects adjusting for age, gender and race. (a) Manhattan plot of p-values adjusted for age and gender (red line: p-value threshold of 10^-7^). (b) Distributions of CpGs relative to CpG island and gene regions for all 450k CpGs on the microarray and the 5 most significant coffee-associated CpGs listed in **Table 2**. (c) Distribution of DNA methylation levels in the top 3 most significant PD-associated CpGs by PD status (1=PD).

**Figure S3. WGCNA results for PD and blood based DNA methylation adjusting for age.** Correlations of module eigengenes (ME) with PD status and other traits in 508 PEG1 subjects of European ancestry adjusting for age. The rows represent ME and its color. The columns represent clinical traits. The Pearson’s correlation coefficients and the corresponding p-values are shown for each cell. Red color indicates positive correlations while green color indicates negative correlations. (a) ME1-25. (b) ME26-50. (c) ME51-76.

(a)


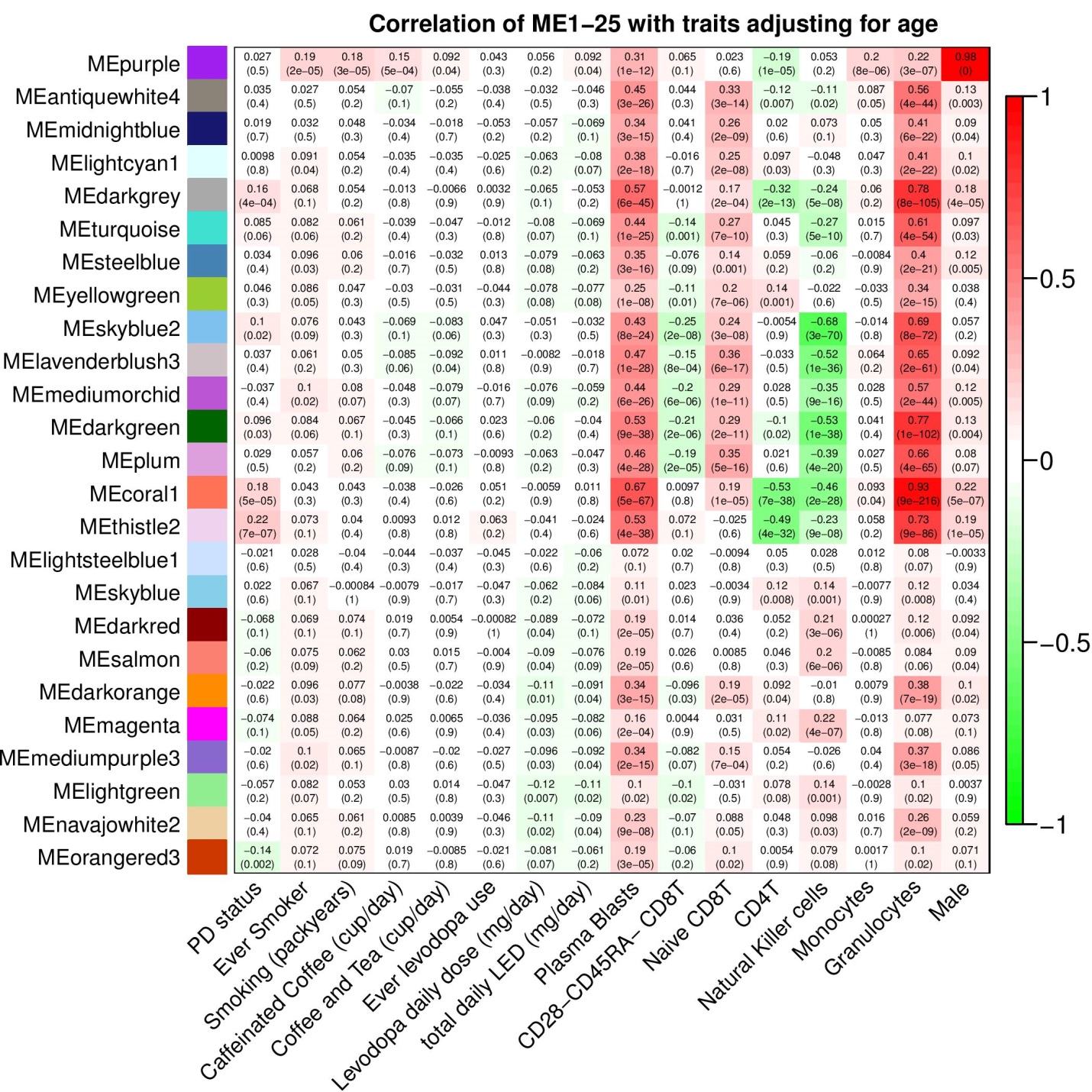


(b)
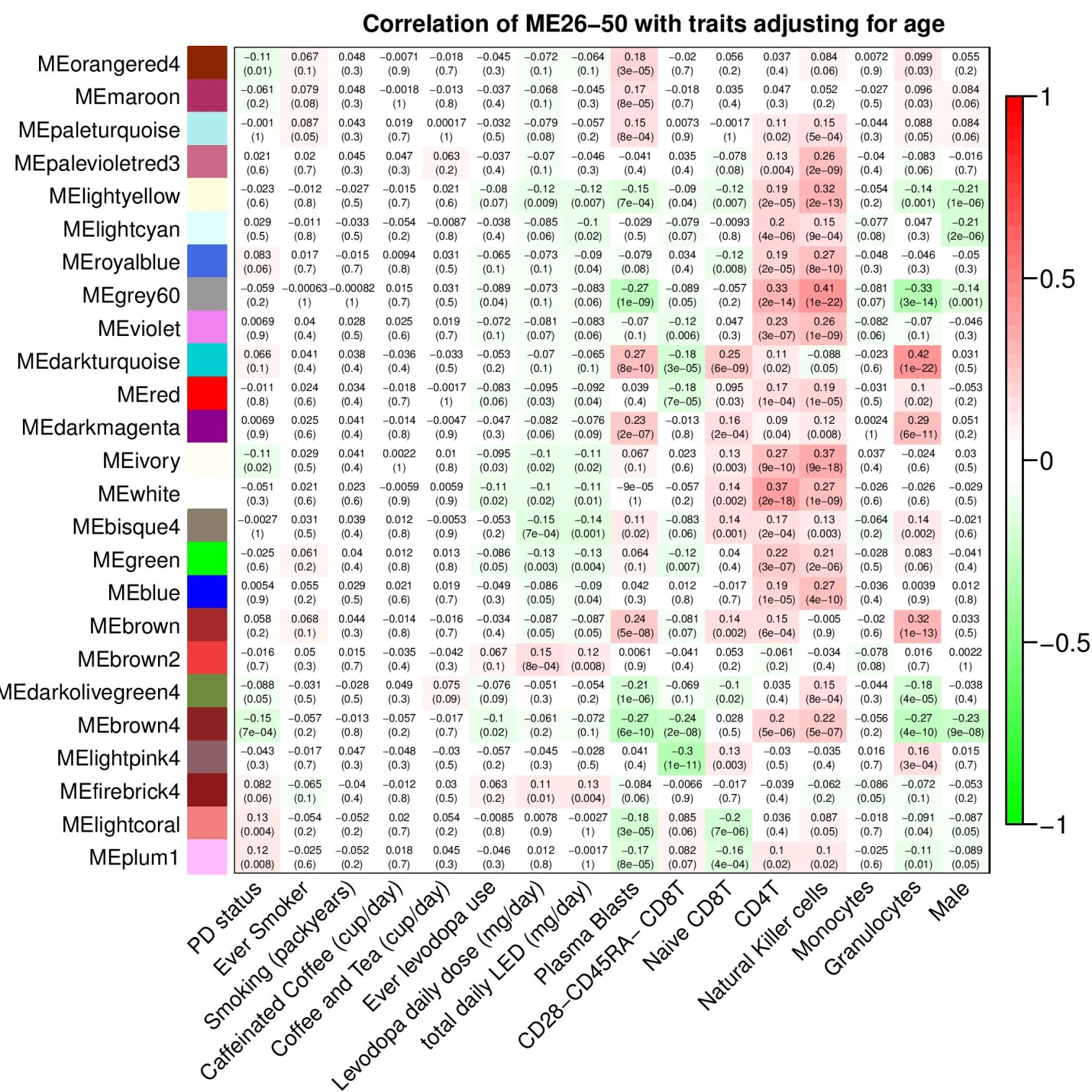


(c)
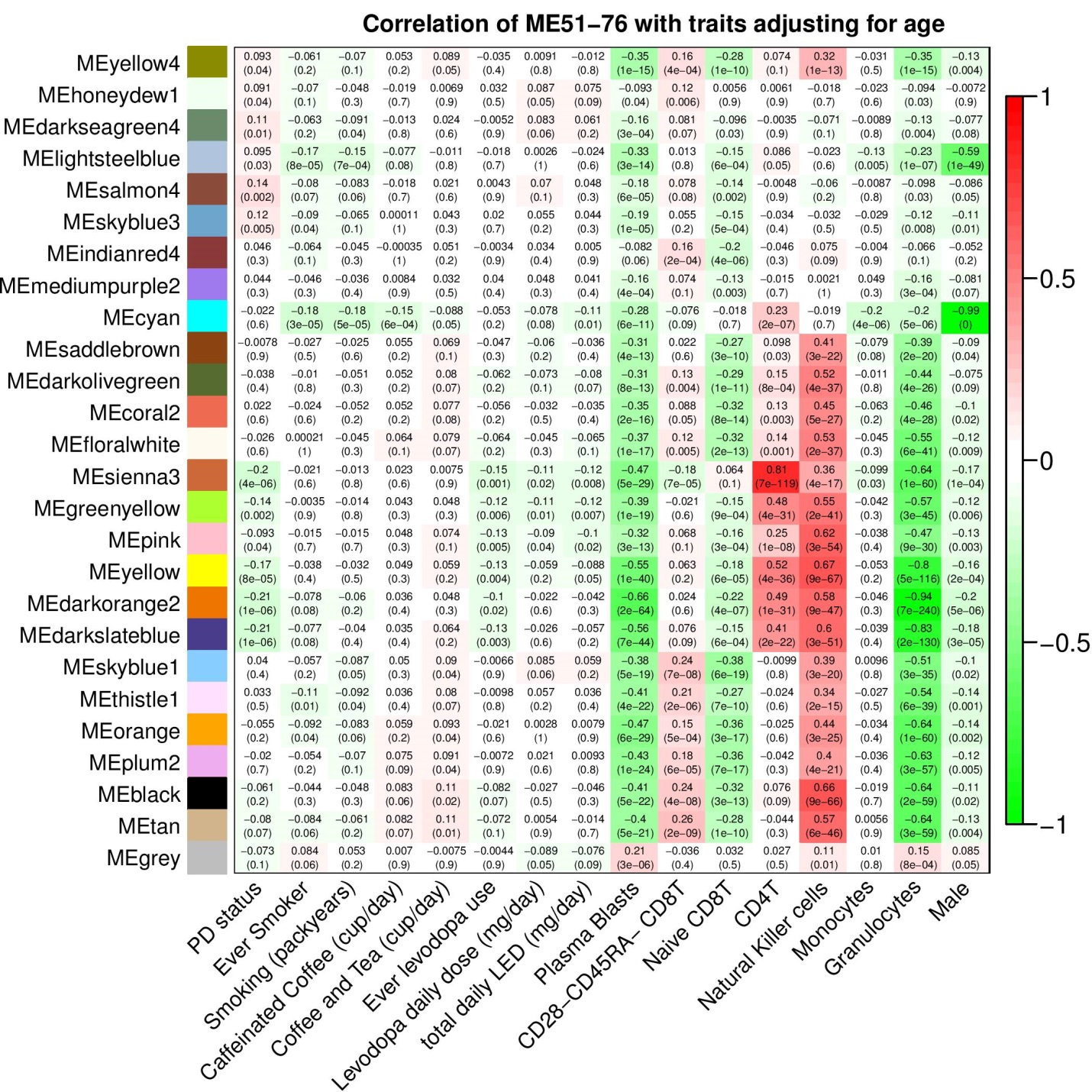


**Figure S4. WGCNA results for PD and blood based DNA methylation with cell composition adjustment.** Correlations of module eigengenes (ME) with PD status and other traits in 508 PEG1 subjects of European ancestry adjusting for age, gender, and cell composition. The rows represent ME and its color. The columns represent clinical traits. The Pearson’s correlation coefficients and the corresponding p-values are shown for each cell. Red color indicates positive correlations while green color indicates negative correlations. (a) ME1-25. (b) ME26-50. (c) ME51-75. (d) ME76-97.

(a)

**
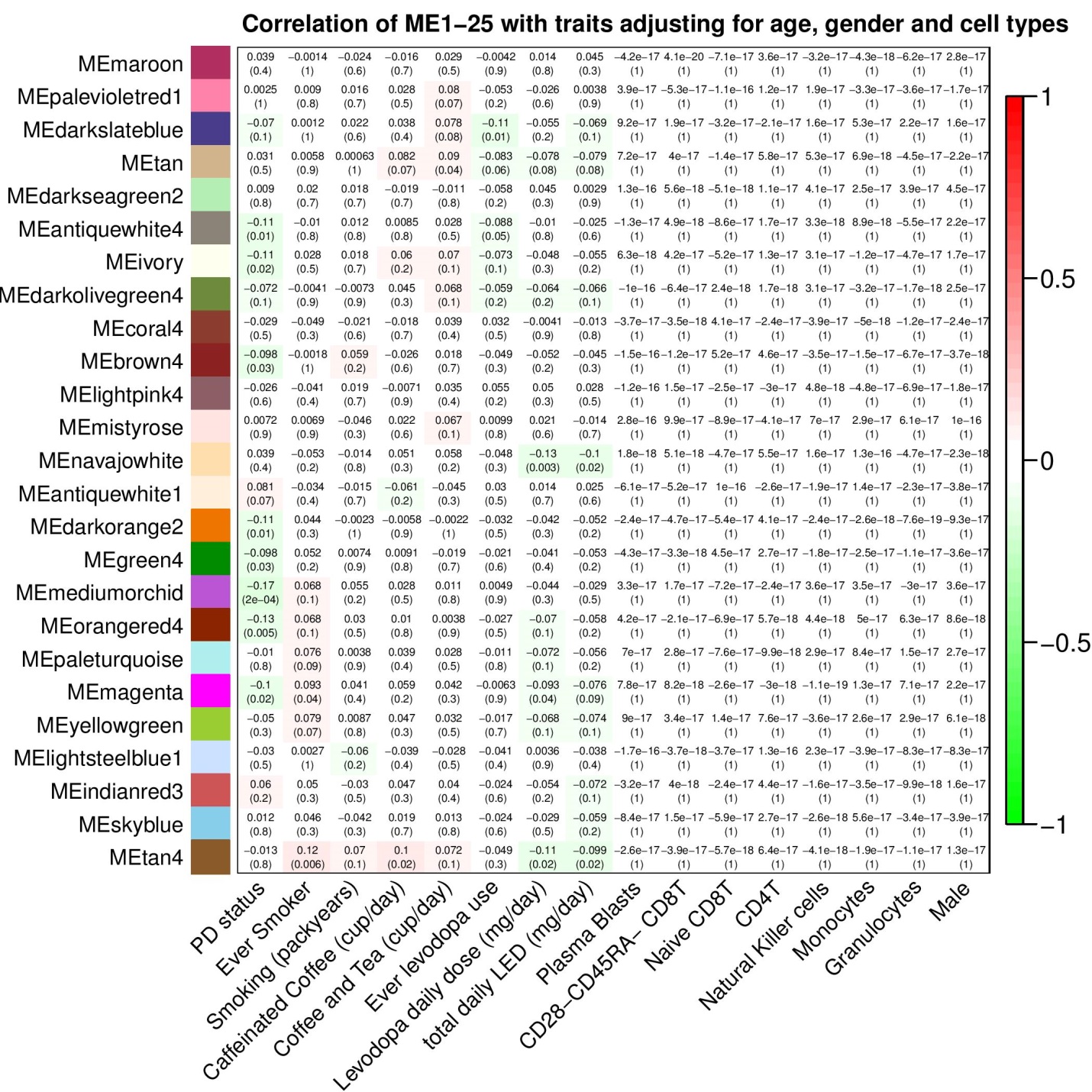
**

(b)

**
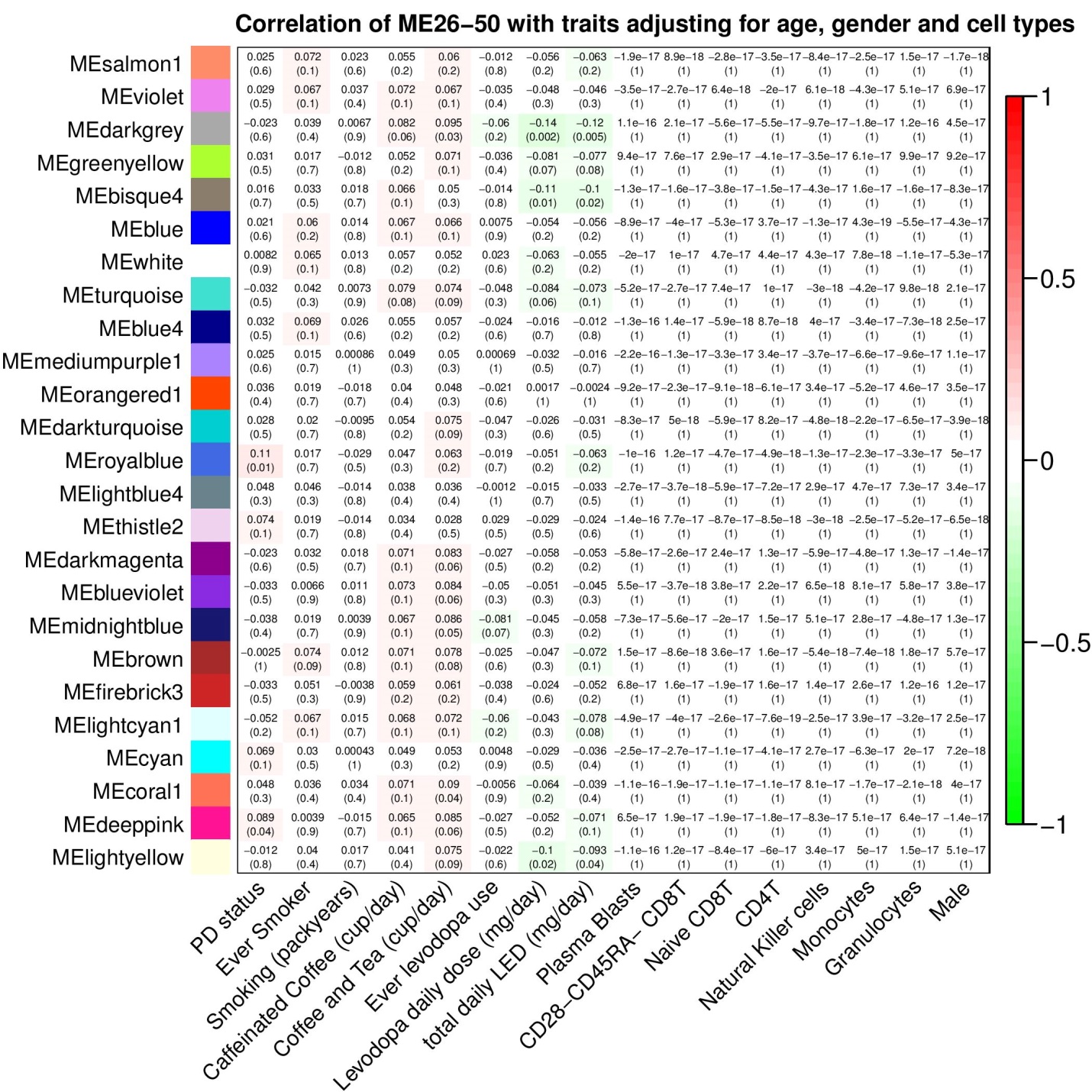
**

(c)**
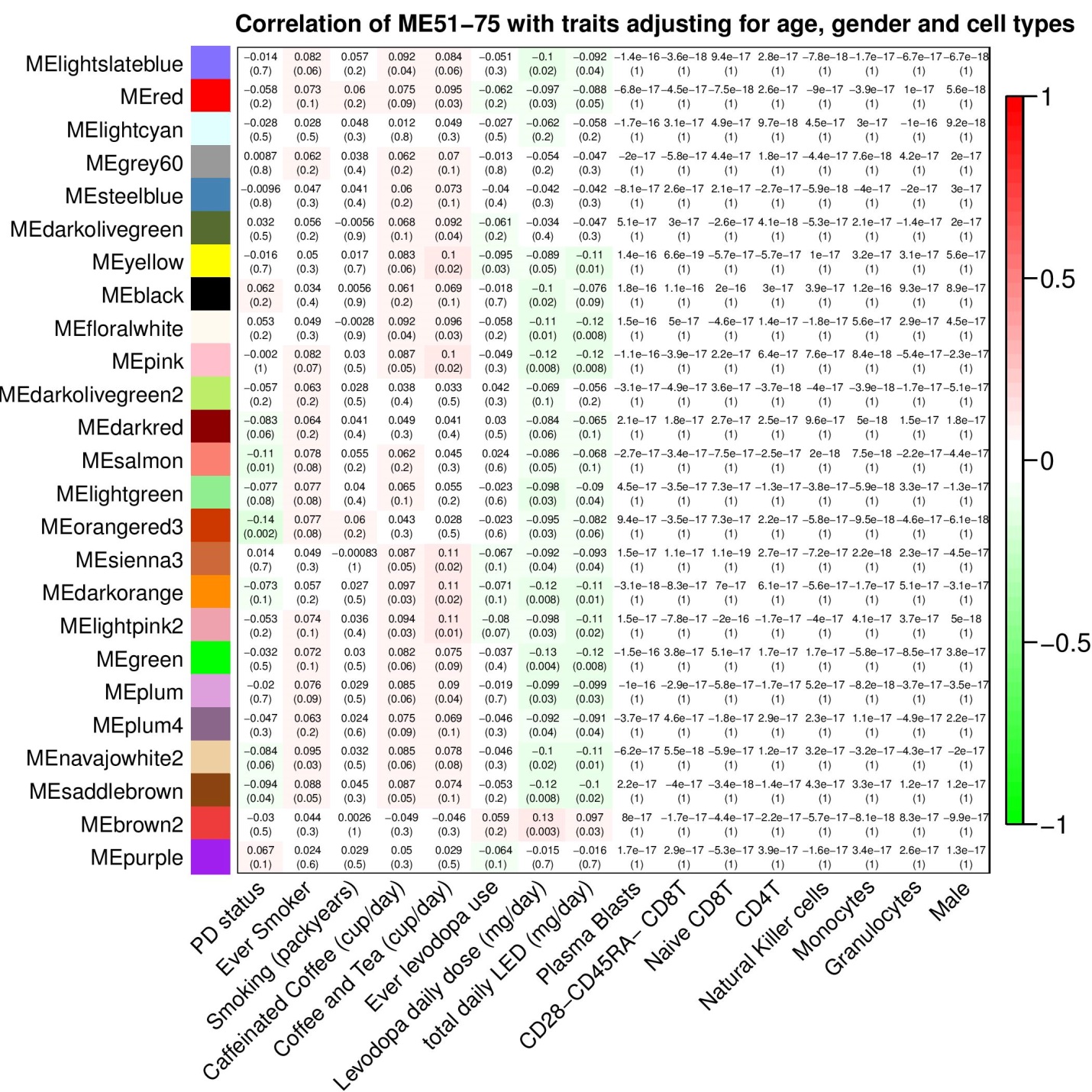
**

(d)**
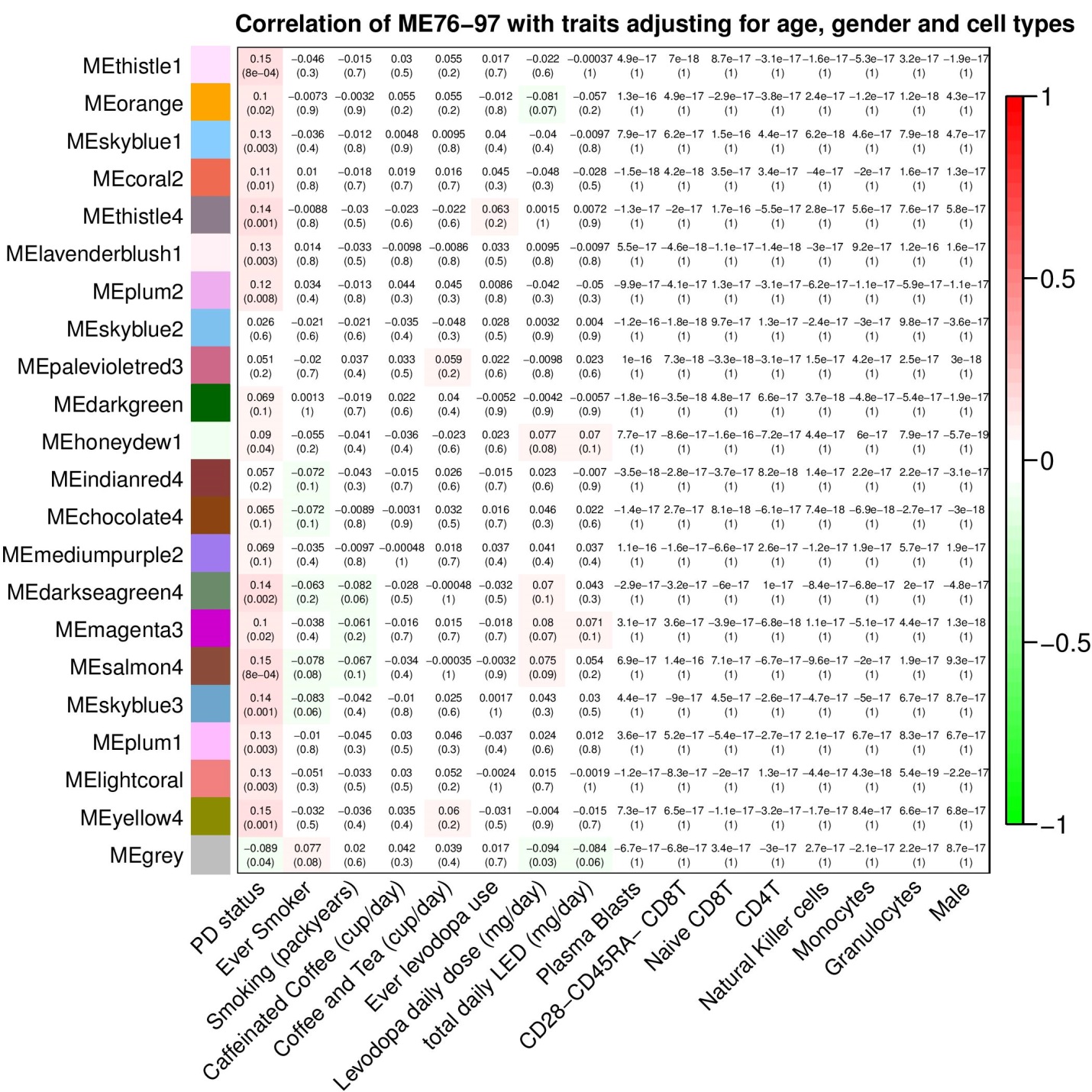
**

**Figure S5. A summary of biological pathways identified in this study.** These biological pathways are involved in PD pathogenesis or cell dysfunction.

**
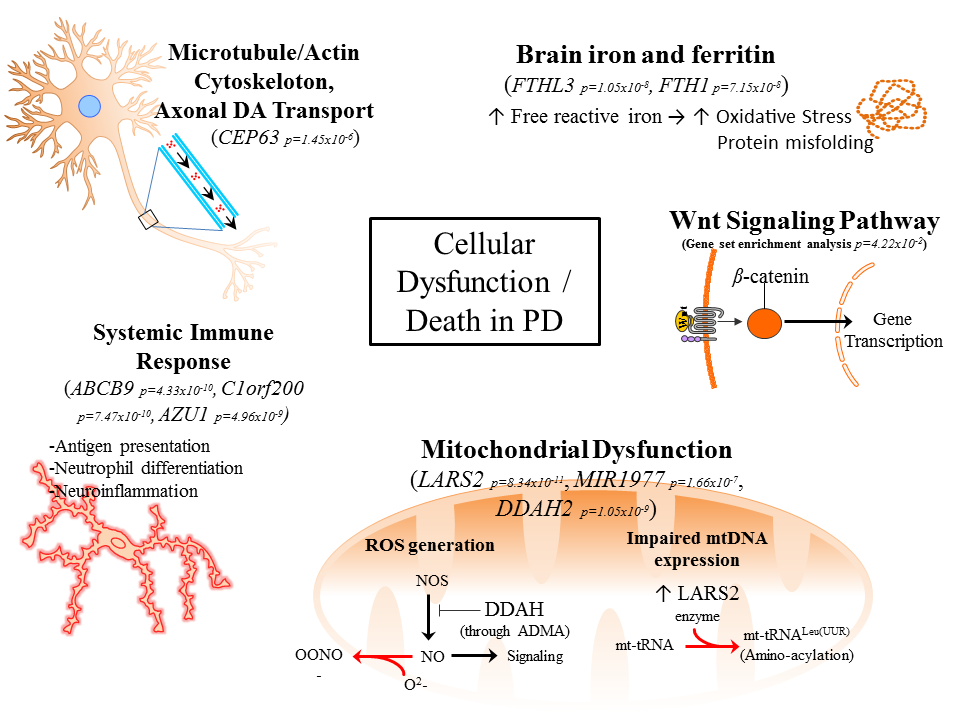
**

**Figure S6. WGCNA results for PD and blood based DNA methylation.** Correlations of module eigengenes (ME) with PD status and other traits in 508 PEG1 subjects of European ancestry. The rows represent ME and its color. The columns represent clinical traits. The Pearson’s correlation coefficients and the corresponding p-values are shown for each cell. Red color indicates positive correlations while green color indicates negative correlations. (a) ME1-25. (b) ME26-50. (c) ME51-75.

(a)

**
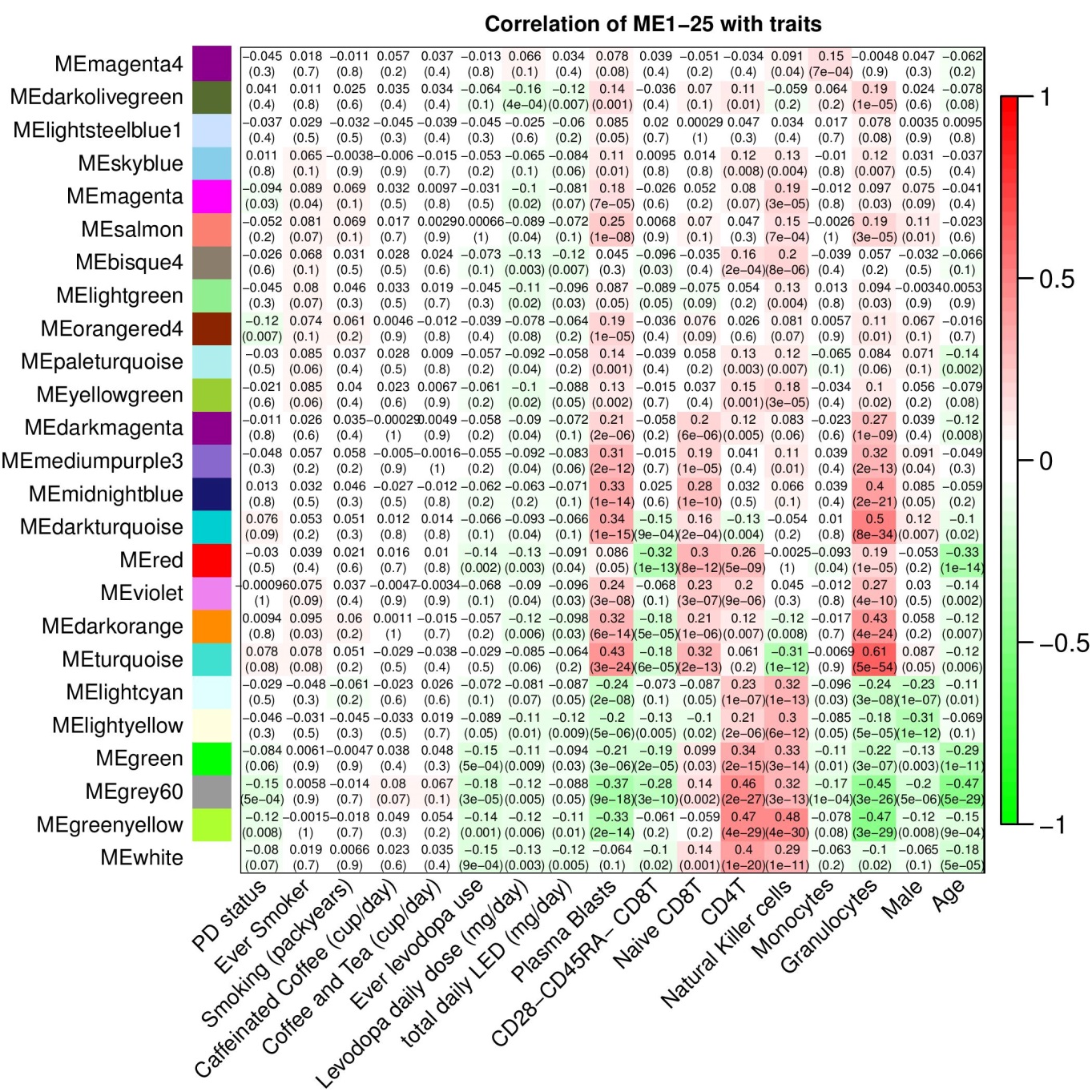
**

(b)

**
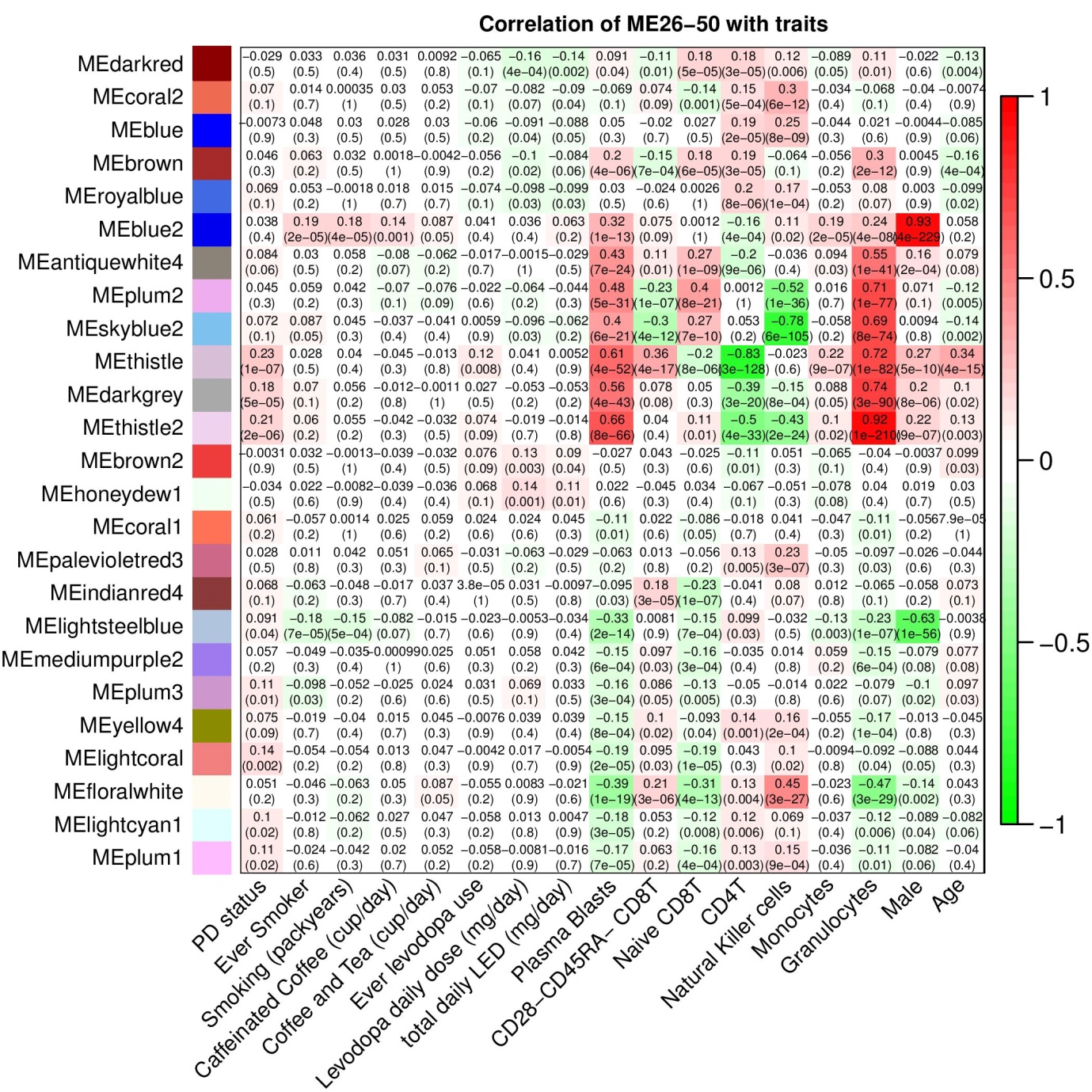
**

(c)**
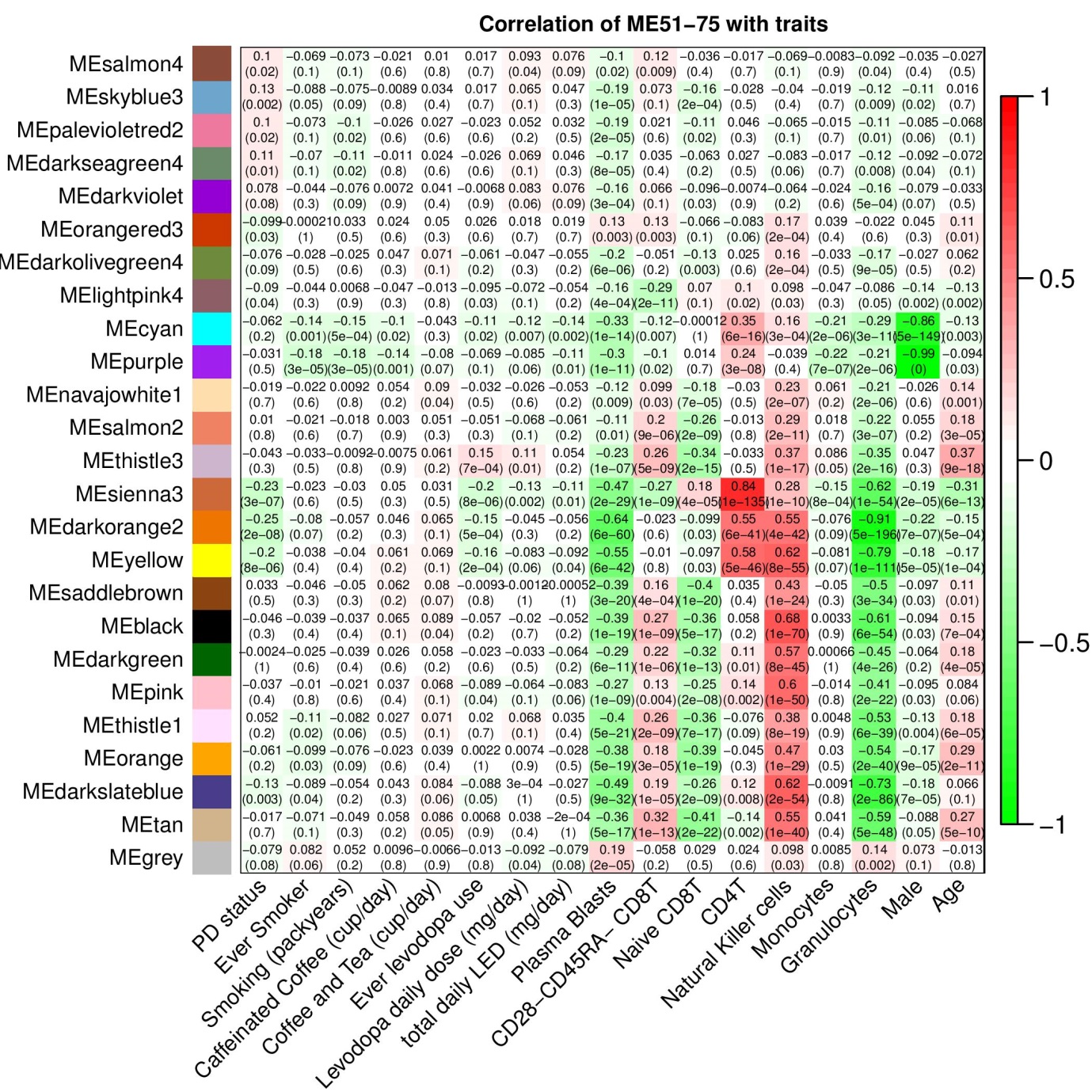
**

**Figure S7. WGCNA results for PD and saliva based DNA methylation.** Correlations of module eigengenes (ME) with PD status and other traits in 259 PEG2 subjects adjusting for age, gender, and race. The rows represent ME and its color. The columns represent clinical traits. The Pearson’s correlation coefficients and the corresponding p-values are shown for each cell. Red color indicates positive correlations while green color indicates negative correlations. (a) ME1-25. (b) ME 26-48.

(a)

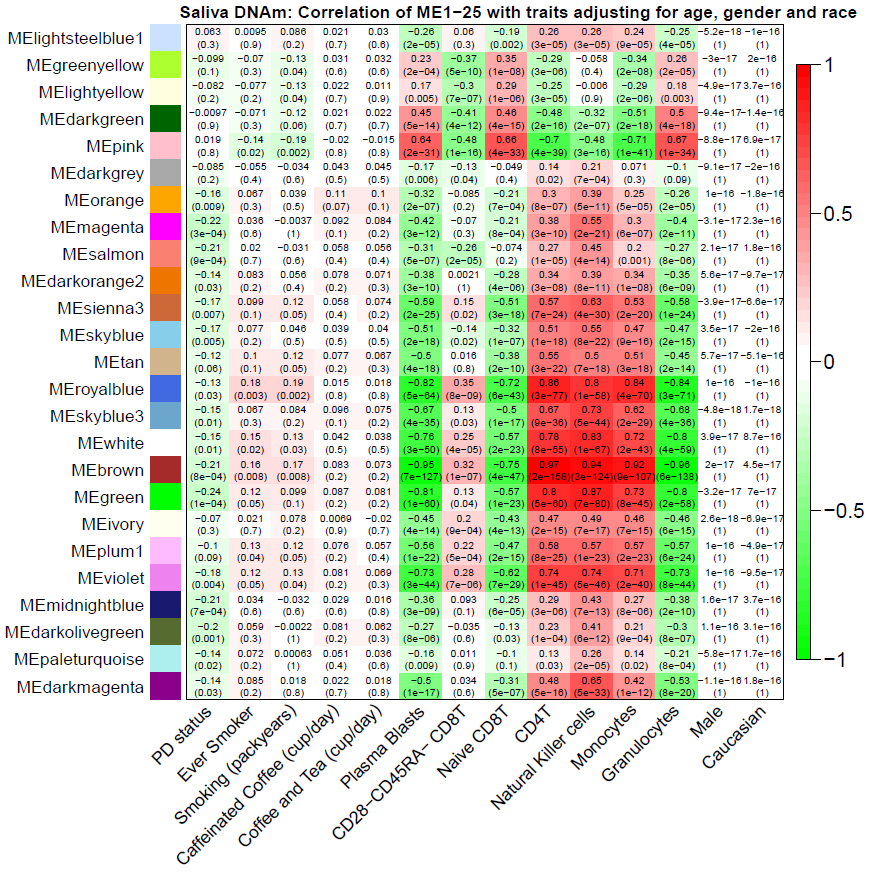


(b)


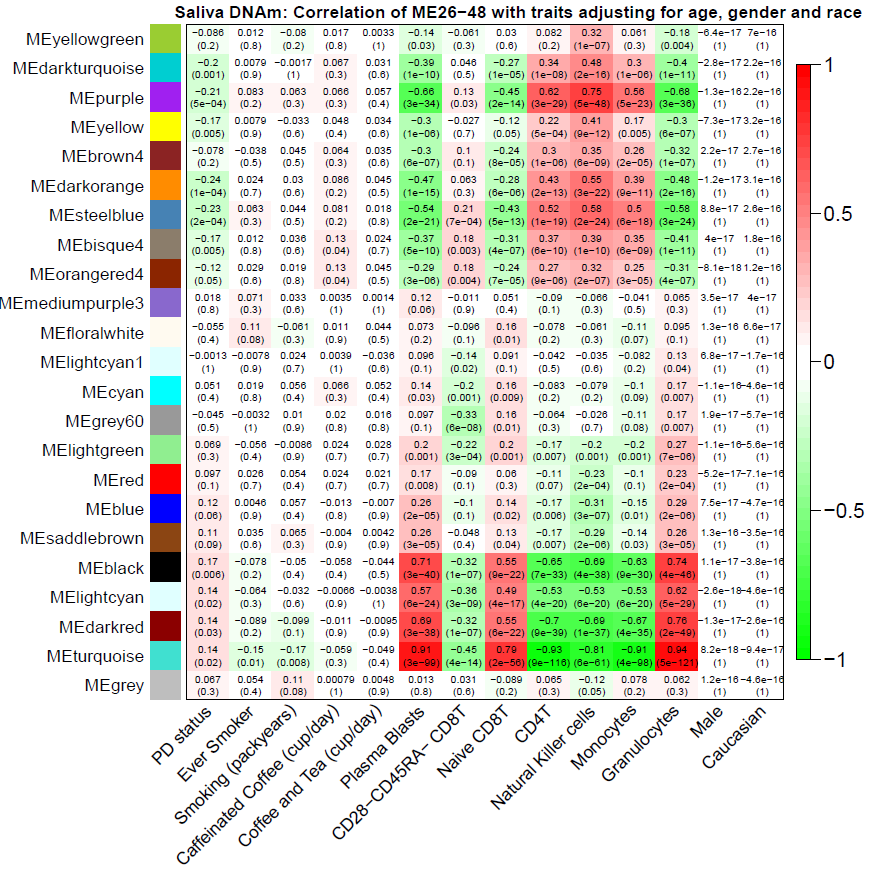

Supplement: Additional file 1: — Supplementary tables and figures that show the following: characteristics of study participants, results of EWAS, WGCNA, meta-analyses, and gene set enrichment analyses. (DOCX 8897 kb) [file 13073_2017_466_MOESM1_ESM.docx]
